# BM-derived mesenchymal stem cell microvesicles protect enteric neural precursor cells and alleviate diabetes-associated enteric neuropathy

**DOI:** 10.1172/JCI192437

**Published:** 2026-03-16

**Authors:** Huiying Shi, Hailing Yao, Yilin Liu, Mengke Fan, Sicheng Cai, Shizhao Xu, Chen Jiang, Yurui Zhang, Weiwei Jiang, Wei Qian, Rong Lin

**Affiliations:** Department of Gastroenterology, Union Hospital, Tongji Medical College, Huazhong University of Science and Technology, Wuhan, China.

**Keywords:** Gastroenterology, Neuroscience, Neuronal stem cells

## Abstract

Enteric nervous system (ENS) injury, characterized by progressive degeneration of enteric neurons and glial cells, is a common diabetic complication with no effective cure beyond symptomatic management. Enteric neural precursor cells (ENPCs) play a key role in maintaining neurogenesis and gliogenesis within the adult ENS. Here, we demonstrate that bone marrow mesenchymal stem cell–derived microvesicles (BMSC-MVs) alleviate diabetic ENS injury. In both diabetic patients and mouse models, gastrointestinal transit was delayed, ENS structure was impaired, and neurogenesis and gliogenesis from ENPCs were elevated yet remained functionally insufficient. Transcriptomic profiling revealed activation of ER stress and the pro-apoptotic PERK branch of the unfolded protein response in ENPCs. BMSC-MVs homed to the colon, were internalized by ENPCs, and suppressed ER stress, thereby enhancing functional neurogenesis and gliogenesis, restoring ENS structure, and improving gastrointestinal motility. Mechanistically, vinculin on BMSC-MVs bound talin-1 on ENPCs, activating the ERK pathway to suppress diabetic ER stress. These results identify BMSC-MVs as a promising cell-free therapeutic strategy for diabetic ENS injury.

## Abstract 2

BMSCs-MVs Alleviate Diabetes-Induced ENS Injury by VCL-TLN1-ERK axis to Suppress ER Stress in ENPCs

## Graphical abstract

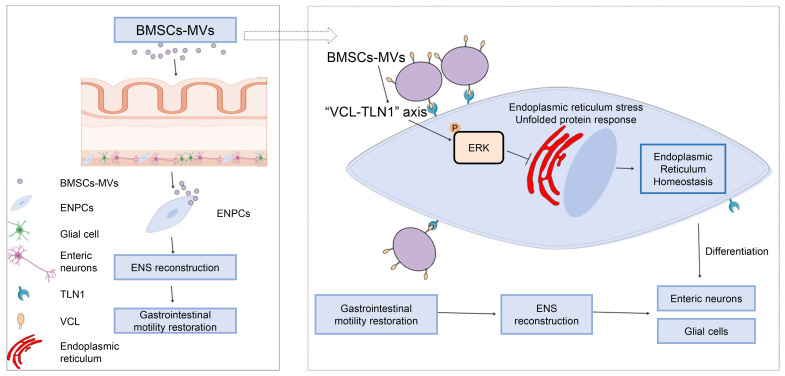

## Introduction

Diabetes mellitus is a prevalent global disorder, affecting approximately 13%–15% of the world’s population (1). Its incidence continues to rise, with projections indicating 600 million cases by 2035 and 783 million by 2045 (2, 3). In parallel with this escalating prevalence, diabetes-related complications have emerged as a critical public health burden, imposing substantial impacts on both individuals and society (4). Among the complications, injury to the enteric nervous system (ENS) is particularly common, occurring in approximately 50%–75% of diabetic patients (5, 6). This condition can affect the entire gastrointestinal tract from the esophagus to the anus and manifests clinically as a spectrum of motility disorders, including esophageal dysmotility, gastroparesis, diarrhea, constipation, and abdominal pain (7, 8). Despite advances in glycemic management, current treatments for diabetic ENS injury, such as prokinetic agents, antiemetics, and laxatives, merely alleviate symptoms without addressing the underlying pathology (9), highlighting the need for further research and development of novel therapeutic approaches to address this substantial medical challenge.

The ENS comprises a complex network of neurons and glial cells that govern gastrointestinal function. Recent evidence has shown that enteric neural precursor cells (ENPCs), located in the myenteric plexus of the gastrointestinal tract, possess the ability to differentiate into neurons or glial cells and contribute to ENS reconstitution following physical or inflammatory injury (10). Kulkarni et al. found that ENPCs were capable of rapid neurogenesis in vivo to maintain a dynamic balance of enteric neurons in healthy adult ENS (11). In a related context, D’Errico et al. demonstrated that estrogen receptor β agonist could activate ENPC-derived neogenesis, facilitating neuronal recovery after injury (12). In addition, our previous study revealed that promoting the differentiation of ENPCs could remodel ENS and restore gastric motility in mice with pylorus denervation (13). Collectively, based on these findings, the development of novel pharmacological agents targeting ENPCs will be essential to treat ENS-related disorders.

Bone marrow mesenchymal stem cells (BMSCs) have shown notable potential in promoting nerve repair following injury or disease (14). Initially, their therapeutic benefits were primarily attributed to direct differentiation and replacement of damaged tissues; however, emerging evidence indicates that these effects are largely mediated through paracrine secretion of bioactive molecules (15, 16). Among these secreted factors, extracellular vesicles (EVs), including exosomes and microvesicles (MVs), have garnered increasing attention as key mediators of BMSC-driven neuroprotection (17–19). These bilayer membranous structures, ranging from 30 to 2,000 nm in diameter, not only replicate many of the therapeutic properties of BMSCs but also offer advantages such as higher stability, lower immunogenicity, and reduced ethical concerns (20, 21). Supporting this, our previous work demonstrated that while BMSCs effectively reversed diabetes-induced gastrointestinal motility dysfunction and ENS injury, they did not differentiate into enteric neurons or glial cells, implying the involvement of a secretory mechanism (22).

Therefore, this study aimed to investigate the therapeutic effects of BMSC-MVs on the ENS and ENPCs in a mouse model of diabetes-induced ENS injury and to further elucidate the underlying mechanisms.

## Results

### ER stress and unfolded protein response were activated in ENPCs under diabetic conditions.

**Figure 1:**
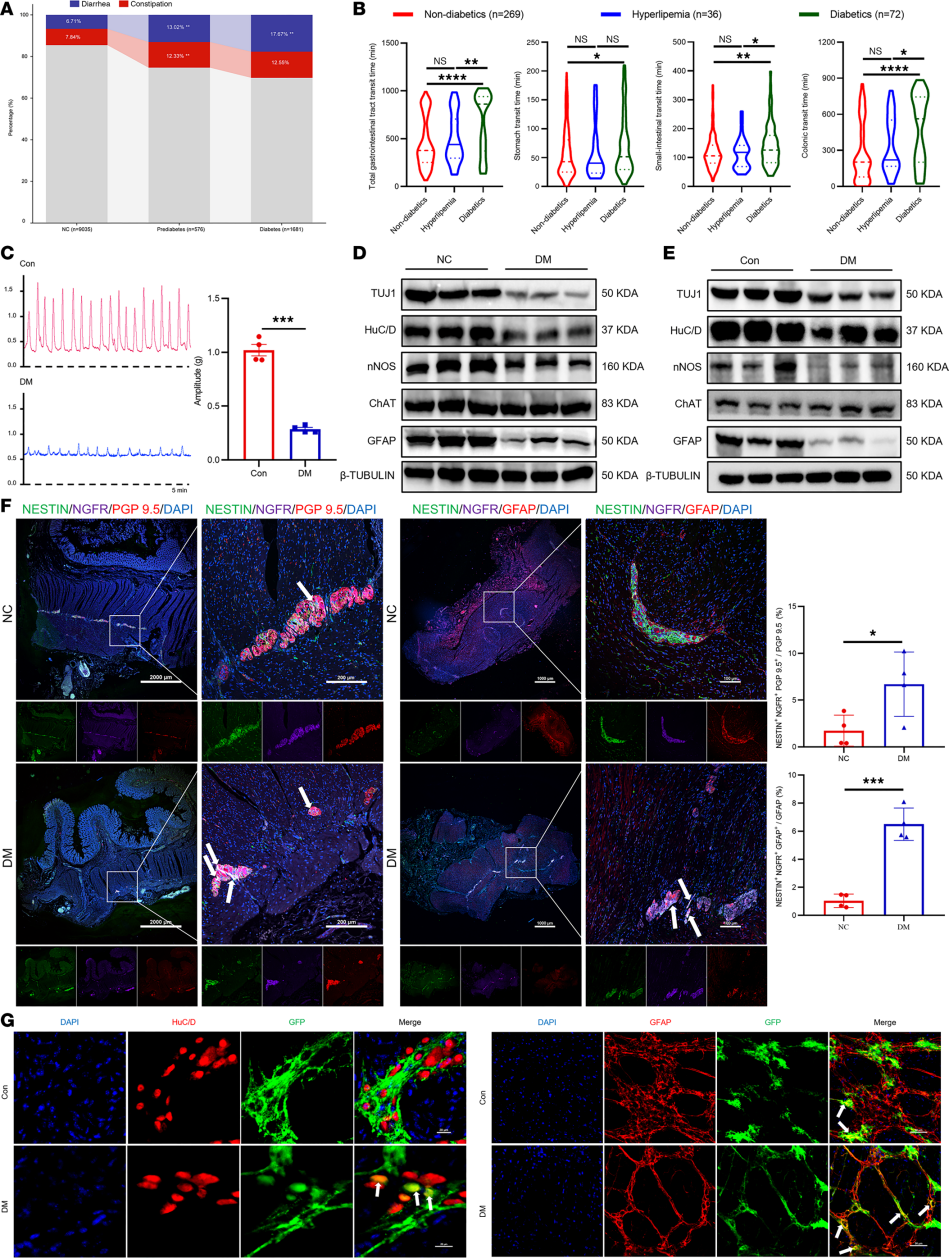
Diabetes-associated gastrointestinal motility dysfunction and ENS impairment in humans and mice. (**A**) Incidence of chronic constipation and diarrhea in normal (NC), prediabetic, and diabetic individuals. (**B**) Gastrointestinal transit time (total, gastric, small intestinal, and colonic) in control, hyperlipidemic, and diabetic groups. (**C**) Representative images and quantitative analysis of spontaneous colonic muscle strip contractility in control and diabetic mice (DM; 5 minutes). *n* = 4. (**D** and **E**) Western blot analysis of TUJ1, HuC/D, nNOS, ChAT, and GFAP expression in human (**D**) and mouse (**E**) colon tissues. *n* = 6. (**F**) Representative immunofluorescence images and quantification of ENPC-derived (NESTIN^+^NGFR^+^) neurons and glial cells in the transverse section of colon from human samples. Scale bars: 2,000, 1,000, 200, and 100 μm. *n* = 4. (**G**) Representative immunofluorescence images of ENPC-derived (GFP^+^) neurons and glial cells in mouse colonic longitudinal muscle-myenteric plexus. Scale bars: 20 μm (left), 50 μm (right). *n* = 4. Data are presented as mean ± SD. Statistical significance was determined by unpaired 2-tailed Student’s *t* test (**A**, **C**, and **F**) and 1-way ANOVA with Tukey’s multiple-comparison test (**B**). *P < 0.05, **P < 0.01, ***P < 0.001, ****P < 0.0001; ns, *P* > 0.05.

We analyzed data from the 2005–2010 National Health and Nutrition Examination Survey database and found that the incidence of chronic diarrhea and constipation was significantly higher in patients with prediabetes (25.35%) and diabetes (30.22%) compared with normal controls (14.55%) (Figure 1A). Consistently, a retrospective analysis of patients undergoing magnetically controlled capsule endoscopy at our center (2019–2024) revealed markedly prolonged gastrointestinal transit times (including gastric, small intestinal, colonic, and whole-gut transit) in individuals with diabetes (Figure 1B). Parallel ex vivo experiments demonstrated weakened spontaneous contractions of colonic muscle strips in diabetic mice (Figure 1C).

Histologically, the ENS network exhibited marked structural damage in the colon of both diabetic humans and mice, characterized by decreased expression of β III-tubulin (TUJ1; nerve fiber marker), HuC/D (pan-neuronal marker), and glial fibrillary acidic protein (GFAP; glial cell marker), as validated by Western blot and immunofluorescence (Figure 1, D and E, and Supplemental Figure 1, A–D; supplemental material available online with this article; https://doi.org/10.1172/JCI192437DS1). At the neurochemical level, protein expression of neuronal nitric oxide synthase (nNOS; a marker of inhibitory nitrergic neurons) was significantly reduced, whereas choline acetyltransferase (ChAT; a marker of cholinergic neurons) remained unchanged (Figure 1, D and E, and Supplemental Figure 1, C and D).

**Figure 2:**
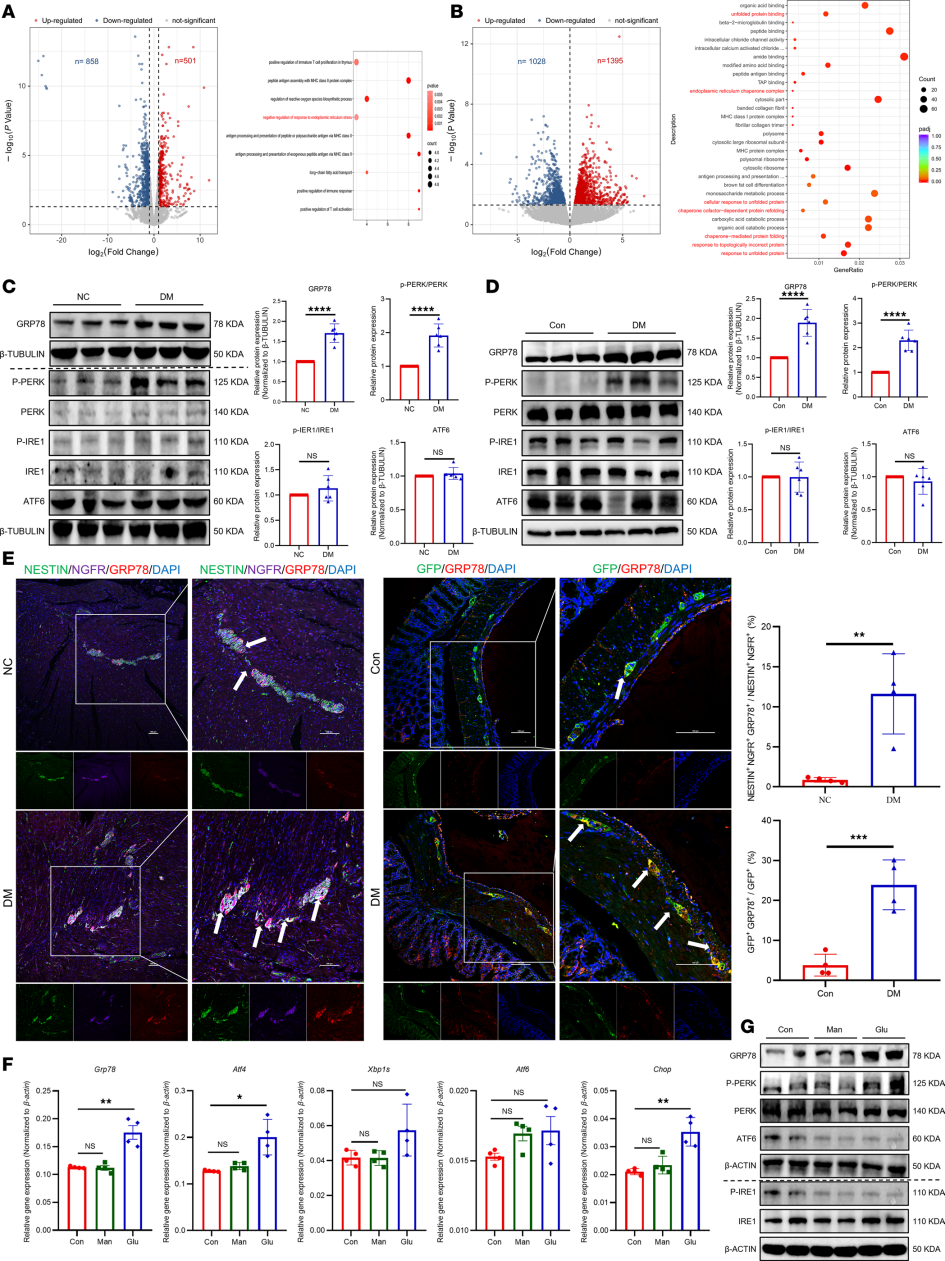
ER stress and UPR were activated in ENPCs under diabetic conditions. (**A** and **B**) Volcano plot and Gene Ontology enrichment of transcriptomic data from human (**A**) and mouse (**B**) colonic samples. *n* = 5 human; *n* = 3 mouse. (**C** and **D**) Western blot and quantification of ER stress/UPR markers (GRP78, p-PERK, PERK, p-IRE1α, IRE1α, and ATF6) in human (**C**) and mouse (**D**) colon samples. *n* = 6. (**E**) Representative immunofluorescence images and quantification of GRP78 in ENPCs from human (left) and mouse (right) colon samples. Scale bars: 100 μm. *n* = 4. (**F**) Relative mRNA expression of ER stress/UPR marker–related genes (*Grp78*, *Atf4*, *Xbp1s*, *Atf6*, and *Chop*) in ENPCs treated with high glucose or mannitol control. *n* = 4. (**G**) Western blot analysis of ER stress/UPR markers in ENPCs treated with high glucose or mannitol as osmotic control. *n* = 4. Data are presented as mean ± SD. Statistical significance was determined by unpaired 2-tailed Student’s *t* test. *P < 0.05, **P < 0.01, ***P < 0.001, ****P < 0.0001; ns, *P* > 0.05.

Interestingly, despite this overall neuronal loss, we observed increased ENPC-derived neurogenesis and gliogenesis in both diabetic patients and mice (Figure 1, F and G, and Supplemental Figure 1E). This dissociation between enhanced regenerative activity and persistent structural degeneration led us to hypothesize that the diabetic microenvironment might compromise the functional efficacy of ENPC-mediated repair. To explore the underlying mechanisms, we performed transcriptomic sequencing on colonic tissues from humans and mice, which revealed substantial enrichment of differentially expressed genes in ER stress and unfolded protein response (UPR) pathways (Figure 2, A and B). Subsequent protein-level analyses confirmed the activation of the ER stress marker 78 kDa glucose-regulated protein (GRP78) and the protein kinase RNA–like ER kinase (PERK) branch of the UPR under diabetic conditions, while the inositol-requiring enzyme 1 alpha (IRE1α) and activating transcription factor 6 (ATF6) branches remained unchanged (Figure 2, C and D). Additionally, fluorescence colocalization analysis revealed elevated GRP78 levels in ENPCs under diabetic conditions (Figure 2E). In vitro, high glucose treatment of primary ENPCs (Supplemental Figure 2, A and B) recapitulated the in vivo findings, which was evidenced by the activation of ER stress and the PERK branch of the UPR (Figure 2, F and G, and Supplemental Figure 2C).

In summary, these results suggest that diabetes-related gastrointestinal dysfunction and ENS damage may be linked to an impaired reparative capacity of ENPCs, in which ER stress and selective UPR activation represent a plausible mechanistic contributor.

### Inhibition of ER stress ameliorates diabetes-induced ENS injury by restoring ENPC function.

**Figure 3:**
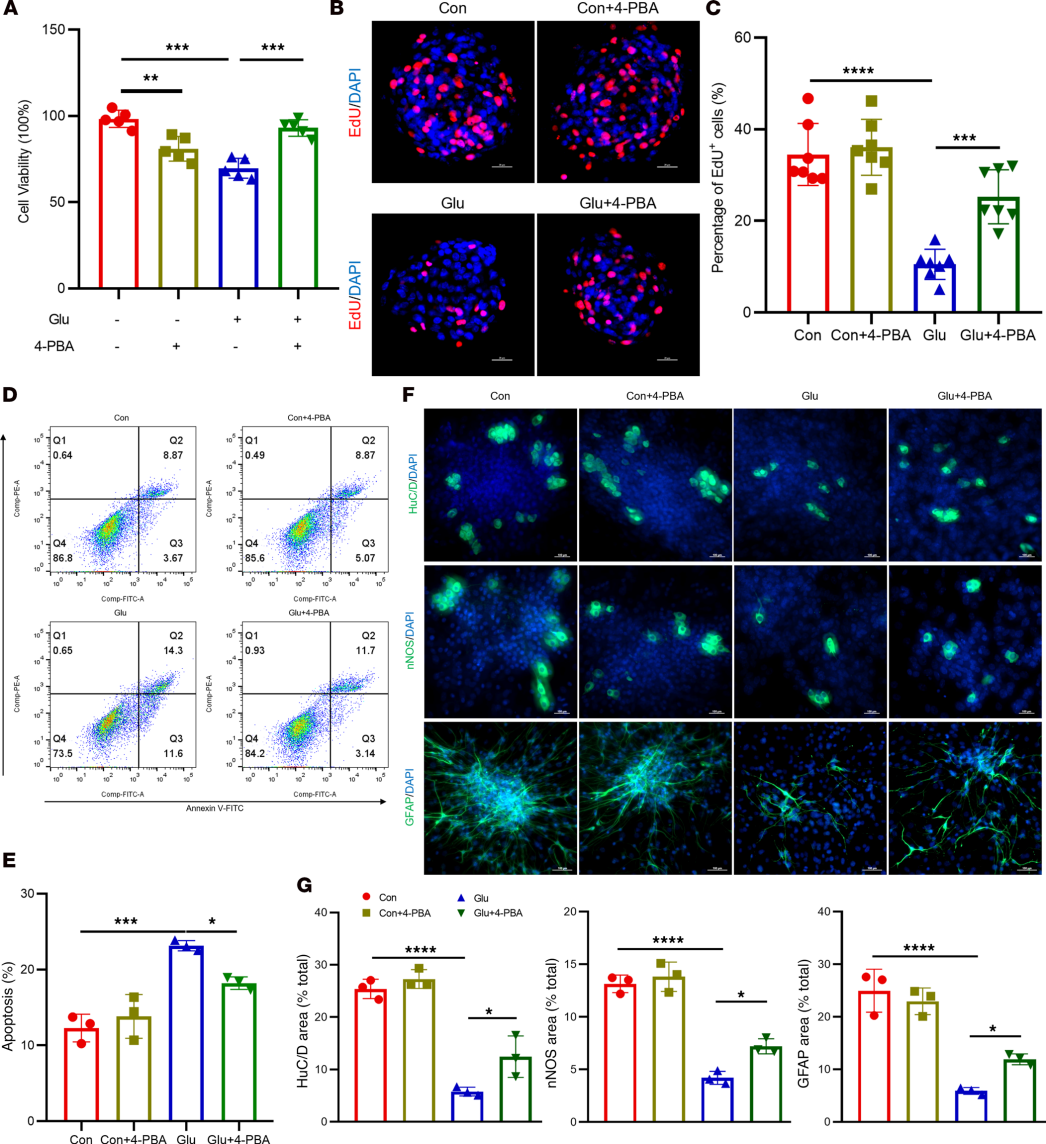
Inhibition of ER stress ameliorates high glucose–induced dysfunction in ENPCs. (**A**) CCK-8 assay assessing the viability of ENPCs following 4-PBA treatment. *n* = 5. (**B** and **C**) Representative immunofluorescence images (**B**) and quantification (**C**) of EdU incorporation in ENPCs treated with 4-PBA. Scale bars: 20 μm. *n* = 7. (**D** and **E**) Representative dot plots of apoptosis (**D**) in ENPCs treated with 4-PBA by annexin V/PI flow cytometry, showing viable (annexin V^–^/PI^–^), early apoptotic (annexin V^+^/PI^–^), and late apoptotic (annexin V^+^/PI^+^) populations, and quantification (**E**) of total apoptotic rate (sum of early and late apoptotic cells). *n* = 3. (**F** and **G**) Representative immunofluorescence images (**F**) and quantification (**G**) of neuronal (HuC/D^+^ and nNOS^+^) and glial (GFAP^+^) differentiation in ENPCs treated with 4-PBA. Scale bars: 100 μm. *n* = 3. Data are presented as mean ± SD. Statistical significance was determined by 1-way ANOVA with Tukey’s multiple-comparison test. *P < 0.05, **P < 0.01, ***P < 0.001, ****P < 0.0001.

Having established the activation of ER stress and the UPR in ENPCs under diabetic conditions, we next asked whether this pathway directly contributes to ENPCs injury. As shown in Figure 3, high glucose treatment significantly suppressed ENPC proliferation (Figure 3, A–C), increased apoptosis (Figure 3, D and E), and impaired neuronal and glial differentiation (Figure 3, F and G). Although chronic ER stress and persistent UPR activation have been implicated in neurodegenerative diseases, their role in diabetic ENS injury remains poorly understood (23). To functionally assess ER stress in this context, we treated ENPCs with the chemical chaperone 4-phenylbutyric acid (4-PBA), a known ER stress inhibitor. Western blot and RT-qPCR confirmed that 4-PBA effectively suppressed high glucose–induced ER stress and the pro-apoptotic PERK branch (Supplemental Figure 3, A and B). Importantly, 4-PBA treatment rescued high glucose–induced deficits in ENPC proliferation (Figure 3, A–C) and differentiation (Figure 3, F and G), while reducing apoptosis (Figure 3, D and E), indicating that ER stress is an important contributor to ENPC dysfunction.

**Figure 4:**
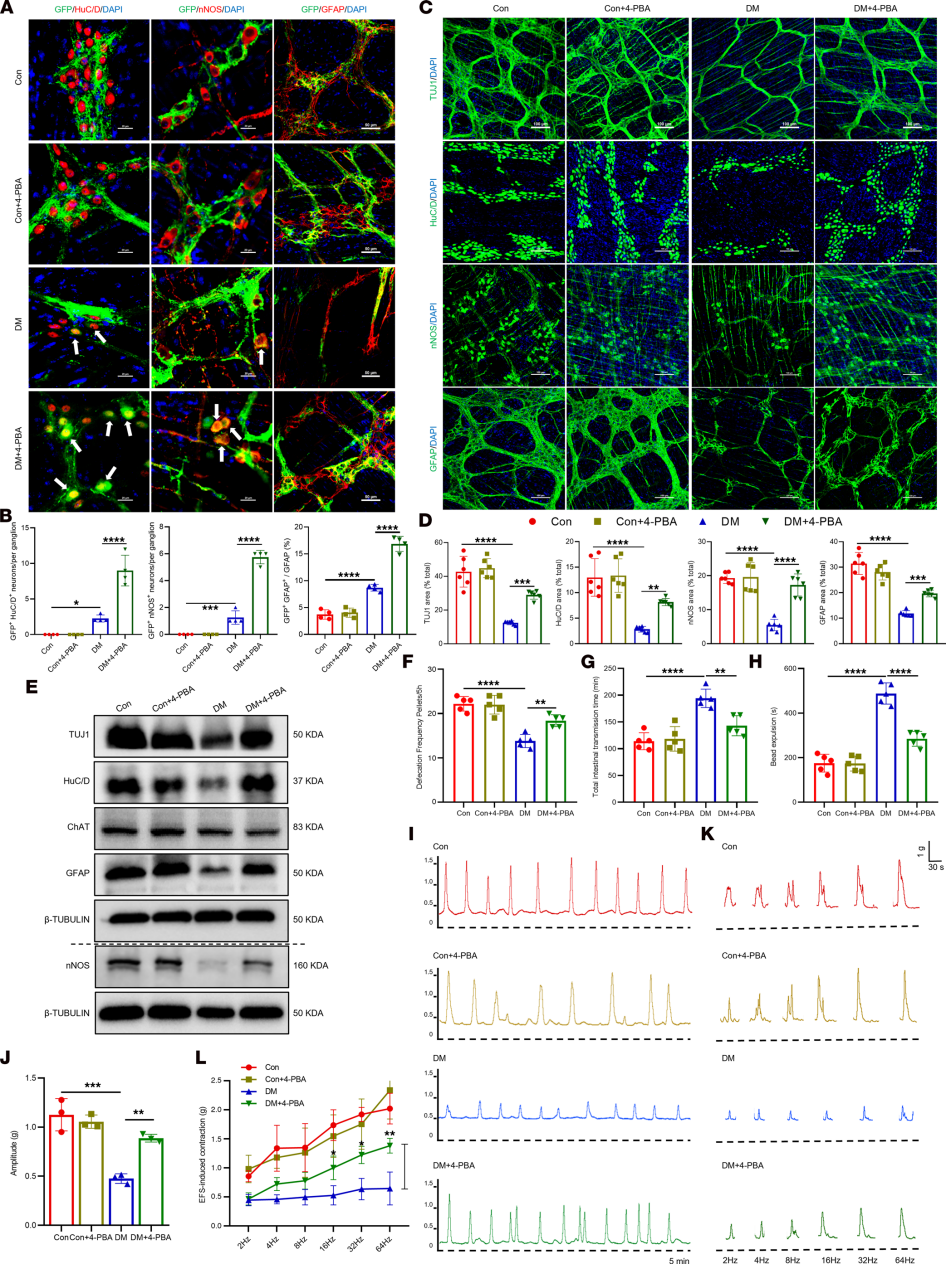
Inhibition of ER stress ameliorates diabetes-induced ENS injury by restoring ENPC function. (**A** and **B**) Representative immunofluorescence images (**A**) and quantification (**B**) of ENPC-derived neurons (HuC/D^+^ and nNOS^+^) and glia (GFAP^+^) in colonic LMMP from 4-PBA–treated mice. Scale bars: 20 μm (left and middle), 50 μm (right). *n* = 4. (**C** and **D**) Representative immunofluorescence images (**C**) and quantification (**D**) of TUJ1, HuC/D, nNOS, and GFAP in colonic LMMP from 4-PBA–treated mice. Scale bars: 100 μm. *n* = 6. (**E**) Western blot analysis of TUJ1, HuC/D, nNOS, ChAT, and GFAP in colon tissues from 4-PBA–treated mice. *n* = 3. (**F**–**H**) Assessment of gastrointestinal motility in 4-PBA–treated mice: defecation frequency (**F**), total intestinal transit time (**G**), and distal colonic transit time (**H**). *n* = 5. (**I** and **J**) Representative images (**I**) and quantitative analysis (**J**) of spontaneous colonic muscle strip contractility in 4-PBA–treated mice (5 minutes). *n* = 3. (**K** and **L**) Representative contractile curve (**K**) and quantification (**L**) of colonic muscle strip induced by EFS at 2, 4, 8, 16, 32, and 64 Hz in 4-PBA–treated mice. *n* = 3. Data are presented as mean ± SD. Statistical significance was determined by 1-way ANOVA with Tukey’s multiple-comparison test. *P < 0.05, **P < 0.01, ***P < 0.001, ****P < 0.0001.

We further evaluated the therapeutic potential of 4-PBA in diabetic mice (Supplemental Figure 3, C and D). Immunofluorescence analyses indicated that 4-PBA significantly promoted the differentiation of ENPCs into enteric neurons (HuC/D^+^GFP^+^ cells), nitrergic neurons (nNOS^+^GFP^+^ cells), and glial cells (GFAP^+^GFP^+^ cells) (Figure 4, A and B). This was accompanied by a marked increase in the number of enteric neurons and glial cells in diabetic mice, nearly restoring counts to control levels (Figure 4, C–E, and Supplemental Figure 3E). Functionally, 4-PBA ameliorated diabetes-related motility impairments, as evidenced by increased defecation frequency (Figure 4F), shortened total gastrointestinal and distal colonic transit times (Figure 4, G and H), and enhanced spontaneous (Figure 4, I and J) and electrically (Figure 4, K and L) evoked contractions of colonic muscle strips. Taken together, these results indicate that ER stress and UPR activation markedly impede ENPC differentiation, thereby compromising ENS repair.

However, CCK-8 assays revealed that 4-PBA monotherapy reduced ENPC viability (Figure 3A), suggesting that systemic ER stress inhibition may have off-target effects that limit its therapeutic utility. Given this limitation, we turned to BMSC-MVs, which have shown the ability to mitigate ER stress with favorable biosafety profiles, representing a promising alternative for regenerative therapy.

### BMSC-MVs alleviate high glucose–induced ENPC damage by suppressing ER stress and the pro-apoptotic PERK branch.

**Figure 5:**
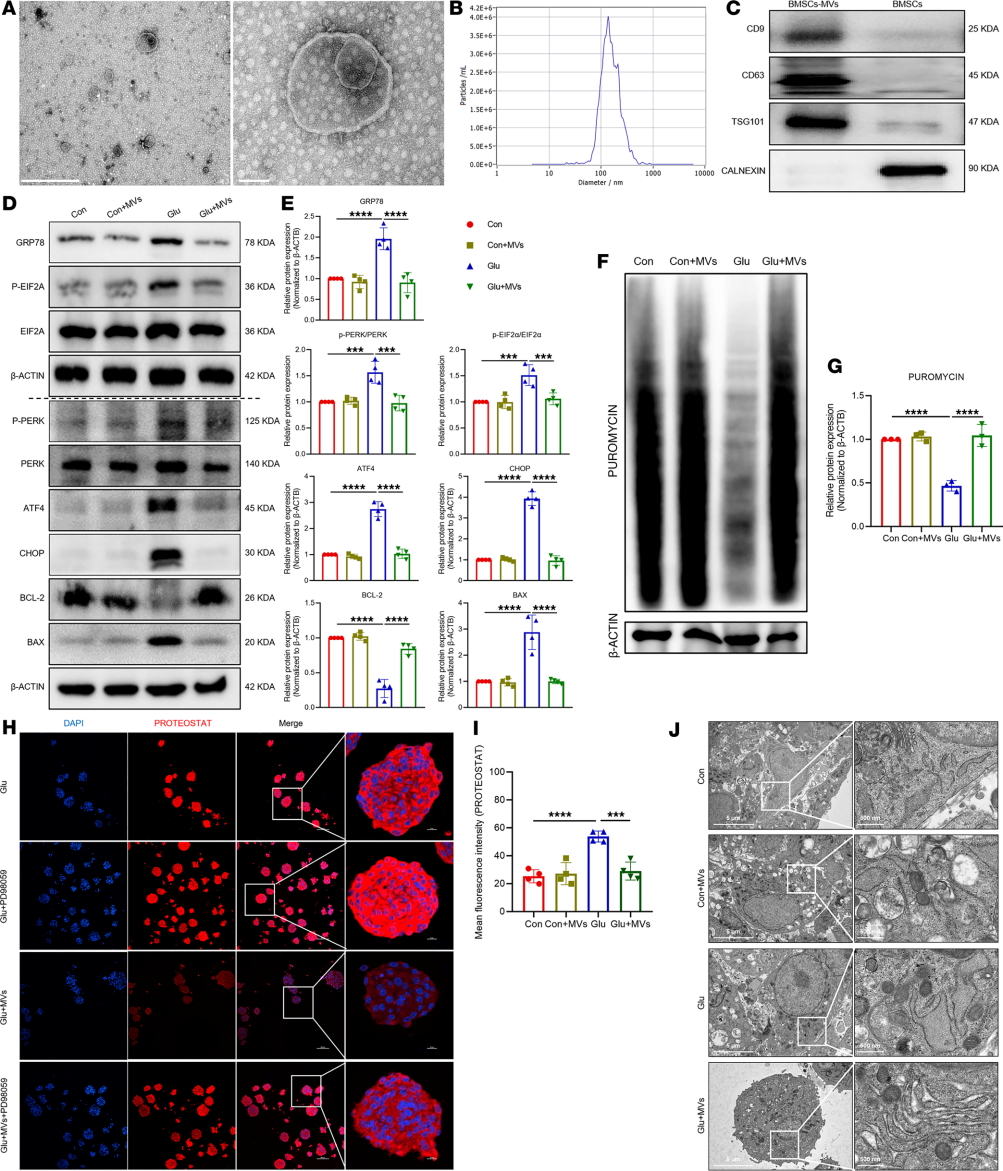
BMSC-MVs alleviate high glucose–induced ENPC damage. (**A**–**C**) Characterization of BMSC-MVs by TEM (**A**), nanoparticle tracking analysis (**B**), and Western blot (**C**) analysis. Scale bars: 1 μm (left), 100 nm (right). (**D** and **E**) Western blot (**D**) and quantification (**E**) of GRP78, p-PERK, PERK, p-EIF2A, EIF2A, ATF4, CHOP, BCL-2, and BAX in ENPCs treated with BMSC-MVs. *n* = 4. (**F** and **G**) Western blot (**F**) and quantification (**G**) of puromycin incorporation assessing global protein synthesis rates in ENPCs treated with BMSC-MVs. *n* = 3. (**H** and **I**) Representative immunofluorescence images (**H**) and quantification (**I**) of PROTEOSTAT-detected protein aggregation in ENPCs treated with BMSC-MVs. Scale bars: 100 μm, 10 μm (enlarged insets). *n* = 4. (**J**) TEM images of ER morphology in ENPCs treated with BMSC-MVs. Scale bars: 5 μm (left), 500 nm (right). Data are presented as mean ± SD. Statistical significance was determined by 1-way ANOVA with Tukey’s multiple-comparison test. ***P < 0.001, ****P < 0.0001.

**Figure 6:**
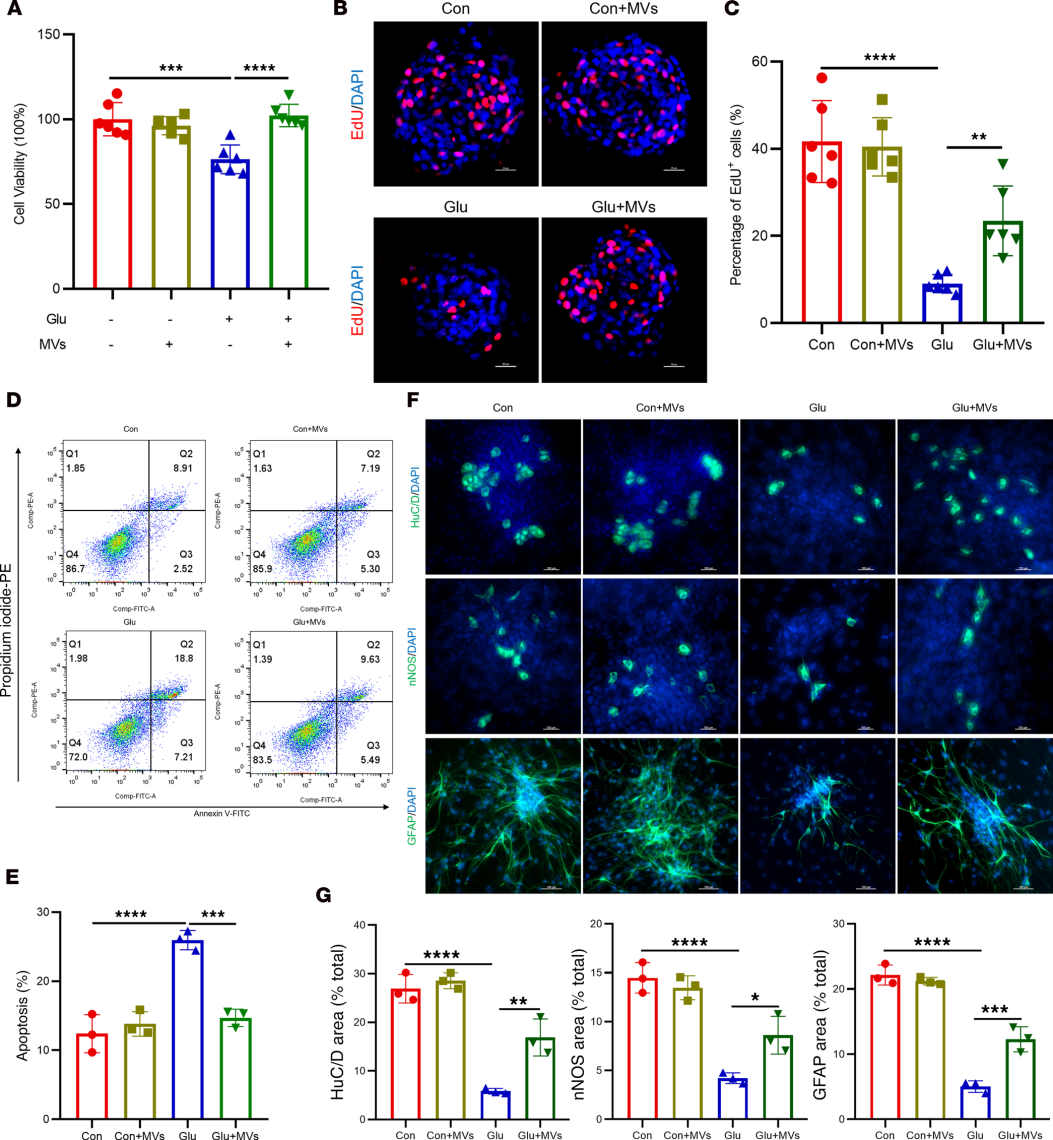
BMSC-MVs suppress ER stress and the pro-apoptotic PERK branch activation in high glucose–treated ENPCs. (**A**) CCK-8 assay assessing the viability of ENPCs treated with BMSC-MVs. *n* = 6. (**B** and **C**) Representative immunofluorescence images (**B**) and quantification (**C**) of EdU incorporation in ENPCs treated with BMSC-MVs. Scale bars: 20 μm. *n* = 6. (**D** and **E**) Representative dot plots of apoptosis (**D**) in ENPCs treated with BMSC-MVs by annexin V/PI flow cytometry, showing viable (annexin V^–^/PI^–^), early apoptotic (annexin V^+^/PI^–^), and late apoptotic (annexin V^+^/PI^+^) populations, and quantification (**E**) of total apoptotic rate (sum of early and late apoptotic cells). *n* = 3. (**F** and **G**) Representative immunofluorescence images (**F**) and quantification (**G**) of HuC/D^+^ neuronal differentiation, nNOS^+^ neuronal differentiation, or GFAP^+^ glial differentiation in ENPCs treated with BMSC-MVs. *n* = 3. Data are presented as mean ± SD. Statistical significance was determined by 1-way ANOVA with Tukey’s multiple-comparison test. *P < 0.05, **P < 0.01, ***P < 0.001, ****P < 0.0001.

We next asked whether BMSC-MVs could mitigate ER stress and restore ENPC function under high-glucose conditions. BMSC-MVs were successfully isolated from BMSC culture supernatant and characterized using transmission electron microscopy (TEM), nanoparticle tracking analysis, and Western blot (Figure 5, A–C). As expected, BMSC-MVs markedly suppressed high glucose–induced ER stress and PERK branch activation in ENPCs, as evidenced by decreased protein levels of GRP78 and the PERK branch (Figure 5, D and E), restored global protein synthesis (Figure 5, F and G), and decreased protein aggregation (Figure 5, H and I). TEM further confirmed that BMSC-MVs restored normal ER morphology, which was notably swollen in high glucose–treated ENPCs (Figure 5J). Functionally, BMSC-MVs markedly alleviated high glucose–induced ENPC damage, enhancing proliferation (Figure 6, A–C), reducing apoptosis (Figure 6, D and E), and improving differentiation capacity (Figure 6, F and G). Notably, unlike 4-PBA, BMSC-MVs did not impair ENPC viability under physiological conditions. Taken together, these results indicate that BMSC-MVs effectively ameliorate high glucose–induced ENPC damage, an effect closely associated with the suppression of ER stress and the pro-apoptotic PERK pathway.

### Surface vinculin on BMSC-MVs mediates internalization by binding to TLN1 on ENPCs.

**Figure 7:**
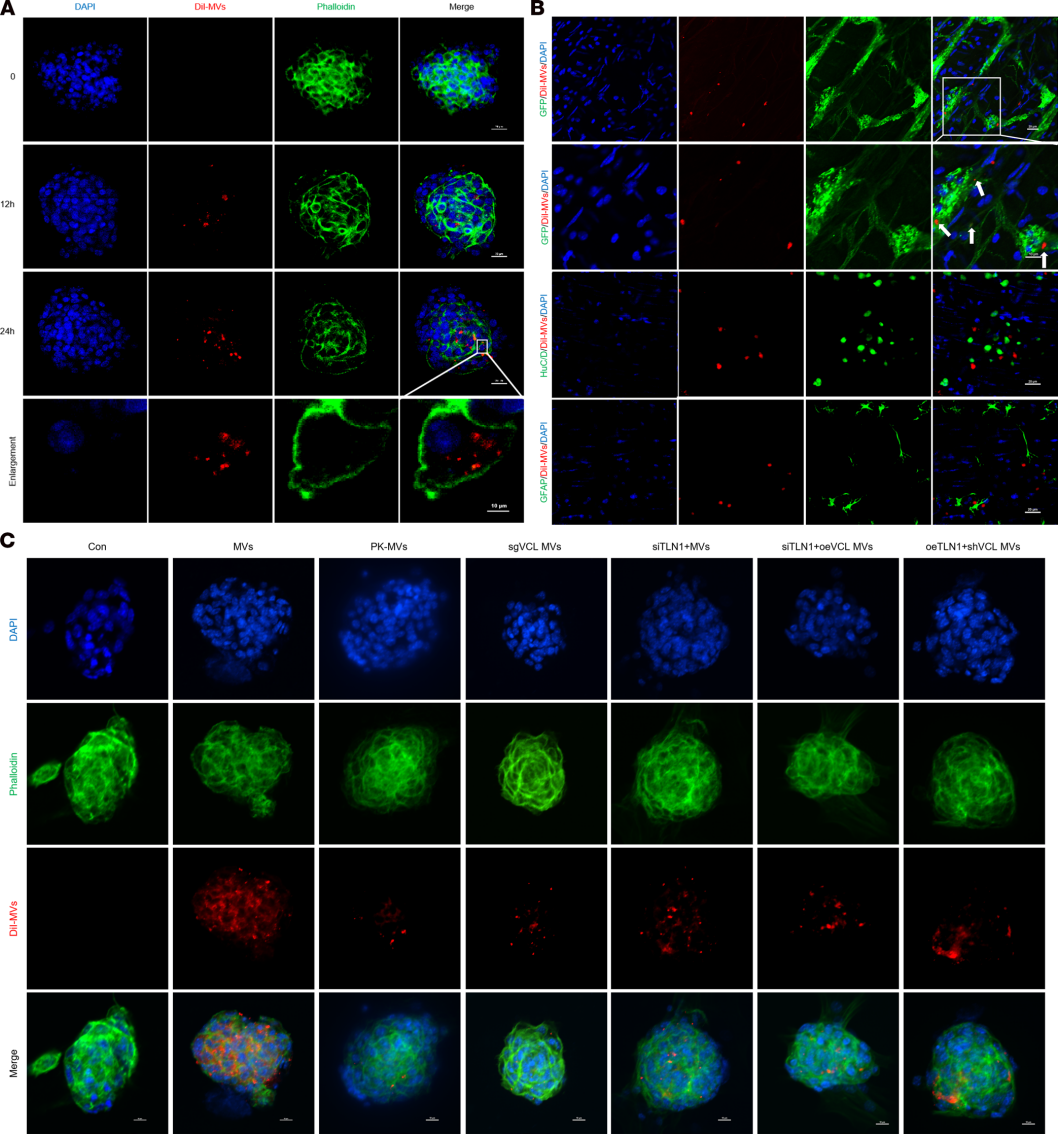
Uptake of BMSC-MVs by ENPCs and distribution within the ENS. (**A**) Uptake of DiI-labeled BMSC-MVs (red) by FITC-phalloidin–stained ENPCs (green) at 0, 12, and 24 hours. Scale bars: 20 μm, 10 μm (enlarged insets). *n* = 3. (**B**) Distribution of DiI-labeled BMSC-MVs in ENPCs, neurons, and glial cells within colonic LMMP 24 hours after incubation. Scale bars: 20 and 10 μm. *n* = 3. (**C**) Representative images showing BMSC-MV uptake by ENPCs across experimental groups. Scale bars: 10 μm. *n* = 3.

To elucidate the mechanism by which BMSC-MVs regulate ER stress in ENPCs, we focused on the internalization process, which is critical for EV-mediated functional delivery and depends on specific ligand–receptor interactions between EV membrane proteins and target cell surface receptors (24). We first confirmed that BMSC-MVs were internalized by ENPCs in a time-dependent manner, as shown by 1,1′-dioctadecyl-3,3,3′,3′-tetramethylindocarbocyanine perchlorate (DiI) fluorescent labeling (Figure 7A). Ex vivo coculture of longitudinal muscle myenteric plexus (LMMP) preparations with BMSC-MVs revealed that the DiI-labeled BMSC-MVs were predominantly localized in GFP^+^ ENPCs, with minimal uptake by HuC/D^+^ neurons or GFAP^+^ glial cells (Figure 7B), suggesting a receptor-mediated uptake mechanism. This was further supported by a marked reduction in ENPC uptake following proteinase K treatment of BMSC-MVs, which removes surface proteins (Figure 7C).

**Figure 8:**
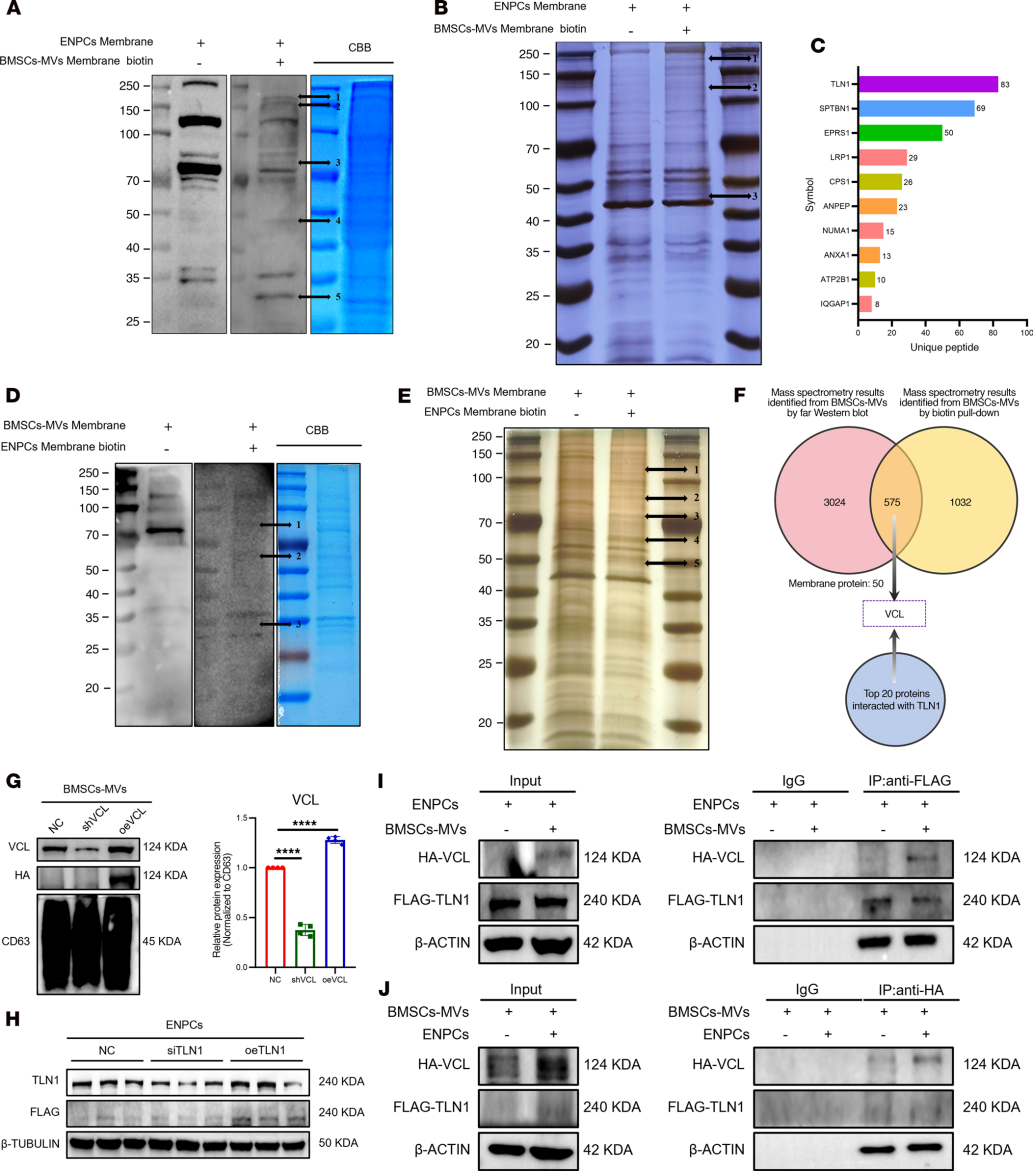
Surface VCL on BMSC-MVs mediates internalization by binding to TLN1 on ENPCs. (**A** and **B**) Screening of ENPC surface proteins interacting with BMSC-MVs by far Western blot (**A**) and biotin pull-down (**B**). Far Western analysis revealed 5 candidate bands, with band 1 identified as TLN1 by LC-MS/MS; biotin pull-down showed 3 bands, with band 1 confirmed as TLN1. CBB, Coomassie blue. (**C**) Peptide-based ranking of ENPC surface proteins interacting with BMSC-MVs from far Western blot. (**D** and **E**) Screening of BMSC-MV surface proteins by far Western blot (**D**) and biotin pull-down (**E**). Far Western showed 3 candidates, with band 1 identified as VCL; biotin pull-down revealed 5 bands, with band 1 identified as VCL by LC-MS/MS. (**F**) Venn diagram of overlapping ENPC surface proteins identified by far Western and biotin pull-down assays. (**G**) Western blot analysis and qualification of VCL in BMSC-MVs after *Vcl* knockdown (shVCL) or overexpression (oeVCL). *n* = 4. (**H**) Western blot analysis of TLN1 in ENPCs after *Tln1* knockdown (siTLN1) or overexpression (oeTLN1). *n* = 3. (**I** and **J**) Coimmunoprecipitation assay to assess the binding of HA-tagged VCL in BMSC-MVs to FLAG-tagged TLN1 in ENPCs. IgG control is shown. Data are presented as mean ± SD. Statistical significance was determined by unpaired 2-tailed Student’s *t* test. ****P < 0.0001.

We next identified the specific interacting membrane proteins from ENPCs using far Western blot (Figure 8A) and biotin pull-down assays (Figure 8B). Liquid chromatography–tandem mass spectrometry (LC-MS/MS) analysis identified talin-1 (TLN1), a membrane-associated protein involved in cell adhesion, as a top candidate based on unique peptide counts (Figure 8C and Supplemental Table 1). Similarly, among the candidate membrane proteins from BMSC-MVs (Figures 8, D and E, Supplemental Figure 4A, and Supplemental Table 2), vinculin (VCL) was selected due to its known high binding affinity for TLN1 (Figure 8F). We then constructed HA-tagged *Vcl-*overexpressing BMSC-MVs (Figures 8G and Supplemental Figure 4B) and FLAG-tagged *Tln1*-overexpressing ENPCs (Figures 8H and Supplemental Figure 4C). Coimmunoprecipitation assays confirmed a direct physical interaction between TLN1 on ENPCs and VCL on BMSC-MVs (Figure 8, I and J).

Functional validation through loss-of-function experiments showed that knockdown of *Vcl* in BMSCs using short hairpin RNA or sgRNA (Supplemental Figure 4, B and D) did not alter MV marker expression (shVCL MVs or sgVCL MVs; Figure 8G and Supplemental Figure 4, E and F) but significantly impaired their uptake by ENPCs (Figure 7C). Similarly, siRNA-mediated knockdown of *Tln1* in ENPCs (Figure 8H and Supplemental Figure 4, C and G) markedly reduced BMSC-MV internalization (Figure 7C). Rescue experiments further confirmed the specificity of this interaction: in *Tln1*-knockdown ENPCs, overexpression of *Vcl* in BMSC-MVs failed to restore internalization. Conversely, in *Tln1*-overexpressing ENPCs, *Vcl*-knockdown BMSC-MVs still showed impaired uptake (Figure 7C). Together, these results demonstrate that the specific VCL–TLN1 interaction is essential for efficient internalization of BMSC-MVs by ENPCs.

### VCL–TLN1 interaction is required for BMSC-MV–mediated functional recovery in ENPCs.

**Figure 9:**
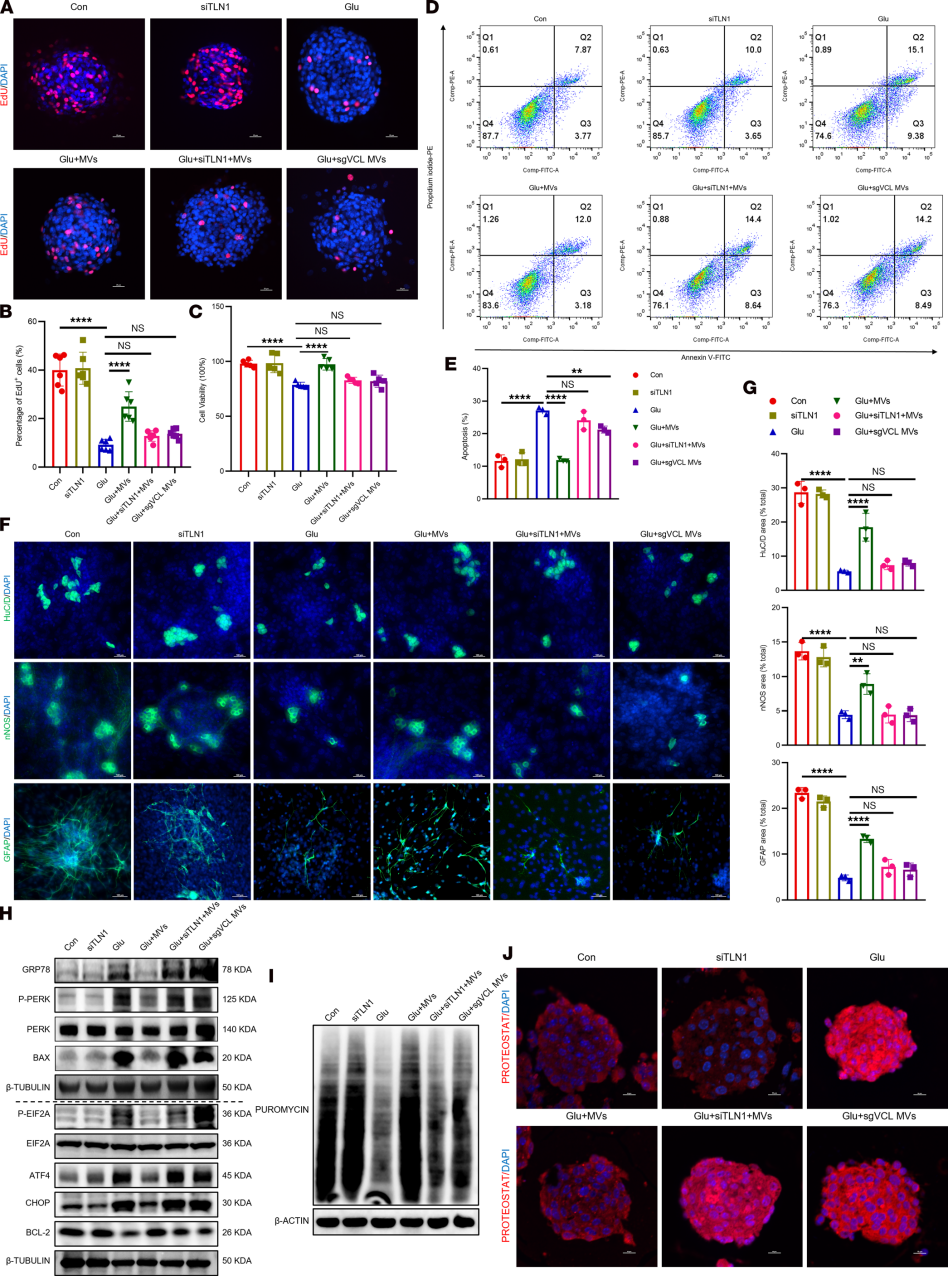
Loss of VCL or TLN1 abolishes the protective effects of BMSC-MVs on ENPCs. (**A** and **B**) Representative immunofluorescence images (**A**) and quantification (**B**) of EdU incorporation in ENPCs. Scale bars: 20 μm. *n* = 6. (**C**) CCK-8 assay assessing ENPC viability under different treatments. *n* = 5. (**D** and **E**) Representative dot plots of apoptosis (**D**) in ENPCs under different treatments by annexin V/PI flow cytometry, showing viable (annexin V^–^/PI^–^), early apoptotic (annexin V^+^/PI^–^), and late apoptotic (annexin V^+^/PI^+^) populations, and quantification (**E**) of total apoptotic rate (sum of early and late apoptotic cells). *n* = 3. (**F** and **G**) Representative immunofluorescence images (**F**) and quantification (**G**) of neuronal (HuC/D^+^ and nNOS^+^) and glial (GFAP^+^) differentiation in ENPCs under different treatments. Scale bars: 100 μm. *n* = 3. (**H**) Western blot analysis of GRP78, p-PERK, PERK, p-EIF2A, EIF2A, ATF4, CHOP, BCL-2, and BAX proteins in ENPCs under different treatments. *n* = 3. (**I**) Western blot analysis of puromycin incorporation assessing global protein synthesis rates in ENPCs under different treatments. *n* = 3. (**J**) Representative immunofluorescence images of PROTEOSTAT-detected protein aggregation in ENPCs under different treatments. Scale bars: 10 μm. *n* = 4. Data are presented as mean ± SD. Statistical significance was determined by 1-way ANOVA with Tukey’s multiple-comparison test. **P < 0.01, ****P < 0.0001; ns, *P* > 0.05.

Having established that VCL-TLN1 mediates the internalization of BMSC-MVs, we next asked whether this interaction is necessary for their therapeutic function. Disruption of the VCL–TLN1 axis — via knocking down *Vcl* in BMSC-MVs or *Tln1* in ENPCs — abolished the ability of BMSC-MVs to restore proliferation (Figure 9, A–C), inhibit apoptosis (Figure 9, D and E), and promote differentiation (Figure 9, F and G) in high glucose–treated ENPCs. Mechanistically, interfering with VCL-TLN1 binding blocked BMSC-MV–mediated suppression of ER stress and UPR activation, as reflected by increased protein levels of GRP78 and PERK branch components (Figure 9H and Supplemental Figure 5A), decreased global protein synthesis (Figure 9I and Supplemental Figure 5B), and elevated protein aggregation (Figure 9J and Supplemental Figure 5C).

**Figure 10:**
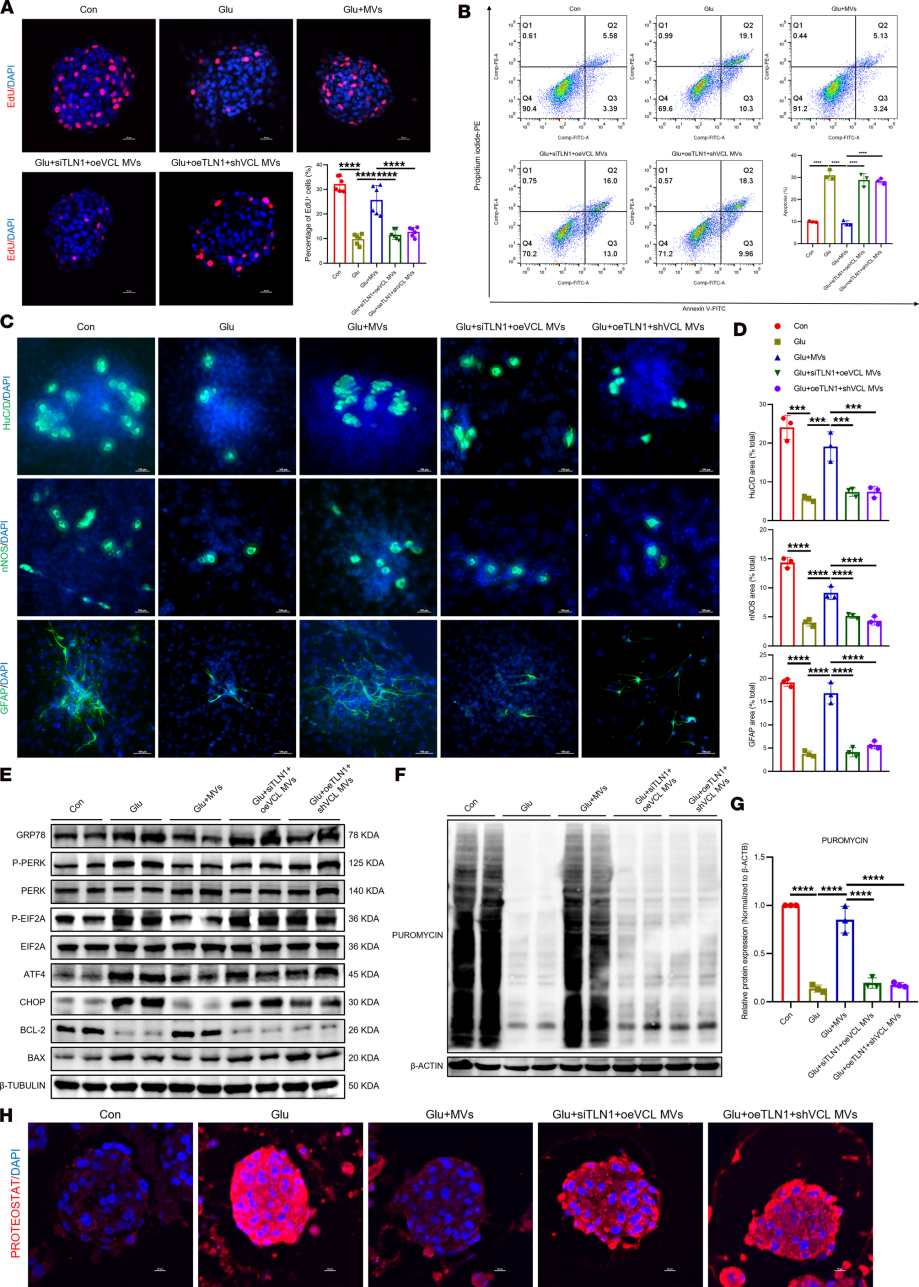
VCL–TLN1 interaction is required for BMSC-MV–mediated functional recovery in ENPCs. (**A**) Representative immunofluorescence images and quantification of EdU incorporation in ENPCs in rescue experiments. Scale bars: 20 μm. *n* = 6. (**B**) Flow cytometric analysis of apoptosis using annexin V/PI staining in ENPCs in rescue experiments. *n* = 3. (**C** and **D**) Representative immunofluorescence images (**C**) and quantification (**D**) of neuronal (HuC/D^+^ and nNOS^+^) and glial (GFAP^+^) differentiation in ENPCs in rescue experiments. Scale bars: 100 μm. *n* = 3. (**E**) Western blot analysis of GRP78, p-PERK, PERK, p-EIF2A, EIF2A, ATF4, CHOP, BCL-2, and BAX proteins in ENPCs in rescue experiments. *n* = 3. (**F** and **G**) Western blot analysis (**F**) and quantification (**G**) of puromycin incorporation assessing global protein synthesis rates in ENPCs in rescue experiments. *n* = 3. (**H**) Representative immunofluorescence images of PROTEOSTAT-detected protein aggregation in ENPCs in rescue experiments. Scale bars: 10 μm. *n* = 4. Data are presented as mean ± SD. Statistical significance was determined by 1-way ANOVA with Tukey’s multiple-comparison test. ***P < 0.001, ****P < 0.0001.

Rescue experiments further confirmed the strict dependence of functional recovery on VCL-TLN1 engagement. In *Tln1*-knockdown ENPCs, *Vcl*-overexpressing BMSC-MVs failed to restore proliferation (Figure 10A and Supplemental Figure 5D), apoptosis resistance (Figure 10B), differentiation (Figure 10, C and D), or ER stress homeostasis (Figure 10, E–H, and Supplemental Figure 5, E and F). Similarly, in *Tln1*-overexpressing ENPCs, *Vcl*-knockdown BMSC-MVs remained functionally impaired (Figure 10 and Supplemental Figure 5, D–F). These results indicate that the therapeutic benefits of BMSC-MVs depend not only on cellular entry but also on downstream signaling events specifically triggered by the VCL–TLN1 interaction. Together, our findings identify the VCL–TLN1 interaction as a key regulator that coordinates the internalization and reparative function of BMSCs-MVs in high glucose–induced ENPC injury.

### BMSC-MVs alleviate high glucose–induced ENPCs injury via ERK-dependent suppression of ER stress.

**Figure 11:**
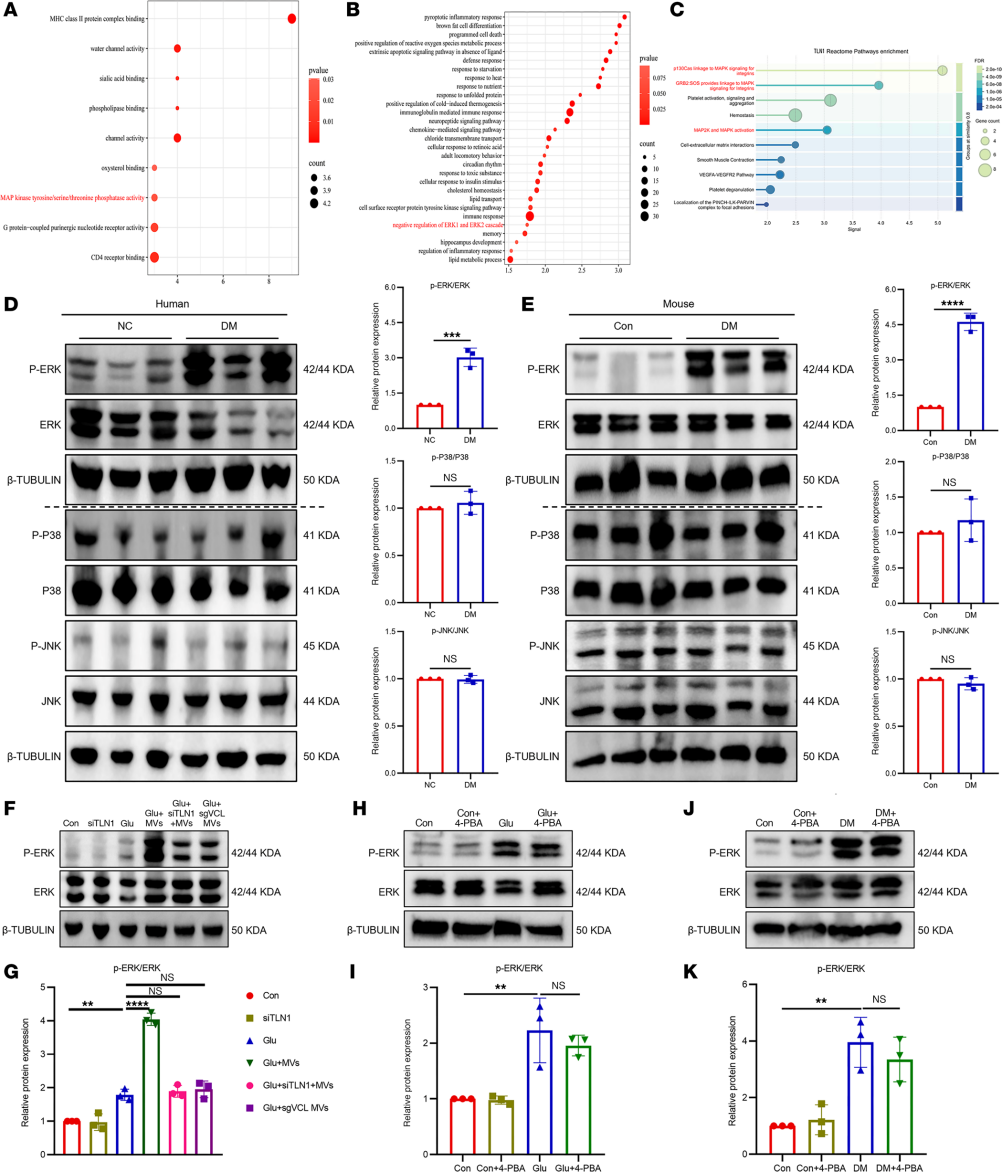
ERK activation is identified as a key event in BMSC-MV–mediated protection. (**A** and **B**) KEGG enrichment analysis of differentially expressed genes from transcriptomic sequencing of colonic tissues of human (**A**) and mouse (**B**) samples. (**C**) Reactome pathway enrichment analysis of TLN1 using the STRING online website (https://www.string-db.org/). (**D** and **E**) Western blot and quantification of p-ERK, ERK, p-P38, P38, p-JNK, and JNK in the colonic tissues of human (**D**) and mouse (**E**) samples. *n* = 3. (**F** and **G**) Western blot (**F**) and quantification (**G**) of p-ERK and ERK in ENPCs under different treatments. *n* = 3. (**H**–**K**) Western blot and quantification of p-ERK and ERK in ENPCs (**H** and **I**) or mice (**J** and **K**) treated with 4-PBA. *n* = 3. Data are presented as mean ± SD. Statistical significance was determined by unpaired 2-tailed Student’s *t* test (**D** and **E**) and 1-way ANOVA with Tukey’s multiple-comparison test (**G**, **I**, and **K**). **P < 0.01, ***P < 0.001, ****P < 0.0001; ns, *P* > 0.05.

To elucidate the link between VCL-TLN1 engagement and ER stress suppression, we performed Kyoto Encyclopedia of Genes and Genomes (KEGG) pathway analysis on transcriptomic data from human and murine samples, which revealed significant enrichment of differentially expressed genes in the MAPK cascade (Figure 11, A and B). In addition, multiple TLN1-related pathways were associated with MAPK activation (Figure 11C). Given the established role of the MAPK cascade — comprising JNK, ERKs, and P38 cascades — in stem cell differentiation (25, 26), we evaluated the activation status of these branches. Under diabetic conditions, only the ERK pathway was significantly upregulated, whereas JNK and P38 remained unaffected (Figure 11, D and E), prompting us to focus on ERK in subsequent experiments.

We found that BMSC-MVs enhanced ERK pathway activation in ENPCs under high glucose, an effect that was abolished upon knockdown of *Vcl* in BMSC-MVs or *Tln1* in ENPCs (Figure 11, F and G), indicating that VCL-TLN1 binding is required for ERK activation. In contrast, treatment with the ER stress inhibitor 4-PBA did not influence ERK activation in high glucose–treated ENPCs (Figure 11, H and I) or in diabetic mice (Figure 11, J and K), suggesting that ERK acts upstream of ER stress and is regulated by VCL–TLN1 signaling.

**Figure 12:**
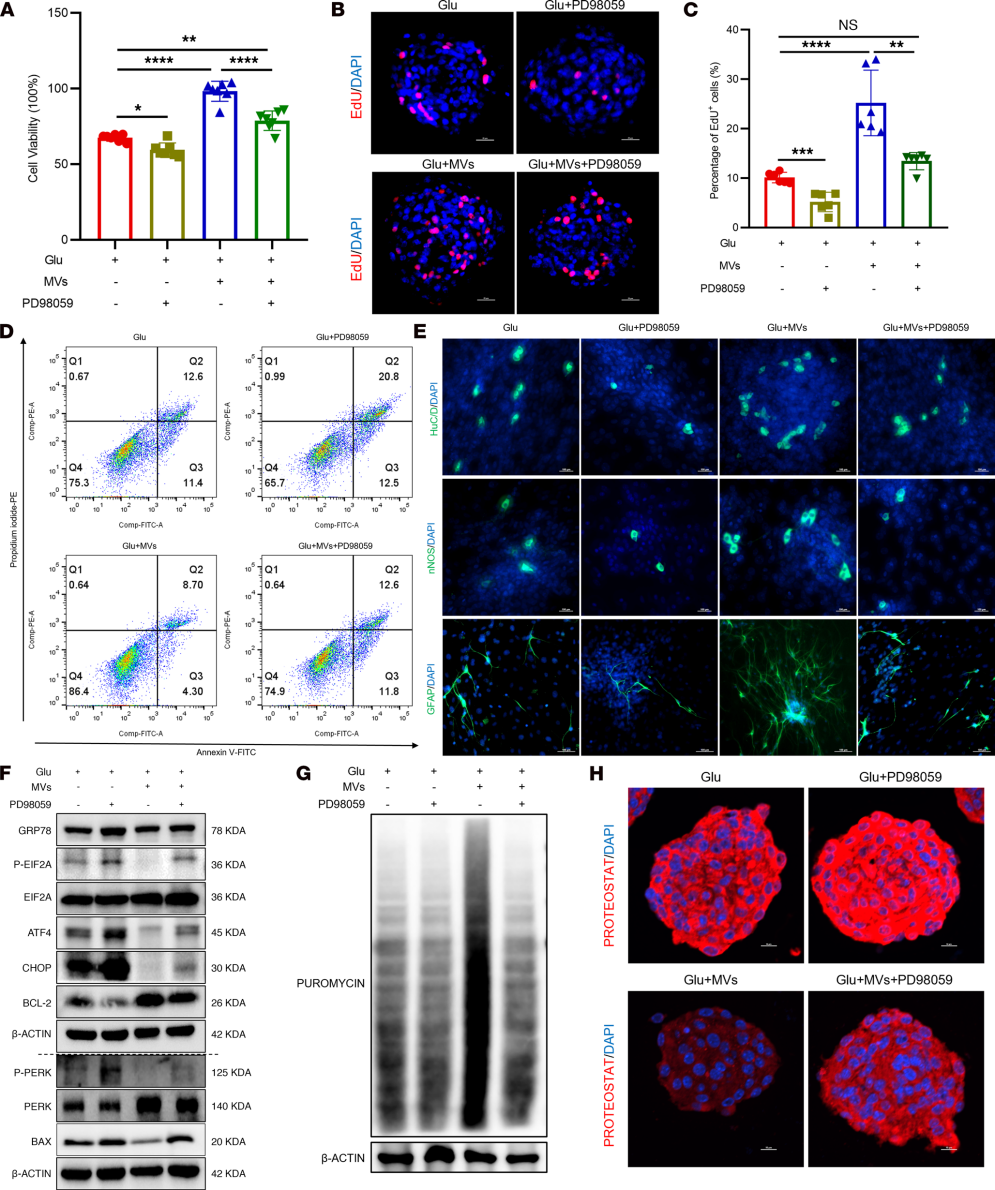
Pharmacological inhibition of ERK abolishes the protective effects of BMSC-MVs on ENPCs. (**A**) CCK-8 assay assessing the viability of ENPCs treated with ERK inhibitor PD98059. *n* = 7. (**B** and **C**) Representative immunofluorescence images (**B**) and quantification (**C**) of EdU incorporation in ENPCs treated with ERK inhibitor PD98059. Scale bars: 20 μm. *n* = 6. (**D**) Flow cytometric analysis of apoptosis using annexin V/PI staining in ENPCs treated with ERK inhibitor PD98059. *n* = 3. (**E**) Representative immunofluorescence images of neuronal (HuC/D^+^ and nNOS^+^) and glial (GFAP^+^) differentiation in ENPCs treated with ERK inhibitor PD98059. Scale bars: 100 μm. *n* = 3. (**F**) Western blot analysis of GRP78, p-PERK, PERK, p-EIF2A, EIF2A, ATF4, CHOP, BCL-2, and BAX proteins in ENPCs treated with ERK inhibitor PD98059. *n* = 3. (**G**) Western blot analysis of puromycin incorporation assessing global protein synthesis rates in ENPCs treated with ERK inhibitor PD98059. *n* = 3. (**H**) Representative immunofluorescence images of PROTEOSTAT-detected protein aggregation in ENPCs treated with ERK inhibitor PD98059. Scale bars: 10 μm. *n* = 4. Data are presented as mean ± SD. Statistical significance was determined by 1-way ANOVA with Tukey’s multiple-comparison test. *P < 0.05, **P < 0.01, ***P < 0.001, ****P < 0.0001; ns, *P* > 0.05.

We next inhibited ERK in primary ENPCs using the small-molecule inhibitor PD98059 (Supplemental Figure 6A). ERK suppression exacerbated high glucose–induced impairments in proliferation (Figure 12, A–C), increased apoptosis (Figure 12D and Supplemental Figure 6B), impaired differentiation (Figure 12E and Supplemental Figure 6C), and intensified ER stress (Figure 12, F–H, and Supplemental Figure 6, D–F). Critically, ERK inhibition completely abolished the ability of BMSC-MVs to restore ENPC function and suppress ER stress and the PERK branch under high glucose (Figure 12 and Supplemental Figure 6). Together, these results demonstrate that surface VCL on BMSC-MVs binds to TLN1 on ENPCs to activate the ERK signaling pathway, which in turn attenuates ER stress and UPR activation, thereby preserving ENPC function.

### BMSC-MVs ameliorate diabetes-induced ENS injury via ER stress and PERK branch suppression in ENPCs.

**Figure 13:**
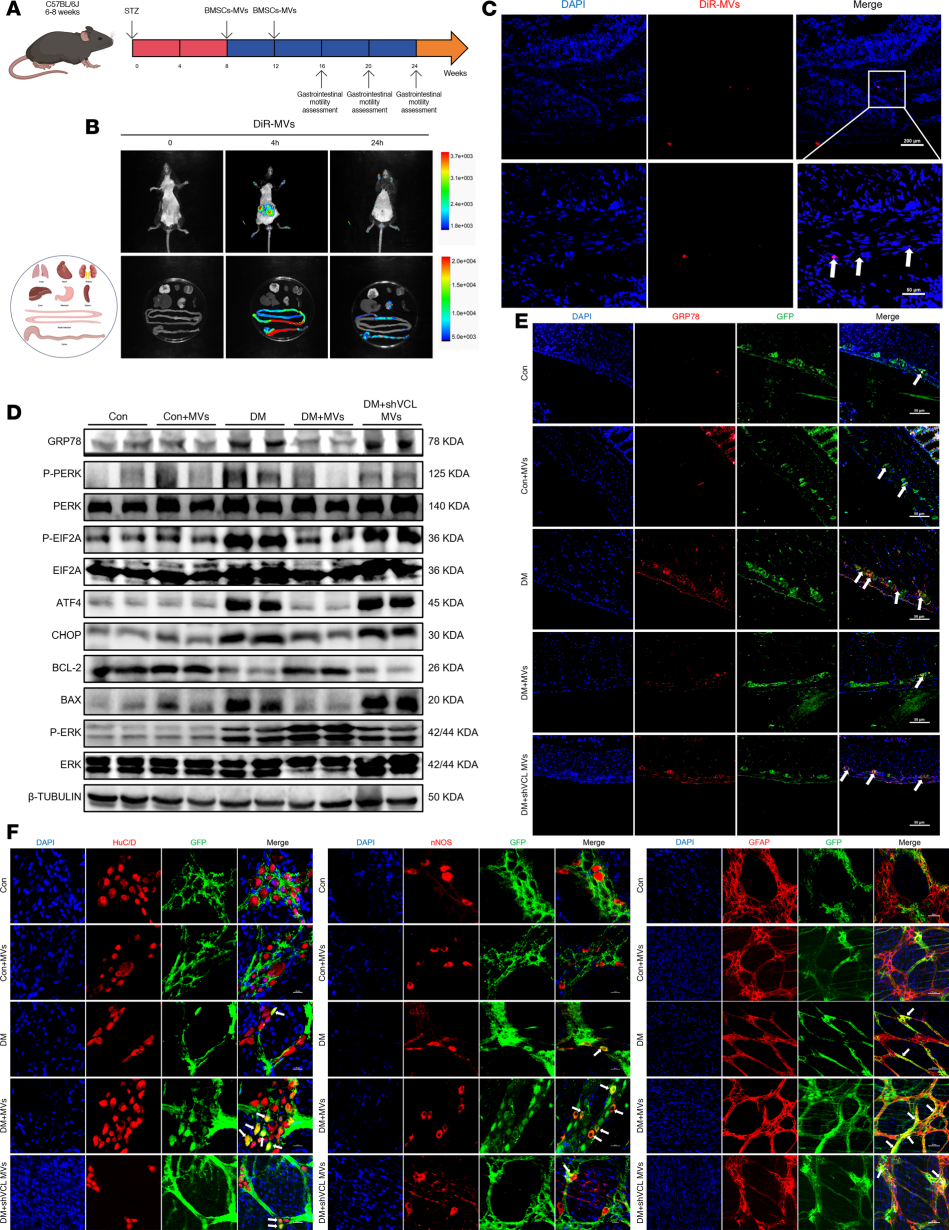
BMSC-MVs suppress ER stress and promote ENPC differentiation in ENPCs in diabetic mice. (**A**) Experimental timeline of BMSC-MV treatment and gastrointestinal motility assessment. (**B**) In vivo distribution of BMSC-MVs at 0, 4, and 24 hours after injection. (**C**) Representative immunofluorescence images of DiR-labeled BMSC-MVs in colon at 24 hours. Scale bars: 200 μm (top), 50 μm (bottom). (**D**) Western blot analysis of GRP78, p-PERK, PERK, p-EIF2A, EIF2A, ATF4, CHOP, BCL-2, BAX, and p-ERK in colon tissues from BMSC-MV–treated mice. *n* = 3. (**E**) Representative immunofluorescence images of GRP78 in ENPCs in colon from BMSC-MV–treated mice. Scale bars: 50 μm. (**F**) Representative immunofluorescence images and quantification of ENPC-derived neurons (HuC/D^+^ and nNOS^+^), and GFAP^+^ glial cells in colonic LMMP from BMSC-MV–treated mice. Scale bars: 20 μm (left and middle), 50 μm (right). *n* = 4.

We next evaluated the therapeutic potential of BMSC-MVs in a diabetic mouse model (Figure 13A). Following intraperitoneal injection of 1,1-dioctadecyl-3,3,3,3-tetramethylindotricarbocyaine iodide–labeled (DiR-labeled) BMSC-MVs, in vivo imaging revealed strong abdominal fluorescence at 4 hours, which persisted but diminished by 24 hours (Figure 13B). Ex vivo imaging confirmed substantial BMSC-MV accumulation in the colon and small intestine (Figure 13B and Supplemental Figure 7A), and frozen sections showed their localization within the muscular layer (Figure 13C). These findings suggest that BMSC-MVs could efficiently reach the colonic muscle layer following intraperitoneal injection, providing valuable evidence for investigating their potential therapeutic effects in the repair of ENS injury.

Consistent with in vitro observation, BMSC-MV treatment significantly downregulated mRNA and protein levels of GRP78, p-PERK, p-EIF2A, ATF4, CHOP, and BAX in the colon of diabetic mice, while increasing BCL-2 and p-ERK expression (Figure 13D and Supplemental Figure 7, B and C). Fluorescence colocalization further confirmed a marked reduction in GRP78 levels within ENPCs after BMSC-MV administration (Figure 13E). Furthermore, BMSC-MVs robustly promoted neurogenesis and gliogenesis, as shown by a substantial increase in the number of HuC/D^+^GFP^+^ neurons, nNOS^+^GFP^+^ neurons, and GFAP^+^GFP^+^ glial cells (Figure 13F and Supplemental Figure 7D).

**Figure 14:**
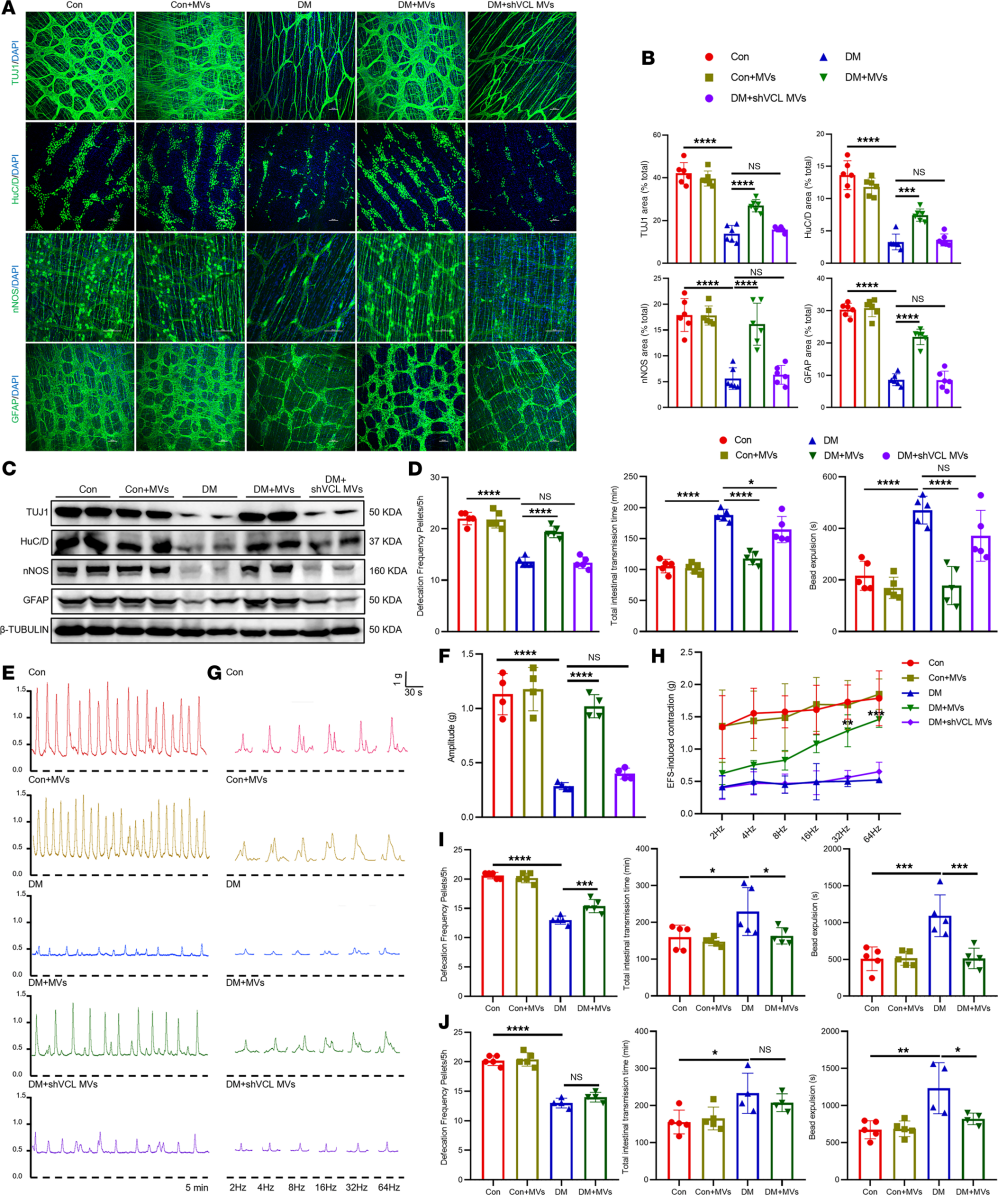
BMSC-MVs restore ENS structure and gastrointestinal motility in diabetic mice. (**A** and **B**) Representative immunofluorescence images (**A**) and quantification (**B**) of TUJ1, HuC/D, nNOS, and GFAP in colonic LMMP from BMSC-MV–treated mice. Scale bars: 100 μm. *n* = 6. (**C**) Western blot analysis of TUJ1, HuC/D, nNOS, and GFAP in colon tissues from BMSC-MV–treated mice. (**D**) Gastrointestinal motility assessment in BMSC-MV–treated mice at 16 weeks after STZ injection. *n* = 4–5. (**E** and **F**) Representative images (**E**) and quantification (**F**) of spontaneous colonic muscle strip contractility from BMSC-MV–treated mice (5 minutes). *n* = 4. (**G** and **H**) Representative contractile curve (**G**) and quantification (**H**) of colonic muscle strip induced by EFS at 2, 4, 8, 16, 32, and 64 Hz from BMSC-MV–treated mice. *n* = 3. (**I** and **J**) Gastrointestinal motility assessment in BMSC-MV–treated mice at 20 (**I**) and 24 (**J**) weeks after STZ injection. *n* = 4–5. Data are presented as mean ± SD. Statistical significance was determined by 1-way ANOVA with Tukey’s multiple-comparison test. *P < 0.05, **P < 0.01, ***P < 0.001, ****P < 0.0001; ns, *P* > 0.05.

BMSC-MVs also restored ENS network integrity and gastrointestinal motility. Immunofluorescence (Figure 14, A and B) and Western blot (Figure 14C and Supplemental Figure 7E) analyses showed recovery of TUJ1^+^ neuronal fibers, HuC/D^+^ neurons, nNOS^+^ neurons, and GFAP^+^ glial cells. Accordingly, administration of BMSC-MVs significantly ameliorated gastrointestinal dysmotility (Figure 14D) and enhanced both spontaneous and electrical field stimulation–induced (EFS-induced) contractility of colonic muscle strips in diabetic mice (Figure 14, E–H). Importantly, all these therapeutic effects were abolished in mice that received *Vcl*-knockdown BMSC-MVs, which failed to suppress ER stress, activate ERK, promote ENPC differentiation, or restore ENS structure and motility, confirming the essential role of VCL in mediating the reparative effects of BMSC-MVs (Figure 13, D–F, and Figure 14, A–H). We further evaluated the durability of the therapeutic response. The protective effects of BMSC-MVs persisted for 8 weeks after the second administration (Figure 14I). By 24 weeks after streptozotocin (STZ) injection (12 weeks after the second BMSC-MV administration), although improvements in whole-gut transit and defecation frequency were not sustained, a significant benefit in distal colonic transit remained (Figure 14J).

Finally, we assessed the safety and immunogenicity of BMSC-MVs. No significant increase in total IgG was observed (Supplemental Figure 8A), indicating low humoral immunogenicity. Serum analysis showed that BMSC-MVs reduced alanine aminotransferase in diabetic mice without affecting aspartate aminotransferase or renal markers (Supplemental Figure 8B). In the intestine, BMSC-MVs downregulated proinflammatory cytokines *Il1b* and *Il6* but did not alter *Tnfa* or antiinflammatory cytokines *Il4*, *Il10*, and *Il13* (Supplemental Figure 8C). Collectively, these results support the biocompatibility, low immunogenicity, and sustained functional efficacy of BMSC-MVs as a promising therapeutic strategy for diabetes-induced ENS injury.

## Discussion

Diabetic gastroenteropathy (manifesting as pain, nausea, constipation, and diarrhea) is a prevalent and challenging complication (27). A key driver of these symptoms is structural ENS injury due to diabetes itself, independent of potential medication effects, as directly evidenced by ENS depletion in patients, particularly those with constipation (28–30). Consequently, regenerative strategies aimed at reversing these structural abnormalities in ENS are fundamentally required. Here, we identified ER stress and the pro-apoptotic PERK branch of the UPR activation in ENPCs as key mechanisms impairing their neurogenic and gliogenic capacity under diabetic conditions. We further demonstrated that BMSC-MVs effectively restore ENS integrity and gastrointestinal motility in diabetic models. Mechanistically, surface VCL on BMSC-MVs binds to TLN1 on ENPCs, activating the ERK signaling pathway, which in turn suppresses ER stress and the pro-apoptotic PERK branch, thereby rejuvenating ENPC-mediated repair and ameliorating diabetes-induced ENS injury.

Postnatal enteric neurogenesis is known to persist in rodents, with the adult ENS maintaining intrinsic regenerative capacity largely mediated by resident ENPCs (31). Similar to the activation of endogenous neural stem cells following central nervous system injury (32), the ENS exhibits limited neurogenic activity under physiological conditions but can initiate reparative neurogenesis in response to injury stimuli (33). For example, gut microbiota depletion has been shown to induce nonselective neuronal loss and subsequent neurogenesis in the myenteric plexus (34), while psychological stress increases the proportion of HuC/D^+^NESTIN^+^ neurons in whole-mount myenteric plexus preparations (35). In line with these findings, we observed that diabetes induced a limited degree of neurogenic and gliogenic differentiation from ENPCs, which was nevertheless insufficient to counterbalance the overall neuronal and glial loss. Treatment with BMSC-MVs markedly enhanced ENPC differentiation, leading to a substantial recovery of neurons and glial cells in the diabetic ENS. Although the therapeutic potential of BMSC-MVs has been previously documented in central and peripheral nerve injury models (36, 37), our study is the first to establish their efficacy in promoting ENS repair. Together with their demonstrated biocompatibility and low immunogenicity in vivo, BMSC-MVs represent a promising cell-free therapeutic strategy not only for diabetes-induced ENS injury but potentially for other ENS-related gastrointestinal motility dysfunctions.

The ER is a central organelle responsible for protein folding, lipid synthesis, and calcium homeostasis. Its function can be disrupted by metabolic insults such as nutrient imbalance and oxidative stress, leading to accumulation of misfolded proteins and ER stress. In response, the UPR is activated to restore proteostasis by enhancing folding capacity and clearing misfolded proteins; however, if stress persists, the UPR shifts to pro-apoptotic signaling (38). Diabetes, a metabolic disorder characterized by chronic hyperglycemia, induces widespread cellular metabolic perturbations that promote ER stress (39, 40). Although ER stress and the UPR have been extensively implicated in the pathogenesis of various diabetic complications, including retinopathy (41), nephropathy (42), cardiomyopathy (43), and peripheral neuropathy (44), their role in diabetic enteric neuropathy remains poorly defined. Our study addresses this gap through transcriptomic analysis of colonic tissues from human and murine diabetic models, which revealed significant enrichment of differentially expressed genes associated with ER stress and UPR signaling. Consistent with these findings, we demonstrated that high glucose activates the ER stress sensor GRP78 and selectively induces the pro-apoptotic PERK/EIF2α/ATF4 branch of the UPR in ENPCs. This was accompanied by upregulation of the downstream effector CHOP, which promoted expression of the pro-apoptotic protein BAX while suppressing the anti-apoptotic protein BCL-2, collectively contributing to ENPC injury. Importantly, treatment with BMSC-MVs effectively suppressed ER stress and inhibited the PERK pathway, thereby establishing the ER stress/UPR axis as a key mechanistic contributor to diabetic enteric neuropathy.

The role of ERK signaling in diabetes and its complication exhibits notable tissue-specific variability. In the kidney, high glucose promotes ERK activation, contributing to mesangial fibrosis and podocyte apoptosis (45, 46), whereas in pancreatic β cells, diabetes suppresses ERK activity, exacerbating β cell dysfunction (47), and no significant change is observed in sciatic nerves of diabetic patients (48). In our study, we found that diabetes activates the ERK pathway in ENPCs. ERK inhibition worsened high glucose–induced ENPC injury, ER stress, and PERK/EIF2α/ATF4-mediated apoptosis and abolished the restorative effects of BMSC-MVs, indicating that BMSC-MVs promote ENS repair via ERK activation and that diabetes-induced ERK signaling represents an adaptive, protective response. This interpretation aligns with reports that enhanced ERK phosphorylation in diabetic dorsal root ganglia represents an adaptive response, and activated ERK signaling through glucagon-like peptide-1–prevented neural dysfunction (49). Similarly, studies on JNK — another MAPK family member — have shown that its activation in diabetic sensory neurons may be neuroprotective rather than detrimental, as treatment with the antioxidant α-lipoic acid, known to normalize nerve conduction velocity deficits in diabetic rats, further enhanced JNK activation (50). Collectively, these findings support a context-dependent protective role of ERK in diabetic neuropathy, consistent with the modest increase in neuroglial differentiation of ENPCs under diabetic conditions — an adaptive but insufficient response — whereas BMSC-MVs therapeutically enhance ERK signaling, effectively suppressing ER stress and restoring ENPC regenerative function.

Building on our findings that BMSC-MVs activate the ERK pathway in ENPCs, we further explored the underlying regulatory mechanisms. EV membrane proteins are known to govern their biodistribution and bioactivity (51). In this study, comprehensive protein interaction screening identified VCL on BMSC-MVs as a key surface ligand that physically associates with TLN1 on ENPCs. Genetic perturbation and functional rescue experiments established that the VCL-TLN1 axis is essential not only for the cellular internalization of BMSC-MVs but also for the subsequent activation of the ERK signaling pathway, which ultimately alleviates ER stress and promotes ENPC differentiation. TLN1 functions as a membrane-anchored mechanosensitive adaptor that mediates integrin activation and cytoskeletal reorganization in response to mechanical stimuli. Its rod domain contains cryptic VCL-binding sites that become exposed under mechanical tension (52). In this context, the engagement of VCL on BMSC-MVs with TLN1 on ENPCs may mimic the mechanical stimulation typically provided by substrate adhesion. This interaction likely initiates biochemical signaling through integrin-mediated recruitment of upstream ERK pathway regulators (53), providing a plausible mechanistic basis for ERK activation and the subsequent functional recovery observed in the diabetic ENS. Thus, beyond facilitating vesicle uptake, VCL-TLN1 engagement serves as an initiating signaling event that triggers the therapeutic action of BMSC-MVs.

In summary, our findings establish that BMSC-MVs ameliorate diabetes-induced ENS injury and restore gastrointestinal motility via VCL binding to TLN1 on ENPCs. This interaction facilitates cellular internalization and activates the ERK pathway, thereby attenuating ER stress and promoting ENPC differentiation. These insights not only advance the mechanistic understanding of diabetic gastrointestinal dysmotility but also support the therapeutic potential of EV-based strategies for treating ENS disorders.

## Methods

### Sex as a biological variable.

All animal studies included equal representations of male and female mice. Both male and female patients were involved for sample collection.

### Patient samples.

Preoperative medical records were reviewed for documented history of diabetes, and patients with fasting plasma glucose level ≥ 126 mg/dL or hemoglobin A1c values ≥ 6.5% were considered diabetic. Human colon tissue samples were procured from patients undergoing colectomy in Wuhan Union Hospital. These samples were obtained from macroscopically normal margins adjacent to the pathological area, as confirmed by a pathologist. Written informed consent was obtained from each patient prior to tissue collection. The basic information of patients is provided in Supplemental Table 3.

### Animals.

Nestin-creER^T2^ mice were bred with R26-e (CAG-RSR-LSL-DTRGFP-WPRE-pA) and Ngfr-e (2A-DreER^T2^) mice (purchased from Shanghai Model Organisms Center) to create Nestin-creER^T2^ × Ngfr-DreER^T2^:DTRGFP mice. The Nestin-creER^T2^ × Ngfr-DreER^T2^:DTRGFP mice represent a dual-inducible lineage tracing model. As previously established in our laboratory (13), administration of tamoxifen (10540-29, Sigma-Aldrich) induces specific and heritable labeling of ENPCs and their progeny with GFP. Tamoxifen was prepared in corn oil (100 μL) and administered via intraperitoneal injection for 6 consecutive days to achieve efficient recombination. In the transgenic diabetic mouse model, tamoxifen was administered starting at 6 weeks and 4 days after diabetes induction. Following the final tamoxifen injection, a 3-day rest period was observed before the mice received BMSC-MV treatment. WT C57BL/6J mice were purchased from Beijing SPF Biotech. Pregnant C57BL/6J mice (E15-E18) were obtained from the Hubei Provincial Center for Disease Control and Prevention Experimental Animal Research Center. Mice were maintained on a light/dark cycle (12 hours/ 12 hours) in a temperature-controlled (21°C–25°C), humidity-controlled (50%–60%), and specific pathogen–free condition room.

### Isolation of BMSC-MVs.

Mouse BMSCs were purchased from Cyagen Biosciences (MUBMX-01001) and cultured in DMEM (11885084, Gibco) supplemented with 10% FBS (10099141C, Gibco) at 37 °C and 5% CO_2_. After the BMSC culture reached a 60%–70% confluence, the cells were cultured in a complete medium supplemented with 10% MV-depleted FBS (FBS centrifuged at 100,000*g* for 12 hours) for another 48 hours. The MVs were isolated using a differential centrifugation protocol. Briefly, the cell supernatants were collected and centrifugated at 600*g* for 10 minutes to remove dead cells. The supernatants were then centrifugated at 2,000*g* for 30 minutes to remove cellular debris and apoptotic bodies and finally centrifugated at 20,000*g* for 75 minutes to pellet the MVs. The MV pellet was washed with PBS under the same ultracentrifuge conditions and stored at –80°C before use.

### Grouping and BMSC-MV transplantation.

Mice (6–8 weeks old) fasted overnight were injected with 100 μL of streptozotocin (150 mg/kg; S0130, Sigma-Aldrich) dissolved in citrate buffer (pH 4.5; C1013, Solarbio), while the control group was given the same volume of citrate buffer. After 1 week, mice with blood glucose ≥ 16.7 mmol/L were considered as successful induction of diabetes and included in the subsequent study. Following the establishment of the diabetic model (8 weeks after streptozotocin injection), both control and diabetic mice were randomly assigned and intraperitoneally administered 40 μg of BMSC-MVs in PBS once every 4 weeks for 8 weeks, resulting in a total cumulative dose of 80 μg per mouse.

### Isolation and identification of ENPCs.

The entire gut was obtained from embryonic (E15–E18) mice dissociated enzymatically in digestion buffer (consisting of Hank’s balanced salt solution [G4204, Servicebio], 1 mg/mL dispase II [4942078001, Roche], 300 U/mL collagenase IV [11088866001, Roche], 40 U/mL DNase I [10104159001, Roche], and 0.1% BSA [GC305006, Servicebio]) for 40 minutes at 37°C. Cell suspensions filtered through a 40 μm mesh, centrifuged, and resuspended in neurosphere medium (Neurobasal A medium [A2477501, Gibco] supplemented with 1% B27 [05731, STEMCELL Technologies], 20 ng/mL fibroblast growth factor [450-33, Peprotech], 20 ng/mL epidermal growth factor [315-09, Peprotech], and 1% penicillin/streptomycin [15070063, Gibco]). Neurospheres formed after 5–7 days in culture, and immunostaining characterization confirmed that the cells within the neurospheres were positive for ENPC markers (NESTIN and NGFR) and negative for TUJ1 and GFAP. ENPCs were then passaged using accutase (07920, STEMCELL Technologies), and the second generation of ENPCs were used for experiments.

### Drug treatment.

For in vivo experiments, 4-PBA (1 g/kg; P21005, Sigma-Aldrich) was dissolved in saline solution and administered to mice once a day for 8 continuous weeks after successful modeling of diabetes for 8 weeks. For in vitro experiments, primary ENPCs were exposed with d-glucose (30 mM; PB180418, Procell), BMSC-MVs (0.25 μg/mL), 4-PBA (5 mM), and ERK inhibitor PD98059 (10 μM; HY-12028, MedChemExpress) for 72, 24, 4, and 2 hours.

### Use of generative artificial intelligence.

We used ChatGPT (OpenAI, GPT-5) during October–November 2025 solely for language polishing of the Discussion. No scientific content, data, or conclusions were generated or altered by artificial intelligence (AI). All AI-assisted content was reviewed and approved by the authors, who take full responsibility for the manuscript.

Additional details are available in Supplemental Methods and Supplemental Tables 4–6.

### Statistics.

Data are expressed as mean ± SD. Statistical analyses were performed using GraphPad Prism 8.0.1 (GraphPad Software). The normality of data distribution was assessed using the Shapiro-Wilk test prior to analysis. Continuous variables were analyzed using parametric tests if normality was satisfied: unpaired 2-tailed Student’s *t* test for comparisons between 2 groups and 1-way ANOVA with Tukey’s post hoc test for comparisons among multiple groups with a designated control. For continuous data that violated the normality assumption, nonparametric tests were applied: the Mann-Whitney *U* test for 2-group comparisons or the Kruskal-Wallis test with Dunn’s post hoc test for multiple comparisons. Categorical variables were compared using the χ^2^ test. A *P* value less than 0.05 was considered significant, and statistically significant differences were defined as *P < 0.05, **P < 0.01, ***P < 0.001, and ****P < 0.0001. The *n* values in the figure legends indicate the number of biologically independent replicates. The results of Western blot and immunofluorescence staining are representative of at least 3 biologically independent replicates. Unless otherwise specified, all outcomes were replicated independently more than 3 separate times, producing similar results.

### Study approval.

The protocol for human experiments was approved by the Ethics Committee of Tongji Medical College, Huazhong University of Science and Technology (IORG number S147). All procedures were carried out in accordance with the Declaration of Helsinki. The protocol for animal experiments was approved by the IACUC of Union Hospital at Tongji Medical College, Huazhong University of Science and Technology (IACUC number S2804).

### Data availability.

The raw sequence data reported in this paper have been deposited in the Genome Sequence Archive of the China National Center for Bioinformation (accession number PRJCA052122) (54, 55). Values for all data points in graphs are reported in the Supporting Data Values file. Figure 13A, Figure 13B, and the graphical abstract were created in BioRender (agreement number EK299KUQH7).

## Author contributions

HS, HY, and RL were responsible for the study concept and design. HS, HY, YL, and MF performed experiments and drafted the manuscript. SC, SX, CJ, YZ, and WJ supervised part of the study and discussed data. RL provided critical revision of the manuscript and important intellectual content. RL and WQ supervised the study. RL, HS, HY, and WJ acquired funding for the work. The order of co–first authors was determined by the volume of work each contributed to the study. All authors reviewed the manuscript.

## Funding support

- National Key Research and Development Program (2023YFC2307001).
- National Natural Science Foundation of China (82100569, 82300616, and 82100563).
- China Postdoctoral Science Foundation (2024M761067).

## Supplementary Material

Supplemental data

Unedited blot and gel images

Supporting data values

## Version 1 03/16/2026

Electronic publication

## References

[1] Collaboration NCDRF (2024). Worldwide trends in diabetes prevalence and treatment from 1990 to 2022: a pooled analysis of 1108 population-representative studies with 141 million participants. Lancet.

[2] Collaboration NCDRF (2016). Worldwide trends in diabetes since 1980: a pooled analysis of 751 population-based studies with 4.4 million participants. Lancet.

[3] Sun H (2022). IDF Diabetes Atlas: global, regional and country-level diabetes prevalence estimates for 2021 and projections for 2045. Diabetes Res Clin Pract.

[4] Wei L (2023). Constipation in DM are associated with both poor glycemic control and diabetic complications: current status and future directions. Biomed Pharmacother.

[5] Eid SA (2023). New perspectives in diabetic neuropathy. Neuron.

[6] Anitha M (2006). GDNF rescues hyperglycemia-induced diabetic enteric neuropathy through activation of the PI3K/Akt pathway. J Clin Invest.

[7] Azpiroz F (2016). Diabetic neuropathy in the gut: pathogenesis and diagnosis. Diabetologia.

[8] Wegeberg AM (2022). Gastrocolic reflex is delayed and diminished in adults with type 1 diabetes. Dig Dis Sci.

[9] Abdalla MMI (2024). Enteric neuropathy in diabetes: implications for gastrointestinal function. World J Gastroenterol.

[10] Laranjeira C (2011). Glial cells in the mouse enteric nervous system can undergo neurogenesis in response to injury. J Clin Invest.

[11] Kulkarni S (2017). Adult enteric nervous system in health is maintained by a dynamic balance between neuronal apoptosis and neurogenesis. Proc Natl Acad Sci U S A.

[12] D’Errico F (2018). Estrogen receptor β controls proliferation of enteric glia and differentiation of neurons in the myenteric plexus after damage. Proc Natl Acad Sci U S A.

[13] Fan M (2023). BMSCs promote differentiation of enteric neural precursor cells to maintain neuronal homeostasis in mice with enteric nerve injury. Cell Mol Gastroenterol Hepatol.

[14] Staff NP (2019). Mesenchymal stromal cell therapies for neurodegenerative diseases. Mayo Clin Proc.

[15] Dabrowska S (2019). Human bone marrow mesenchymal stem cell-derived extracellular vesicles attenuate neuroinflammation evoked by focal brain injury in rats. J Neuroinflammation.

[16] Giovannelli L (2023). Mesenchymal stem cell secretome and extracellular vesicles for neurodegenerative diseases: risk-benefit profile and next steps for the market access. Bioact Mater.

[17] Kalra H (2016). Focus on extracellular vesicles: introducing the next small big thing. Int J Mol Sci.

[18] Teixeira FG (2013). Mesenchymal stem cells secretome: a new paradigm for central nervous system regeneration?. Cell Mol Life Sci.

[19] L PK (2019). The mesenchymal stem cell secretome: a new paradigm towards cell-free therapeutic mode in regenerative medicine. Cytokine Growth Factor Rev.

[20] Witwer KW (2019). Defining mesenchymal stromal cell (MSC)-derived small extracellular vesicles for therapeutic applications. J Extracell Vesicles.

[21] Pincela Lins PM (2023). Manufacture of extracellular vesicles derived from mesenchymal stromal cells. Trends Biotechnol.

[22] Shi H (2021). CD44 fucosylation on bone marrow-derived mesenchymal stem cells enhances homing and promotes enteric nervous system remodeling in diabetic mice. Cell Biosci.

[23] Hetz C (2020). Mechanisms, regulation and functions of the unfolded protein response. Nat Rev Mol Cell Biol.

[24] van Niel G (2022). Challenges and directions in studying cell-cell communication by extracellular vesicles. Nat Rev Mol Cell Biol.

[25] Phoenix TN (2010). Spred1, a negative regulator of Ras-MAPK-ERK, is enriched in CNS germinal zones, dampens NSC proliferation, and maintains ventricular zone structure. Genes Dev.

[26] Lee HR (2019). Differential effects of MEK inhibitors on rat neural stem cell differentiation: repressive roles of MEK2 in neurogenesis and induction of astrocytogenesis by PD98059. Pharmacol Res.

[27] Du YT (2018). Gastrointestinal symptoms in diabetes: prevalence, assessment, pathogenesis, and management. Diabetes Care.

[28] Yarandi SS (2014). Diabetic gastrointestinal motility disorders and the role of enteric nervous system: current status and future directions. Neurogastroenterol Motil.

[29] Kornum DS (2025). Diabetic gastroenteropathy: a pan-alimentary complication. Diabetologia.

[30] Chandrasekharan B (2011). Colonic motor dysfunction in human diabetes is associated with enteric neuronal loss and increased oxidative stress. Neurogastroenterol Motil.

[31] Burns AJ (2014). Neural stem cell therapies for enteric nervous system disorders. Nat Rev Gastroenterol Hepatol.

[32] Llorens-Bobadilla E (2020). A latent lineage potential in resident neural stem cells enables spinal cord repair. Science.

[33] Stavely R (2024). Mature enteric neurons have the capacity to reinnervate the intestine with glial cells as their guide. Neuron.

[34] Vicentini FA (2021). Intestinal microbiota shapes gut physiology and regulates enteric neurons and glia. Microbiome.

[35] Schneider KM (2023). The enteric nervous system relays psychological stress to intestinal inflammation. Cell.

[36] Li Y (2023). Comparative study of extracellular vesicles derived from mesenchymal stem cells and brain endothelial cells attenuating blood-brain barrier permeability via regulating Caveolin-1-dependent ZO-1 and Claudin-5 endocytosis in acute ischemic stroke. J Nanobiotechnology.

[37] Shi S (2025). Extracellular vesicles in peripheral nerve regeneration: from biology to therapeutic engineering. Int J Nanomedicine.

[38] Chen X (2023). Endoplasmic reticulum stress: molecular mechanism and therapeutic targets. Signal Transduct Target Ther.

[39] Lu X (2024). Type 2 diabetes mellitus in adults: pathogenesis, prevention and therapy. Signal Transduct Target Ther.

[40] Lytrivi M (2025). Diabetes mellitus and the key role of endoplasmic reticulum stress in pancreatic β cells. Nat Rev Endocrinol.

[41] Zhang SX (2024). The endoplasmic reticulum: homeostasis and crosstalk in retinal health and disease. Prog Retin Eye Res.

[42] Wang J (2025). Intermodulation of endoplasmic reticulum stress and ferroptosis in diabetic nephropathy: molecular mechanisms and therapeutic potentials. Apoptosis.

[43] Congur I (2025). Targeting endoplasmic reticulum stress as a potential therapeutic strategy for diabetic cardiomyopathy. Metabolism.

[44] Ye S (2024). Different types of cell death in diabetic neuropathy: a focus on mechanisms and therapeutic strategies. Int J Mol Sci.

[45] Lin CL (2006). Ras modulation of superoxide activates ERK-dependent fibronectin expression in diabetes-induced renal injuries. Kidney Int.

[46] Wang Y (2012). Vitamin D receptor signaling in podocytes protects against diabetic nephropathy. J Am Soc Nephrol.

[47] Khatri R (2021). Intrapancreatic MSC transplantation facilitates pancreatic islet regeneration. Stem Cell Res Ther.

[48] Li CJ (2012). Cardiac fibrosis and dysfunction in experimental diabetic cardiomyopathy are ameliorated by alpha-lipoic acid. Cardiovasc Diabetol.

[49] Jolivalt CG (2011). GLP-1 signals via ERK in peripheral nerve and prevents nerve dysfunction in diabetic mice. Diabetes Obes Metab.

[50] Price SA (2003). Activation of JNK in sensory neurons protects against sensory neuron cell death in diabetes and on exposure to glucose/oxidative stress in vitro. Ann N Y Acad Sci.

[51] Rai A (2021). Proteomic dissection of large extracellular vesicle surfaceome unravels interactive surface platform. J Extracell Vesicles.

[52] Dedden D (2019). The architecture of talin1 reveals an autoinhibition mechanism. Cell.

[53] Pulous FE (2021). Talin-dependent integrin activation is required for endothelial proliferation and postnatal angiogenesis. Angiogenesis.

[54] CNCB-NGDC Members and Partners (2025). Database resources of the National Genomics Data Center, China National Center for Bioinformation in 2025. Nucleic Acids Res.

[55] Zhang S (2025). The GSA Family in 2025: a broadened sharing platform for multi-omics and multimodal data. Genomics Proteomics Bioinformatics.

