# Supplementary material for: BM-derived mesenchymal stem cell microvesicles protect enteric neural precursor cells and alleviate diabetes-associated enteric neuropathy
Source: J Clin Invest. 2026 Mar 16;136(6):e192437. doi: 10.1172/JCI192437 (PMC12987631; doi:10.1172/JCI192437)
Supplement: Supplemental data [file jci-136-192437-s027.pdf]

Supplemental Methods

Analysis of bowel symptoms in diabetic individuals using NHANES data

The NHANES is a crucial research program that aims to evaluate the health and nutritional status of both adults and children residing in the United States. The Centers for Disease Control and Prevention is responsible for furnishing health statistics for the nation, and the protocols of NHANES have been duly approved by the Research Ethics Review Board of National Center for Health Statistics. To ensure the protection of the participants' rights, NHANES has obtained informed written consent from all the individuals involved in the study. Moreover, the datasets generated and analyzed in the current study are readily available on the official NHANES website (https://www.cdc.gov/nchs/nhanes/index.html). We downloaded data of NHANES from 2005 to 2010.

According to the American Diabetes Association diagnostic criteria, diabetes was defined as meeting any of the following criteria: a self-reported physician diagnosis, current use of insulin or oral hypoglycemic agents, a fasting plasma glucose level ≥ 126 mg/dL, or a HbA1c level ≥ 6.5%. Prediabetes is identified by self-reported prediabetes status or having fasting blood glucose between 100 mg/dL and 125 mg/dL, or HbA1c between 5.7% and 6.4% (1). The Bowel Health Questionnaire was used to identify subjects with chronic diarrhea or chronic constipation (2). The usual or most common stool of Bristol Stool Form Scale type 1 or type 2, or bowel movements less than 3 times a week was defined as chronic constipation. The usual or most common stool of Bristol Stool Form Scale type 6 or type 7 or bowel movements more than three times a day was defined as chronic diarrhea. The rest Bristol Stool Form Scale type 3, type 4, type 5, or other bowel movements were designated as no bowel symptoms. Individuals with self-reported history of inflammatory bowel disease, celiac disease, and/or colon cancer were excluded from the analysis.

Assessment of gastrointestinal transit times in patients

This study retrospectively analyzed data from patients and healthy controls who underwent magnetically controlled capsule endoscopy at the Endoscopy Center of Wuhan Union Hospital

between 2019 and 2024. The diabetic group comprised individuals with type 1 or type 2 diabetes with disease duration >5 years, while controls had no dyspeptic symptoms, known gastroparesis, or suspected gastrointestinal obstruction. All participants had documented negative upper and lower gastrointestinal endoscopy before examination and no contraindications to capsule endoscopy (Supplemental Table 4). Exclusion criteria for both groups included irritable bowel syndrome, gastrointestinal tumors, inflammatory bowel disease, prior gastrointestinal resection, or use of medications affecting gastrointestinal motility. Gastrointestinal transit times were assessed as follows: stomach transit time (capsule ingestion to pylorus passage), small-intestine transit time (pylorus to ileocecal junction), colonic transit time (ileocecal junction to capsule expulsion), and whole gastrointestinal transit time (ingestion to expulsion).

Characterization of BMSCs-MVs

The morphology of BMSCs-MVs was examined by TEM (HT7800, Hitachi) at 80 kV. The particle concentration and size were tracked by NTA (ZetaView, Particle Metrix). Protein concentration was quantified using a BCA assay (P0010, Beyotime). Furthermore, the expression of both positive BMSCs-MVs markers (CD9, CD63, TSG101) and a negative marker (CALNEXIN) was assessed by Western blot analysis.

Tracing of BMSCs-MVs in vivo

20 μg BMSCs-MVs labeled with DiR (UR21017, Umibio) or PBS were intraperitoneally injected into mice. After 4 and 24 hours, bioluminescence imaging (Bruker MS FX Pro, Bruker Corp.) was conducted with Ex 748 nm/Em 780 nm to track BMSCs-MVs distribution in vivo. In addition, mice were sacrificed at predetermined time points to collect major organs (brain, lung, heart, spleen, liver, stomach, small intestine, colon, and kidney) for biodistribution assessment.

Safety of BMSCs-MVs

Peripheral blood was collected from the orbit of euthanized mice and allowed to clot at room temperature for 30 minutes. Following clotting, the samples were centrifuged at 3000 ×g for 15 minutes to isolate the serum. The resulting serum samples were stored at -80 °C for subsequent analysis of liver function (aspartate aminotransferase: C010-2-1; alanine aminotransferase: C009-2-1) and renal function (creatinine: C011-2-1; BUN: C013-2-1) using appropriate kits (Nanjing Jiancheng Bioengineering Institute) following the manufacturer's instructions.

Systemic immunogenicity and local inflammatory responses of BMSCs-MVs

The systemic immunogenicity of BMSCs-MVs was evaluated by measuring the concentration of mouse IgG in serum samples with a commercial enzyme-linked immunosorbent assay kit (RK00375, ABlconal), according to the manufacturer's instructions. The local impact of BMSCs-MVs on the intestinal tissue was assessed by quantifying the transcriptional levels of pro-inflammatory (*Tnfa, Il1b,* and *Il6*) and anti-inflammatory (*Il4, Il10,* and *Il13*) cytokines using qRT-PCR.

Assessment of gastrointestinal motility in vivo

Functional outcomes on gastrointestinal motility were assessed by counting the number of fecal pellet output, measuring the whole gut transit time and distal colon bead expulsion time. The mice were fasted overnight with free access to water. The next day, mice were placed individually in new bedding-free cages and had free access to food and water. Fecal pellets for a 5 hour-period were collected and counted the total number. To measure whole gut transit time, overnight fasted mice were gavaged with 300 µL of the carbon black suspension (5% powdered carbon suspended in 0.9% saline solution) and monitored every 10 minutes. The period from the time of the gavage to the visualization of the first fecal pellet containing the black carbon

was considered the whole gut transit time. To measure the distal colonic transit time, the mice were anesthetized with isoflurane. A glass bead in diameter of 3 mm was inserted 3 cm into the distal colon, and the latency to bead expulsion was recorded as the colonic transit time.

Organ bath colonic smooth muscle activity study

Freshly excised distal colonic smooth muscle strip (1 cm in length and 3 mm in width) was placed in the 25 mL organ bath system containing oxygenated (95% O_2 and 5% CO_2) Krebs' solution (NaCl 119 mmol/L, KCl 4.7 mmol/L, $NaHCO_3$ 25 mmol/L, NaH_2PO_4 1.2 mmol/L, $MgSO_4$ 1.2 mmol/L, glucose 11.1 mmol/L, $CaCl_2$ 2.5 mmol/L, pH 7.30–7.40) at 37°C. The colonic smooth muscle strip was mounted between two L-shape hooks attached to the TRI201AD Isometric Transducer (AD Instruments, Australia). Then, the muscle strip was equilibrated for 30 minutes under a basal tension of 1g. Electric field stimulation (EFS) was applied to the colon segment, and the voltage (20V), pulse (10s), and gradient stimulation frequency (2, 4, 8, 16, 32, and 64 Hz) were controlled by LabChart software v8.0 (AD Instruments, Australia). Force contraction of the circular smooth muscle was recorded and analyzed by using LabChart software v8.0.

Cellular uptake of BMSCs-MVs

ENPCs and LMMP were cocultured with BMSCs-MVs labeled with DiI (C1036, Beyotime) for different periods. After that, cells were washed with PBS and fixed with 4% paraformaldehyde for 15 minutes. ENPCs were stained with FITC-phalloidin (G1028, Servicebio), while LMMP were stained with antibodies directed against GFP, HuC/D, and GFAP. Subsequently, ENPCs and LMMP were counterstained with 4',6-diamidino-2-phenylindole (DAPI; G1012, Servicebio), and imaged by a confocal laser microscope (Nikon, Tochigi, Japan).

Cell proliferation, apoptosis, and differentiation assays

Cell Counting Kit-8 (CCK-8) and EdU incorporation experiment were used to assess ENPCs proliferation ability. ENPCs were seeded at a density of 4000 cells/well in a 96-well plate overnight and treated as indicated. After 72 hours of treatment, 10 μL CCK-8 solution (G4104, Servicebio) was added to each well and incubated in the dark for 2 hours. The absorbance at 450 nm was measured using a microplate reader (Tiangen, China). Samples were prepared at least in triplicates. For EdU incorporation assay, ENPCs were cultured in the presence of EdU (10 μM) for 4 hours, and stained using the BeyoClick™ EdU Cell Proliferation Kit with Alexa Fluor 594 (C0078S, Beyotime) according to the manufacturers' instructions.

The apoptosis assay was analyzed using the Annexin V-FITC Apoptosis Detection Kit (C1062, Beyotime). ENPCs were harvested after incubating with BMSCs-MVs for 24 hours, washed in cold PBS, and resuspended in binding buffer containing Annexin V-FITC and propidium iodide (PI) according to the manufacturer's instructions. Subsequently, cells were detected by flow cytometry (BD, Biosciences) and analyzed by software Flowjo v10.7.1.

For the differentiation assay, neurospheres were plated on poly-L-lysine-coated (0.1 mg/mL; Sigma, P1399) 24-well plate and cultured with differentiation medium (Neurobasal A medium supplemented with 1% B27 and 5% fetal bovine serum) for 7 days. Cells were fixed in 4% paraformaldehyde, and were further immunostaining for neuronal (HuC/D, nNOS) or glial (GFAP) markers.

Cell transfection

The targeting sequences of mouse siRNAs designed against TLN1-encoding transcripts were

as follows: *Tln1*: 5′-GUAAUCUCUGCUAAGACAA-3′; 5′-UUGUCUUAGCAGAGAUUAC-3′, which was commercially obtained from Beijing Tsingke Biotech Co., Ltd. The FLAG-tagged *Tln1* overexpression plasmid (pECMV-Tln1-m-FLAG, P6342) was obtained from MiaoLing Plasmid Sharing Platform. ENPCs were then transfected with either the siRNA or the plasmid using Lipofectamine 3000 (L3000008, Thermo Fisher Scientific) in accordance with the manufacturer's instructions. ENPCs were trypsinized with acctutase and resuspended with culture medium, and plated in 24-well plates. Then, siRNA or plasmid and Lipofectamine 3000 were diluted in Opti-MEM (31985070, Gibco), mixed, and incubated for 15 minutes before being added to cells. After 24 h, transfection medium was replaced with complete medium. After 48-72 hours, gene knockdown or overexpression efficiency was assessed by qRT-PCR and western blot.

Vcl knockdown in BMSCs was performed using the CRISPR-Cas9 system. Lentivirus was produced by co-transfecting HEK293FT cells with packaging plasmids (3 μg pMD2.G, 9 μg psPAX2) and 12 μg of sgRNA expression vectors (control or targeting murine *Vcl*) using PEI. After 48–72 hours, viral supernatant was collected and used to infect target cells in the presence of polybrene for 24 hours. For *Vcl* overexpression, an HA-tagged construct was generated by amplifying the full-length murine vcl cDNA using Q5® High-Fidelity DNA Polymerase (M0491, New England Biolabs). The product was assembled into a PiggyBac transposon vector (Yunzhou Biotech) with the NEBuilder HiFi DNA Assembly Master Mix (E2621, New England Biolabs) for 1 hour at 50 °C. The assembly reaction was transformed into competent *E. coli* cells. Successful amplification and linearization were confirmed by agarose gel electrophoresis. Positive clones were verified by Sanger sequencing. Transduced cells were selected with puromycin for 7 days before assessing knockdown or overexpression efficiency.

Immunofluorescence analysis

Colon was opened along the mesenteric border, pinned on a sylgard-coated plate, and peeled off of the mucosa and submucosa to expose the LMMP. Next, LMMP and ENPCs were fixed with 4% paraformaldehyde for 20 minutes at room temperature, and blocked with donkey serum containing 0.3% Triton X-100. Primary antibodies (Supplemental Table 5) were diluted in 10% donkey serum and incubated at 4°C. After washing with PBS, the sample was incubated with the secondary antibody (Supplemental Table 5) for 2 hours. Cell nuclei were stained with DAPI. Finally, samples were mounted onto glass slides and imaged using a confocal laser microscope (Nikon, Japan).

Western blot

The cells and colon were lysed in radio immunoprecipitation assay lysis buffer (G2002, Servicebio) supplemented with 1% protease inhibitor cocktail (HYK0010, MedChemExpress). Protein concentrations were determined by BCA assay. Equal amounts of protein were separated by 10% or 12.5% sodium dodecyl sulfate–polyacrylamide gel, and then transferred to polyvinylidene fluoride membranes. After blocking with 10% nonfat milk for 2 hours, membranes were incubated with specific primary antibody overnight at 4°C, and subsequently incubated with horseradish peroxidase-conjugated secondary antibody for 1 hour at 37°C. The protein complex was visualized using enhanced chemiluminescence reagents (E412, Vazyme) by the Chemiluminescence imaging system (UVP, USA). The intensity of the band was quantified using ImageJ software v1.52. Information on the antibodies is listed in Supplemental Table 5.

Assessment of global protein synthesis

The global protein synthesis rate was evaluated using the Surface Sensing of Translation method (3). In brief, ENPCs were treated with 10 μg/mL puromycin (ST551, Beyotime) in culture medium for 30 minutes to allow for a brief pulse-labeling of nascent polypeptides.

Subsequently, the cells were lysed, and the incorporation of puromycin was detected by Western blot analysis using an anti-puromycin antibody.

Protein aggregation detection assay

PROTEOSTAT Aggresome Detection Kit (ENZ-51035-K100, Enzo Life Sciences) was used to detect misfolded or aggregated proteins in cells as previously described (4). Briefly, cells grown on coverslips were washed with PBS, fixed with 4% formaldehyde for 30 minutes at room temperature, and permeabilized with permeabilization solution (0.5% Triton X-100 supplemented with 3 mM EDTA) on ice with gentle agitation for 30 minutes. Subsequently, the cells were stained with PROTEOSTAT dye (1:20000) at room temperature for 30 minutes, followed by three washes with PBS. Nuclei were counterstained with DAPI. Imaging was performed using a confocal laser microscope (Nikon, Japan).

Biotin pull-down assay

The biotin pull-down assay was performed as the reference (5). Surface proteins of ENPCs / BMSCs-MVs were biotinylated with 1 mM EZ-Link Sulfo-NHS-LC-Biotin (A39257, Thermo Fisher Scientific) at 4°C for 2 hour according to the manufacturer's instructions. The reaction was quenched by 100 mM TRIS pH 8 and incubated for 15 min. Then proteins of biotin-labelled ENPCs / BMSCs-MVs were extracted with PBS containing 1% Triton X-100 for 1 hour on ice. To perform the binding assay, biotinylated ENPCs / BMSCs-MVs surface proteins and BMSCs-MVs / ENPCs surface proteins were incubated overnight at 4°C. Next, the mixed complex was incubated with streptavidin magnetic beads (P2151, Beyotime) for 16 hours at 4°C. The beads were then washed three times and incubated with elution buffer for 5 minutes, followed by centrifugation. The eluates were analyzed by mass spectrometry for protein identification, or subjected to SDS-polyacrylamide gel electrophoresis, visualized by silver staining (P0017, Beyotime) and analyzed by mass spectrometry.

Far Western blot assay

The far Western blot assay was performed as the reference (6). The surface proteins of ENPCs / BMSCs-MVs were extracted, separated by SDS-polyacrylamide gel electrophoresis and transferred onto a PVDF membrane. Next, denature and renature proteins on the membrane in AC buffer by gradually reducing the guanidine–HCl concentration. The PVDF membrane was then blocked with 5% milk for 1 hour and incubated with biotinylated BMSCs-MVs / ENPCs surface proteins overnight at 4 °C. Biotin-labeled proteins were detected using streptavidin-conjugated HRP. The corresponding bands in SDS-PAGE were excised for identification by mass spectrometry.

Co-immunoprecipitation assay

For the co-immunoprecipitation assay, 10 million cells were lysed on ice for 30 minutes in 1 ml of immunoprecipitation lysis buffer (P0013, Beyotime) containing protease and phosphatase inhibitor cocktails. Protein lysates were cleared by centrifugation at 14000 g for 20 min at 4°C. Supernatant was incubated with anti-FLAG or anti-HA antibody or anti-IgG antibody overnight at 4 °C with rotation gentle rotating. Then, 25 μL of Protein A/G Magnetic Beads (HY-K0202, MedChemExpress) were added and the mixture was incubated for an additional 4 hours. After washing five times with wash buffer, the bound proteins were eluted eluted by boiling in 1× SDS-PAGE loading buffer and analyzed by Western blot.

LC-MS/MS analysis

Mass spectrometry was performed at the Sangon Biotech (Shanghai) Co., Ltd. The target proteins were generated from magnetic beads or stained gel bands. Target protein bands were excised, enzymatically digested, redissolved in 0.1% formic acid, and loaded on a

ThermoFisher Q Exactive plus mass spectrometer (Thermo Fisher Scientific, USA). The mass spectrometer data were processed using Proteome Discoverer (v2.5, Thermo Fisher Scientific, USA) to identify proteins against the UniProt database. Protein-protein interaction network and gene functional enrichment analysis were performed using the STRING (https://www.string-db.org/) and GeneMANIA (https://genemania.org/) online website to identify candidate surface proteins on BMSCs-MVs and ENPCs.

qRT-PCR

Total RNA was extracted from cells and tissues with TRIzol reagent (R701, Vazyme) according to the manufacturer's protocol. The cDNA was synthesized with the HiScript III SuperMix (R323, Vazyme). qRT-PCR was performed using ChamQ Universal SYBR qPCR Master Mix (Q711, Vazyme) on a Roche LightCycler R480 system (Roche). The software GraphPad Prism v8.0 was used for data analysis. The primer sequences for qRT-PCR are listed in Supplemental Table 6.

RNA-sequencing analysis

Total RNA from colonic LMMP tissues (human and mouse) was quality-controlled (RIN > 7.0, Agilent Bioanalyzer 2100). Libraries were constructed via poly(A) enrichment, fragmentation, cDNA synthesis, and adapter ligation, followed by size selection (370–420 bp) and PCR amplification. Sequencing was performed on an Illumina NovaSeq platform (150 bp PE). After quality control (removal of adapters and low-quality reads), clean reads were aligned to the reference genome (GRCh38/GRCm39) using HISAT2 (v2.0.5). Gene expression was quantified by featureCounts (v1.5.0-p3), and differential gene expression between groups was identified with DESeq2 (v1.20.0), applying a threshold of $|\log_2 FC| \geq 1$ and adjusted $p < 0.05$. Functional annotation of differentially expressed genes was conducted through Gene Ontology (GO) and KEGG pathway analyses using clusterProfiler.

TEM

ENPCs of each group were collected and fixed with 2.5% phosphate buffered glutaraldehyde. After washing with PBS phosphate buffer (PH7.4) for 3 times, the samples were fixed with 1% osmic acid at the room temperature for 2 hour, gradually dehydrated with ethanol and acetone, then embedded and cut into 80-100 nm slices using an ultramicrotome, finally dyed with uranium-lead double staining, dried at room temperature overnight and observed by TEM (Hitachi).

References

1. Ahlqvist E, et al. Novel subgroups of adult-onset diabetes and their association with outcomes: a data-driven cluster analysis of six variables. *Lancet Diabetes Endocrinol.* 2018;6(5):361-369.
2. Ballou S, et al. Chronic Diarrhea and Constipation Are More Common in Depressed Individuals. *Clin Gastroenterol Hepatol.* 2019;17(13):2696-2703.
3. Schmidt EK, et al. SUnSET, a nonradioactive method to monitor protein synthesis. *Nat Methods.* 2009;6(4):275-277.
4. Xu L, et al. Protein quality control through endoplasmic reticulum-associated degradation maintains haematopoietic stem cell identity and niche interactions. *Nat Cell Biol.* 2020;22(10):1162-1169.
5. Meng Q, et al. Surfaceome analysis of extracellular vesicles from senescent cells uncovers uptake repressor DPP4. *Proc Natl Acad Sci U S A.* 2023;120(43):e2219801120.
6. Wu Y, et al. Detecting protein-protein interactions by Far western blotting. *Nat Protoc.*

2007;2(12):3278-3284.

Supplemental Figures

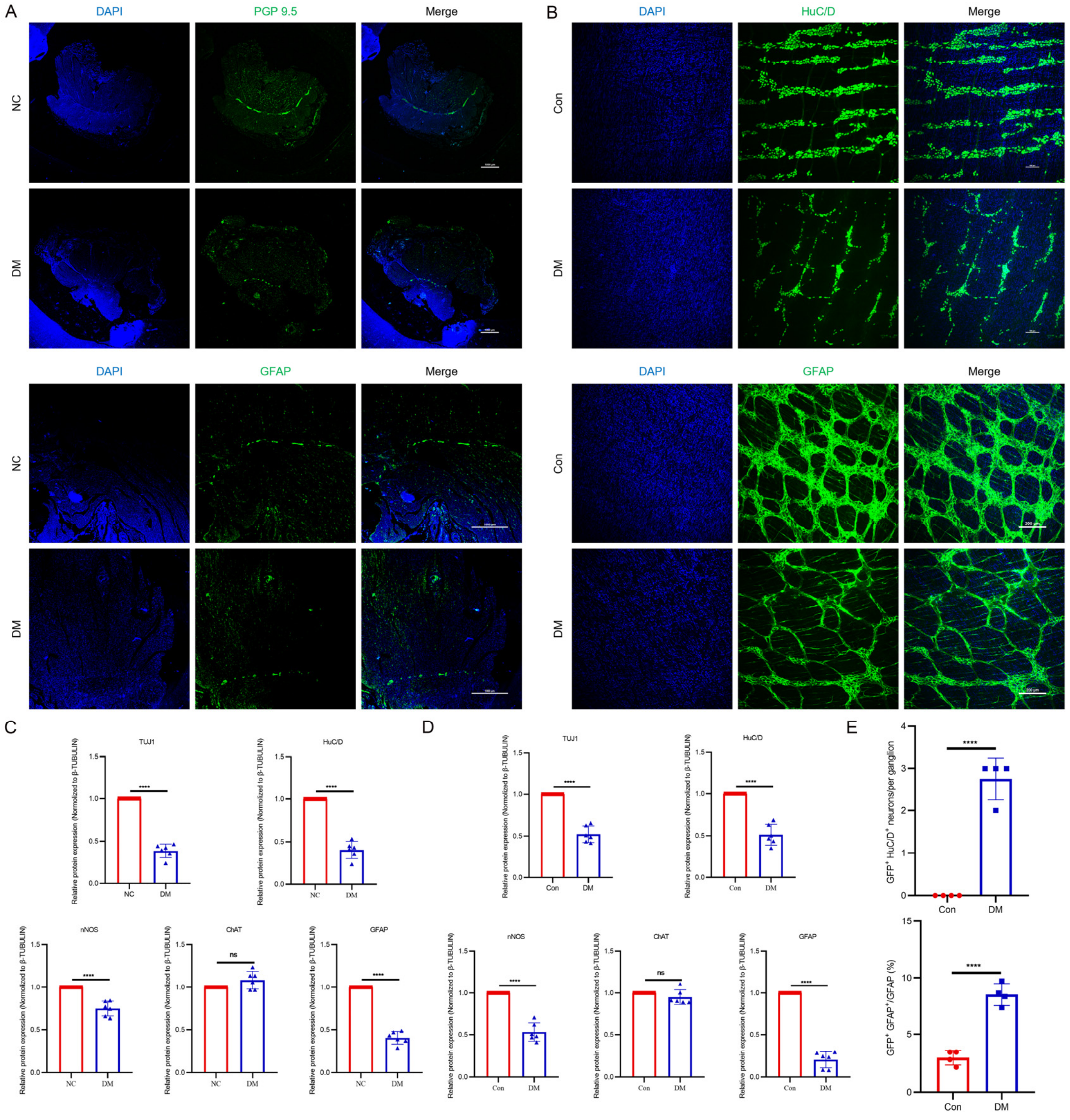

Supplemental Figure 1. Diabetic ENS injury in human and mouse colon. (A) Representative immunofluorescence images of myenteric neurons (PGP 9.5^{+}) and glial cells (GFAP^{+}) in human colon sections across groups. Scale bars: 1000 μm. **(B)** Representative immunofluorescence images of myenteric neurons (HuC/D^{+}) and glial cells (GFAP^{+}) in mouse colon sections across groups. Scale bars: 200 and 100 μm. **(C-D)** Quantification analysis of TUJ1, HuC/D, nNOS, ChAT, and GFAP protein expression in human **(C)** and mouse **(D)** colon tissues. n=6. **(E)** Quantification of GFP^{+} HuC/D^{+} neurons and GFP^{+} GFAP^{+} glial cells in colonic myenteric plexus across groups. n=4. Data are presented as mean ± SD. Statistical significance was determined by unpaired 2-tailed Student's t-test. ****$p < 0.0001$; ns, $p > 0.05$.

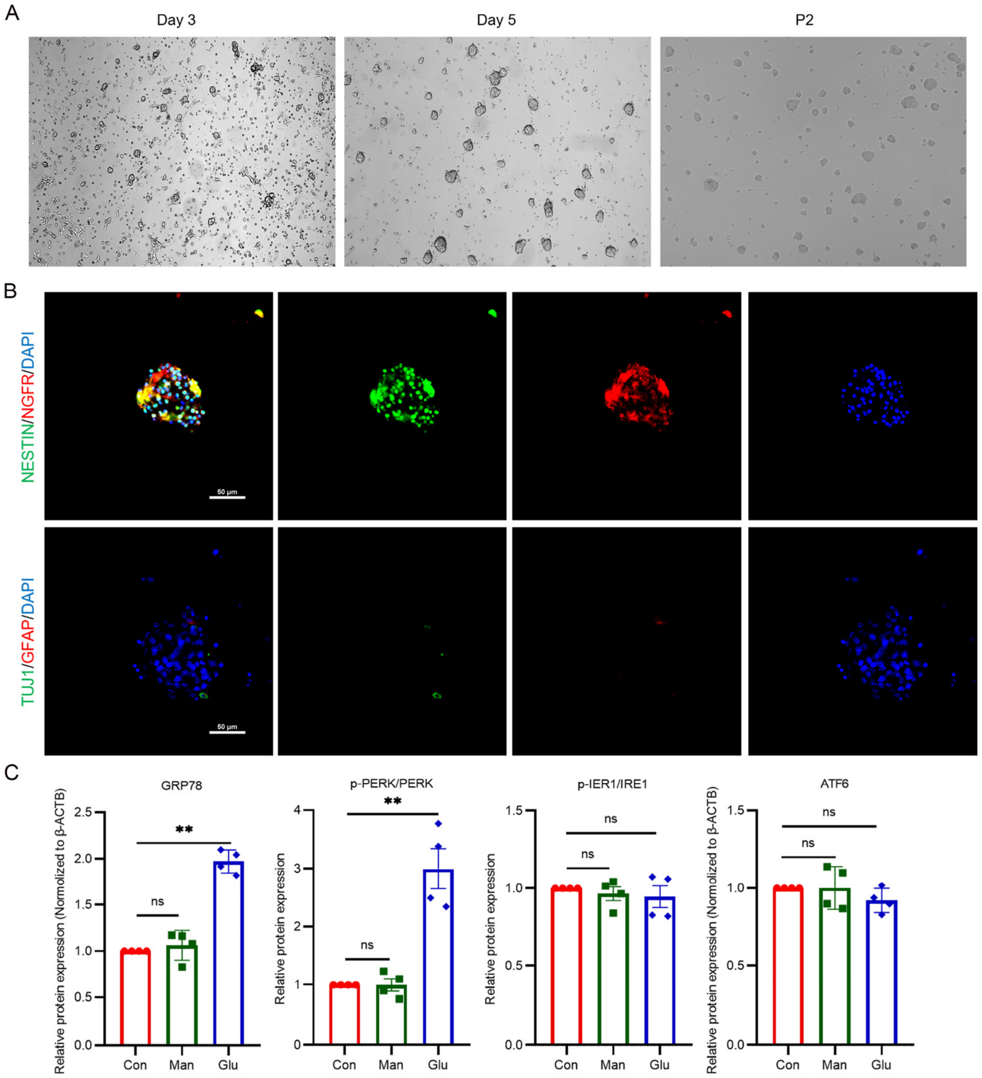

Supplemental Figure 2. Primary ENPCs characterization and ER stress/UPR activation under high glucose. (A-B) Morphology **(A)** and immunofluorescence **(B)** characterization of primary ENPCs neurospheres. Scale bars: 50 μm. **(C)** Quantification of ER stress/UPR markers (GRP78, p-PERK, PERK, p-IRE1α, IRE1α, ATF6) protein levels in ENPCs treated with high glucose or mannitol as osmotic control. n=4. Data are presented as mean ± SD. Statistical significance was determined by unpaired 2-tailed Student's t-test. *p <0.05, **p < 0.01; ns, p > 0.05.

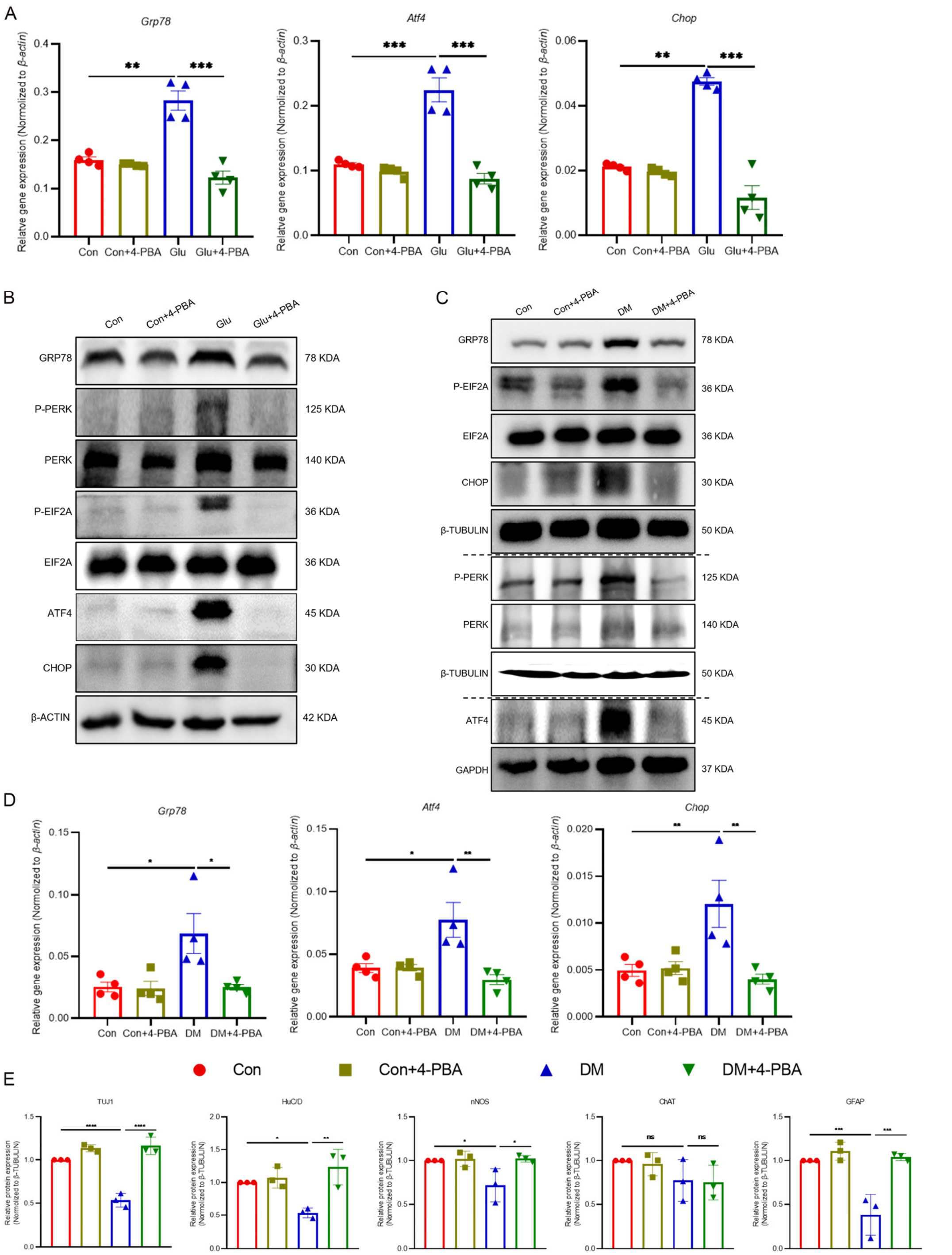

Supplemental Figure 3. 4-PBA treatment suppresses ER stress in vitro and in vivo. (A) The relative mRNA expression of *Grp78*, *Atf4*, and *Chop* in ENPCs treated with 4-PBA. n=4. **(B)** Western blot analysis of GRP78, p-PERK, PERK, p-EIF2A, EIF2A, ATF4, and CHOP in ENPCs treated with 4-PBA. **(C)** Western blot analysis of GRP78, p-PERK, PERK, p-EIF2A, EIF2A, ATF4, and CHOP in the colon in each group of mice treated with 4-PBA. **(D)** The relative mRNA expression of *Grp78*, *Atf4*, and *Chop* in the colon in each group of mice treated with 4-PBA. n=4. **(E)** Quantification of TUJ1, HuC/D, nNOS, ChAT and GFAP proteins expression in colon tissues from 4-PBA-treated mice. n=3. Data are presented as mean ± SD.

Statistical significance was determined by 1-way ANOVA with Tukey's multiple-comparison test. *$p < 0.05$, **$p < 0.01$, ***$p < 0.001$, ****$p < 0.0001$; ns, $p > 0.05$.

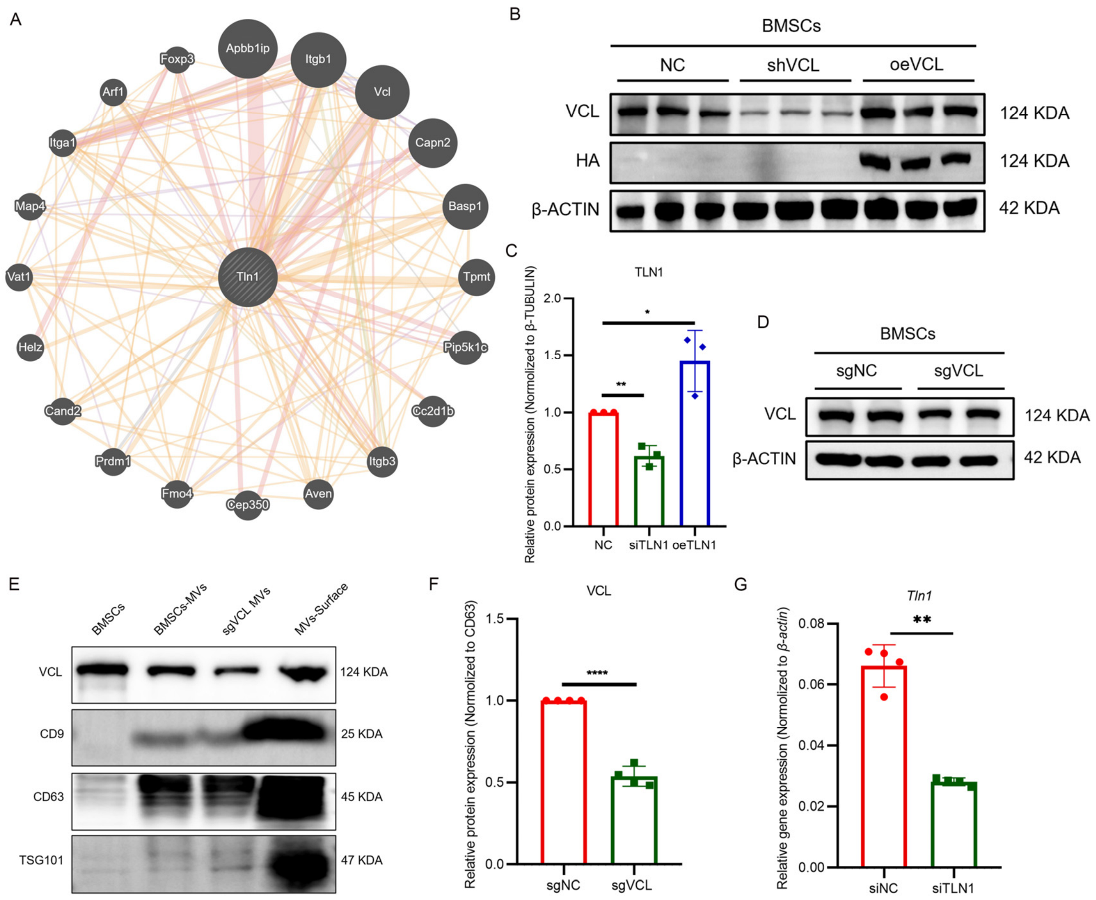

Supplemental Figure 4. Genetic validation of VCL/TLN1 expression in BMSCs and ENPCs. (**A**) Top 20 candidate proteins identified to interact with TLN1. **(B)** Western blot analysis of VCL and HA levels in BMSCs after *Vcl* knockdown (shVCL) or overexpression (oeVCL). **(C)** Qualification of TLN1 protein level in ENPCs after *Tln1* knockdown (siTLN1) or overexpression (oeTLN1). n=3. **(D)** Western blot analysis of VCL in BMSCs following sgRNA-mediated knockdown (sgVCL). **(E-F)** Western blot analysis **(E)** and qualification **(F)** of VCL in BMSCs-MVs following sgRNA-mediated knockdown (sgVCL). n=4. **(G)** Relative *Tln1* mRNA expression in ENPCs after siRNA treatment. Data are presented as mean ± SD. Statistical significance was determined by unpaired 2-tailed Student's t-test. **p* <0.05, ***p* < 0.01, *****p* < 0.0001.

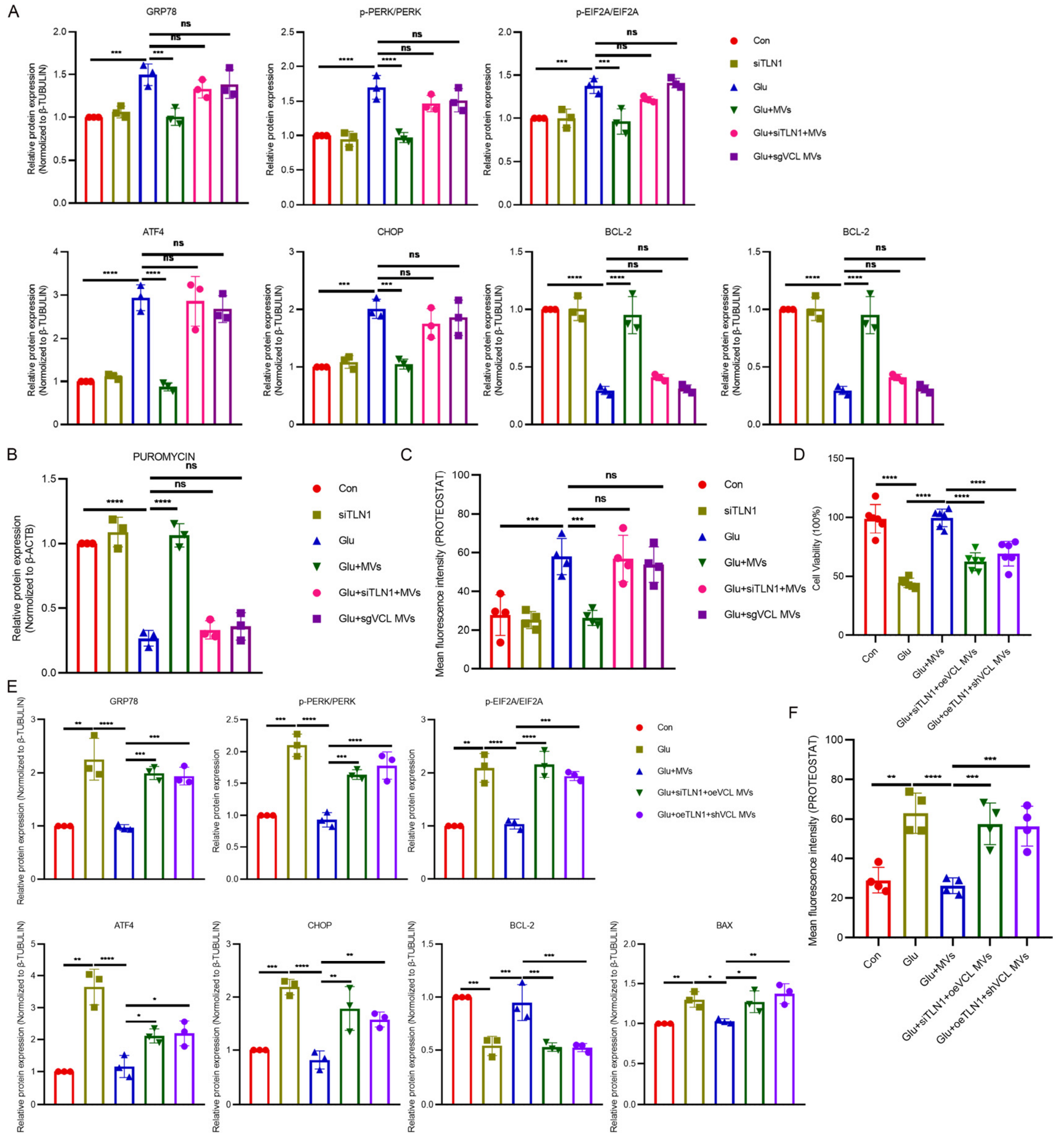

Supplemental Figure 5. Quantification of BMSCs-MVs–mediated recovery of ENPCs function and ER homeostasis through VCL–TLN1. (A) Quantification analysis of GRP78, p-PERK, PERK, p-EIF2α, EIF2α, ATF4, CHOP, BCL-2, and BAX proteins in ENPCs under different treatments. n=3. **(B)** Quantification of puromycin incorporation assessing global protein synthesis rates in ENPCs under different treatments. n=3. **(C)** Quantification of mean immunofluorescence intensity of PROTEOSTAT to detect protein aggregation in ENPCs under different treatments. n=4. **(D)** CCK-8 assay assessing ENPCs viability in rescue experiments. n=5. **(E)** Quantification of GRP78, p-PERK, PERK, p-EIF2α, EIF2α, ATF4, CHOP, BCL-2, and BAX protein levels in ENPCs in rescue experiments. n=3. **(F)** Quantification of PROTEOSTAT detecting protein aggregation in ENPCs. Scale bars: 10 μm. n=4. Data are presented as mean ± SD. Statistical significance was determined by 1-way ANOVA with Tukey's multiple-comparison test. **p* < 0.05, ***p* < 0.01, ****p* < 0.001, *****p* < 0.0001; ns, *p* > 0.05.

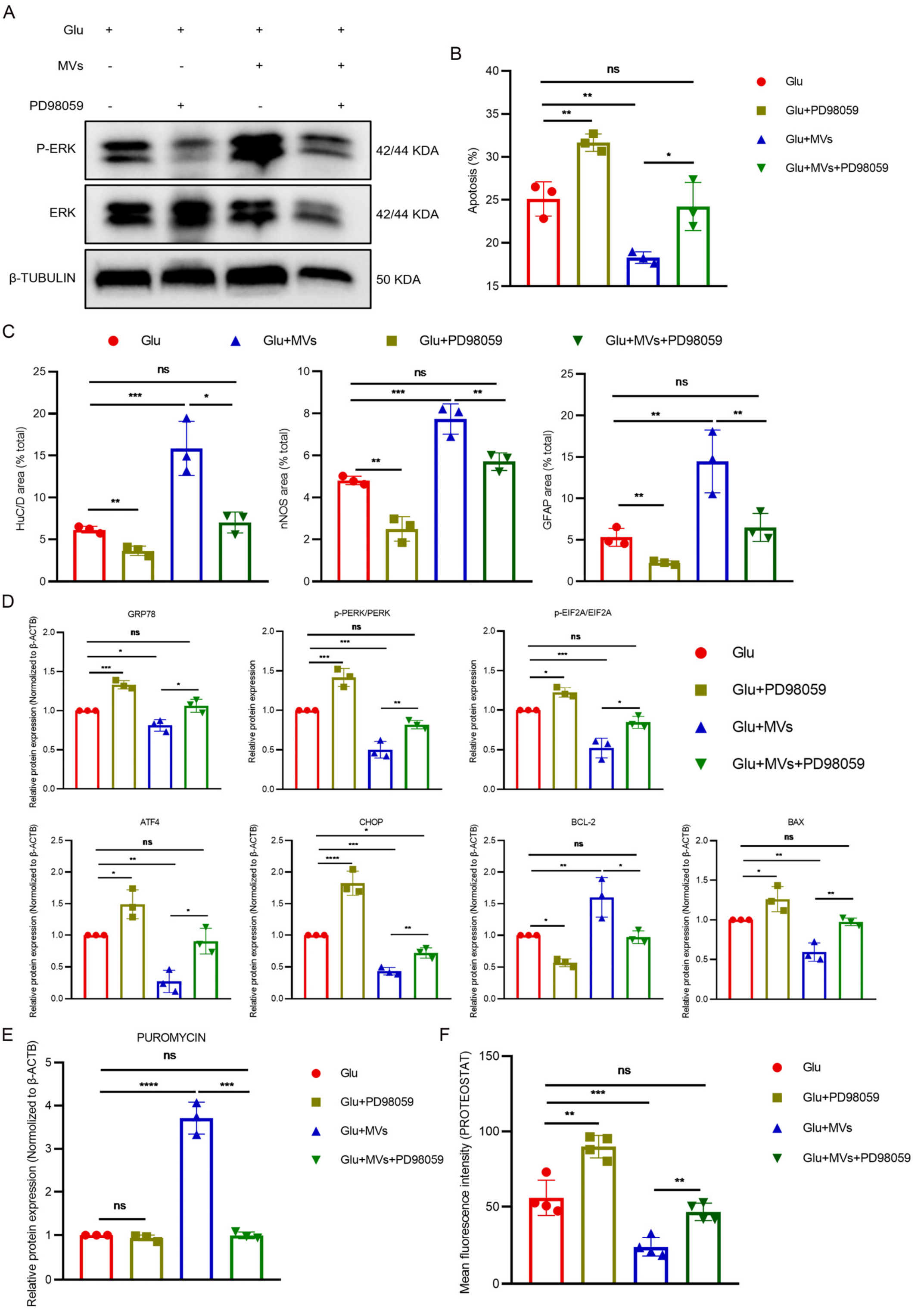

Supplemental Figure 6. Quantification of ERK-dependent protective effects induced by BMSCs-MVs in ENPCs. (A) Western blot of p-ERK and ERK in ENPCs treated with ERK inhibitor PD98059. in ENPCs treated with ERK inhibitor PD98059. n=3. **(B)** Quantification of total apoptotic rate in ENPCs treated with ERK inhibitor PD98059. n=3. **(C)** Quantification analysis of neuronal (HuC/D^{+}, nNOS^{+}) and glial (GFAP^{+}) differentiation in ENPCs treated with ERK inhibitor PD98059. n=3. **(D)** Quantification analysis of GRP78, p-PERK, PERK, p-

EIF2A, EIF2A, ATF4, CHOP, BCL-2, and BAX proteins in ENPCs treated with ERK inhibitor PD98059. n=3. **(E)** Quantification analysis of puromycin incorporation assessing global protein synthesis rates in ENPCs treated with ERK inhibitor PD98059. n=3. **(F)** Quantification of PROTEOSTAT detecting protein aggregation in ENPCs treated with ERK inhibitor PD98059. Scale bars: 10 μm. n=4. Data are presented as mean ± SD. Statistical significance was determined by 1-way ANOVA with Tukey's multiple-comparison test. *$p < 0.05$, **$p < 0.01$, ***$p < 0.001$, ****$p < 0.0001$; ns, $p > 0.05$.

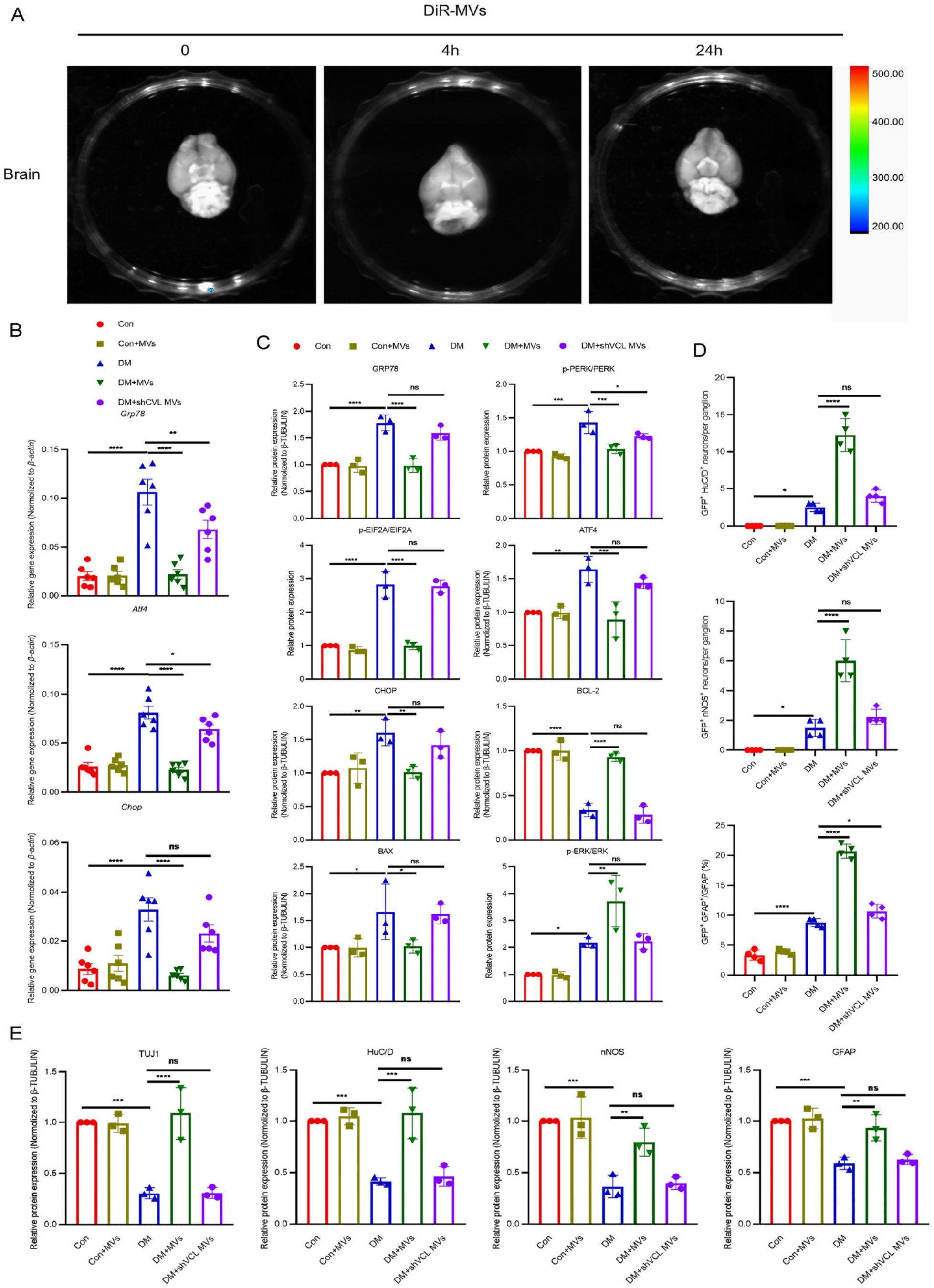

Supplemental Figure 7. Quantification of BMSCs-MVs therapeutic effects in diabetic mice. (A) Bioluminescence imaging of BMSCs-MVs distribution in the brain at 0, 4, and 24 hours post-injection. **(B)** Relative mRNA expression of *Grp78, Atf4*, and *Chop* in colon tissues from BMSCs-MVs treated mice. n=6. **(C)** Quantification analysis of GRP78, p-PERK, PERK, p-EIF2A, EIF2A, ATF4, CHOP, BCL-2, BAX, p-ERK and ERK proteins from BMSCs-MVs treated mice. n=3. (D) Quantification of ENPCs-derived neurons (HuC/D$^+$, nNOS$^+$), and GFAP$^+$ glial cells in colonic LMMP from BMSCs-MVs treated mice. n=4. **(E)** Quantification of TUJ1, HuC/D, nNOS, and GFAP protein levels in colon tissues from BMSCs-MVs treated mice. n=3. Data are presented as mean ± SD. Statistical significance was determined by 1-way ANOVA with Tukey's multiple-comparison test. **p* < 0.05, ***p* < 0.01, ****p* < 0.001, *****p* < 0.0001; ns, *p* > 0.05.

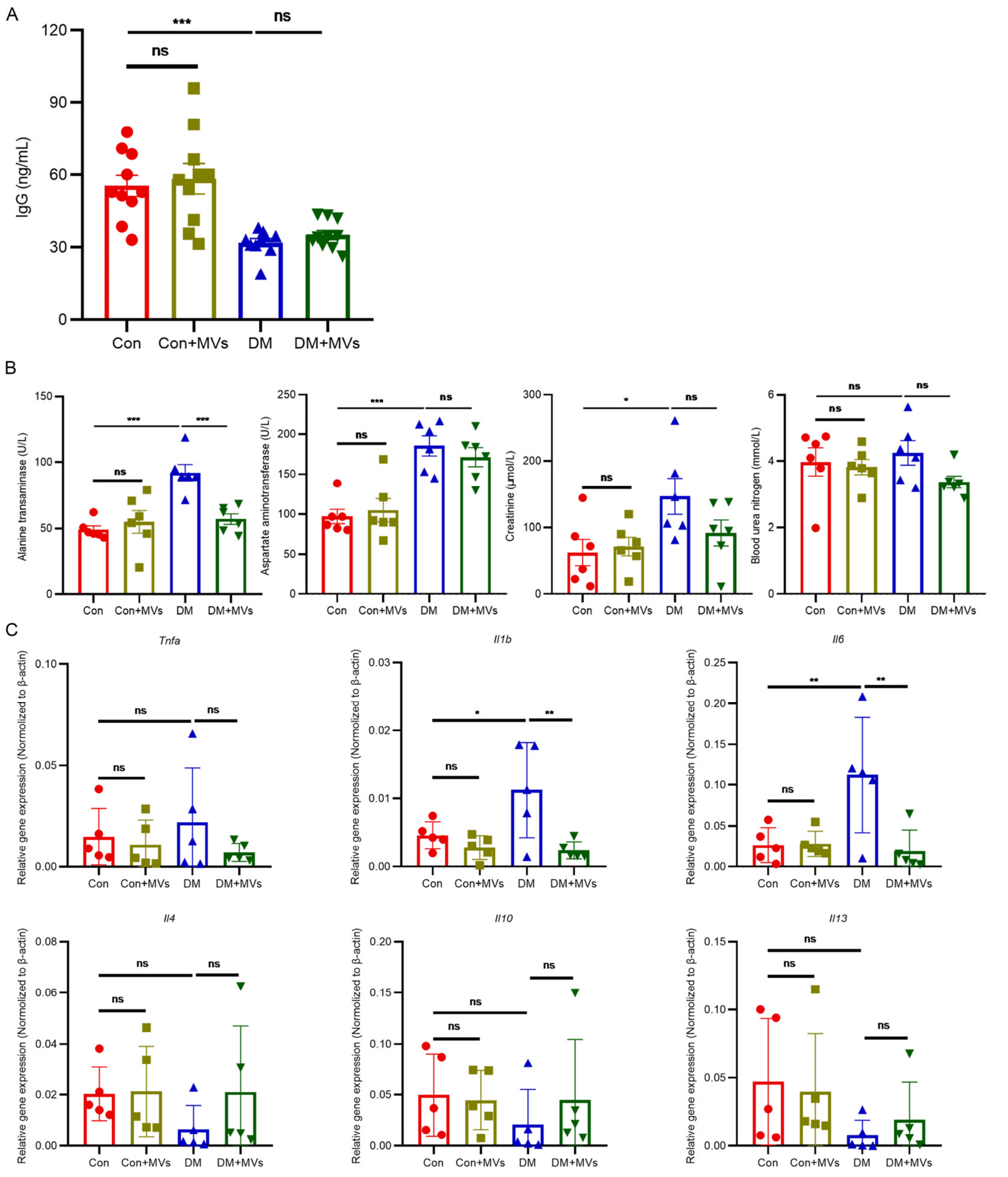

Supplemental Figure 8. Safety profile of BMSCs-MVs in vivo. (A) Serum IgG levels across experimental groups. n=10. **(B)** Serum biochemical markers of liver (alanine aminotransferase and aspartate aminotransferase) and kidney function (creatinine and blood urea nitrogen). n=6. **(C)** The relative mRNA expression of pro-inflammatory (*Tnfa, Il1b, Il6*) and anti-inflammatory (*Il4, Il10, Il13*) cytokines in colon tissues. n=5. Data are presented as mean ± SD. Statistical significance was determined by 1-way ANOVA with Tukey's multiple-comparison test. *$p < 0.05$, **$p < 0.01$, ***$p < 0.001$, ****$p < 0.0001$; ns, $p > 0.05$.

Supplemental Table 1. Mass spectrometry results of five bands identified from ENPCs by far Western blot assay

Gene_symbol	Unique Peptides	MW(KDA)
Tln1	83	269.7
Sptbn1	69	274.1
Eprs1	50	170
Lrp1	29	504.4
Cps1	26	164.5
Anpep	23	109.6
Numa1	15	235.5
Anxa1	13	38.7
Atp2b1	10	134.7
Iqgap1	8	188.6
Glg1	6	133.6
Vdac1	6	32.3
Slc25a5	6	32.9
Csk	5	50.7
Atp1b1	4	35.2
Map2k2	4	44.4
Map2k1	4	43.4
Coro1c	3	53.1

Supplemental Table 2. Overlapped mass spectrometry results identified from BMSCs-MVs by far Western blot and biotin pull-down assay

Gene_symbol	Unique Peptides	MW(KDA)
Adam9	1	93.6
Slc25a5	11	32.9
Atp1a1	46	112.9
Bsg	3	42.4
Ctnna1	22	100
Hk1	8	102.2
Cd47	6	35.3
Anxa1	17	38.7
Lpl	7	53.1
Slc3a2	23	58.3
Msn	3	67.7
P4hb	35	57
Enpp1	6	103
Slc2a1	5	53.9
Slc7a5	1	55.8
Stxbp3	3	67.9
Vcl	3	116.6
Adam10	2	83.9
Aldh3a2	1	53.9
Atp2b1	6	134.7
Bst2	1	19.1
Cavin1	1	43.9
Cd44	4	85.8
Cpne2	6	61
Epb41l3	2	103.3
Fyn	1	60.6

Krt1	4	65.6
Krt10	6	57
Lrp1	3	504.4
Mfge8	15	51.2
Nedd4	1	102.6
Panx1	1	48.1
Plxnb2	4	206.1
Prkca	5	76.8
Psen1	1	52.6
Sgcd	2	32.1
Slc1a5	7	58.4
Slc25a4	3	32.9
Slc38a1	6	53.8
Slc38a2	1	55.5
Slc39a10	2	94.3
Slc39a14	1	53.7
Slc6a6	4	69.8
Slc7a1	2	67
Slc7a2	3	71.7
Slc7a3	3	67.4
Sptbn1	2	274.1
Tmem119	1	29.4
Ttyh3	1	57.7
Vdac1	5	30.7

Supplemental Table 3. Baseline characteristics of the patient cohort with tissue sampling

	Normal Control	Diabetes	*P*
**Age (years, mean ± SD)**	64.87 ± 5.55	65.33 ± 8.79	0.8542
**Gender, *n* (%)**			>0.9999
Female	5 (33.33)	5 (33.33)	
Male	10 (66.67)	10 (66.67)	
**Body mass index (Kg/m^2, mean ± SD)**	24.27 ± 3.55	23.09 ± 2.86	0.3902
**Smoking, *n* (%)**	5 (33.33)	4 (26.67)	>0.9999
**Alcohol, *n* (%)**	4 (26.67)	3 (20.00)	>0.9999
**Surgery type**	Colectomy	Colectomy	-

Supplemental Table 4. Baseline characteristics of participants from the Endoscopy Center

	**Normal Control**	**Prediabetes**	**Diabetes**	***P***
**Age (years, mean ± SD)**	54.68 ± 11.89	51.11 ± 9.88	55.93 ± 11.42	0.3084
**Gender, *n* (%)**				0.4205
Female	85 (31.60)	11 (30.56)	17 (23.61)	
Male	184 (68.40)	25 (69.44)	55(76.39)	
**Body mass index (Kg/m^2, mean ± SD)**	21.70 ± 3.15	24.91 ± 3.51	24.78 ± 4.14	0.0457

Supplemental Table 5. The primary and secondary antibodies used for Western blot and immunofluorescence

Primary antibody	Producer (Catalog Number)	Concentration
Rabbit anti-TUJ1	Abclonal (A17913)	IF (1:200); WB (1:1000)
Rabbit anti-HuC/D	Abcam (ab184267)	IF (1:500); WB (1:1000)
Rabbit anti-nNOS	Genetex (GTX133403)	IF (1:200); WB (1:1000)
Rabbit anti-ChAT	Boster (A01192)	WB (1:1000)
Rabbit anti-GFAP	Abclonal (A0237)	IF (1:200); WB (1:1000)
Chicken anti-GFAP	Genetex (GTX85454)	IF (1:200)
Rabbit anti-NESTIN	Abclonal (A11861)	IF (1:200)
Rat anti-NGFR	Abcam (ab271289)	IF (1:500)
Mouse anti-PGP 9.5	Abcam (ab8189)	IF (1:200)
Goat anti-GFP	Abcam (ab5450)	IF (1:1000)
Rabbit anti-GRP78	Proteintech (11587-1-AP)	IF (1:200); WB (1:1000)
Rabbit anti-p-PERK	Affinity (DF7576)	WB (1:500)
Rabbit anti-PERK	Proteintech (20582-1-AP)	WB (1:500)
Rabbit anti-p-EIF2A	Proteintech (28740-1-AP)	WB (1:1000)
Rabbit anti-EIF2A	Proteintech (11170-1-AP)	WB (1:1000)
Rabbit anti-ATF4	Proteintech (10835-1-AP)	WB (1:1000)
Rabbit anti-p-IRE1	Affinity (AF7150)	WB (1:500)
Rabbit anti-IRE1	Affinity (DF7709)	WB (1:500)
Rabbit anti-ATF6	Proteintech (24169-1-AP)	WB (1:1000)
Rabbit anti-CHOP	Proteintech (15204-1-AP)	WB (1:1000)
Rabbit anti-BCL-2	Proteintech (26593-1-AP)	WB (1:2000)
Rabbit anti-BAX	Proteintech (50599-2-Ig)	WB (1:5000)
Rabbit anti-p-ERK (Thr202/Tyr204)	CST (4370)	WB (1:1000)
Rabbit anti-ERK	CST (4696)	WB (1:1000)
Rabbit anti-p-P38 (Tyr182)	Genetex (GTX639570)	WB (1:1000)
Rabbit anti-P38 (Tyr182)	Genetex (GTX635797)	WB (1:1000)
Rabbit anti-p-JNK (Thr183/Tyr185)	Genetex (GTX24821)	WB (1:1000)
Rabbit anti-JNK	Genetex (GTX52360)	WB (1:1000)
Rabbit anti-TSG101	Proteintech (28283-1-AP)	WB (1:1000)
Rabbit anti-CD9	Abclonal (A19027)	WB (1:1000)
Rabbit anti-CD63	Abcam (ab217345)	WB (1:1000)
Rabbit anti-CALNEXIN	Servicebio (GB115471)	WB (1:1000)

Rabbit anti-PUROMYCIN	Abclonal (A23031)	WB (1:3000)
Rabbit anti-VCL	Abclonal (A2752)	WB (1:50000)
Rabbit anti-TLN1	Abcam (ab108480)	WB (1:3000)
Mouse anti-FLAG	Sigma (F1804)	Immunoprecipitation (1:200)
Rabbit anti-FLAG	Proteintech (20543-1-AP)	WB (1:20000)
Rabbit anti-HA	Abclonal (AE105)	Immunoprecipitation (1:200); WB (1:20000)
Mouse anti-IgG	Beyotime (A7028)	Immunoprecipitation (1:200)
Rabbit anti-IgG	Beyotime (A7016)	Immunoprecipitation (1:200)
Rabbit anti-β-TUBULIN	Beyotime (AF1216)	WB (1:3000)
Rabbit anti-β-ACTIN	Abclonal (AC026)	WB (1:100000)
Rabbit anti-GAPDH	Beyotime (AF1186)	WB (1:3000)
**Secondary antibody**		
HRP-labelled anti-rabbit antibody	AntGene (ANT016)	WB (1:3000)
HRP-labelled anti-streptavidin antibody	Bioss (bs-0437P-HRP)	WB (1:3000)
Alexa Fluor 488 and donkey anti-goat IgG	AntGene (ANT025)	IF (1:200)
Alexa Fluor 488 and donkey anti-rabbit IgG	AntGene (ANT024)	IF (1:200)
Alexa Fluor 488 and donkey anti-mouse IgG	AntGene (ANT023)	IF (1:200)
Alexa Fluor 594 and donkey anti-rabbit IgG	AntGene (ANT030)	IF (1:200)
Alexa Fluor 594 and donkey anti-goat IgG	AntGene (ANT031)	IF (1:200)
Goat Anti Chicken IgY(H+L) (AbFluor 594)	Immunoway (RS3601)	IF (1:200)
Alexa Fluor 647 and donkey anti-rat IgG	AntGene (ANT037S)	IF (1:200)

Supplemental Table 6. Primers used for qRT-PCR

Gene	Forward primer (5′-3′)	Reverse primer (5′-3′)
Grp78	GACTGCTGAGGCGTATTTGG	AGCATCTTTGGTTGCTTGTCG
Atf4	CCTGAACAGCGAAGTGTTGG	TGGAGAACCCATGAGGTTTCAA
Xbp1s	AGCTTTTACGGGAGAAAACTCAC	CCTCTGGAACCTCGTCAGGA
Atf6	GTCCAAAGCGAAGAGCTGTCTG	AGAGATGCCTCCTCTGATTGGC
Chop	AAGCCTGGTATGAGGATCTGC	TTCCTGGGGATGAGATATAGGTG
Tln1	AGTGACGGACAGCATCAACCAG	GGATTCTCCAGGAGTTCTCGGA
Tnfa	GGTGCCTATGTCTCAGCCTCTT	GCCATAGAACTGATGAGAGGGAG
Il1b	TGGACCTTCCAGGATGAGGACA	GTTCATCTCGGAGCCTGTAGTG
Il6	TACCACTTCACAAGTCGGAGGC	CTGCAAGTGCATCATCGTTGTTC
Il4	ATCATCGGCATTTTGAACGAGGTC	ACCTTGGAAGCCCTACAGACGA
Il10	CGGGAAGACAATAACTGCACCC	CGGTTAGCAGTATGTTGTCCAGC
Il13	AACGGCAGCATGGTATGGAGTG	TGGGTCCTGTAGATGGCATTGC
β-Actin	GGCTGTATTCCCCTCCATCG	CCAGTTGGTAACAATGCCATGT
