# Supplementary material for: BM-derived mesenchymal stem cell microvesicles protect enteric neural precursor cells and alleviate diabetes-associated enteric neuropathy
Source: J Clin Invest. 2026 Mar 16;136(6):e192437. doi: 10.1172/JCI192437 (PMC12987631; doi:10.1172/JCI192437)

Full unedited blot for Figure 1D

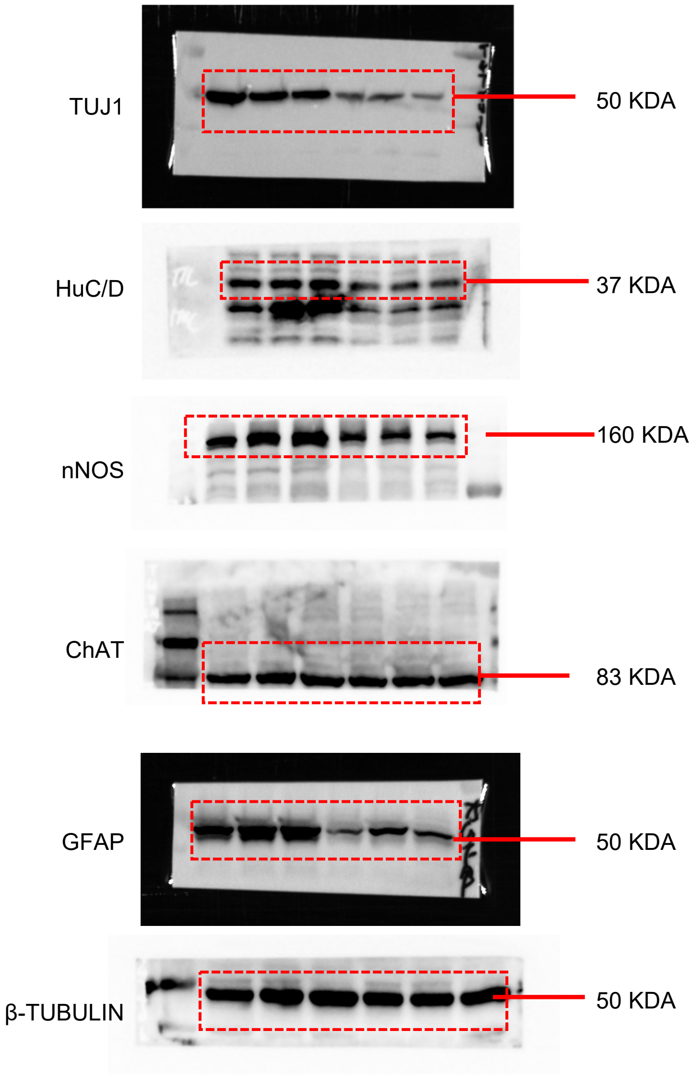

Full unedited blot for Figure 1E

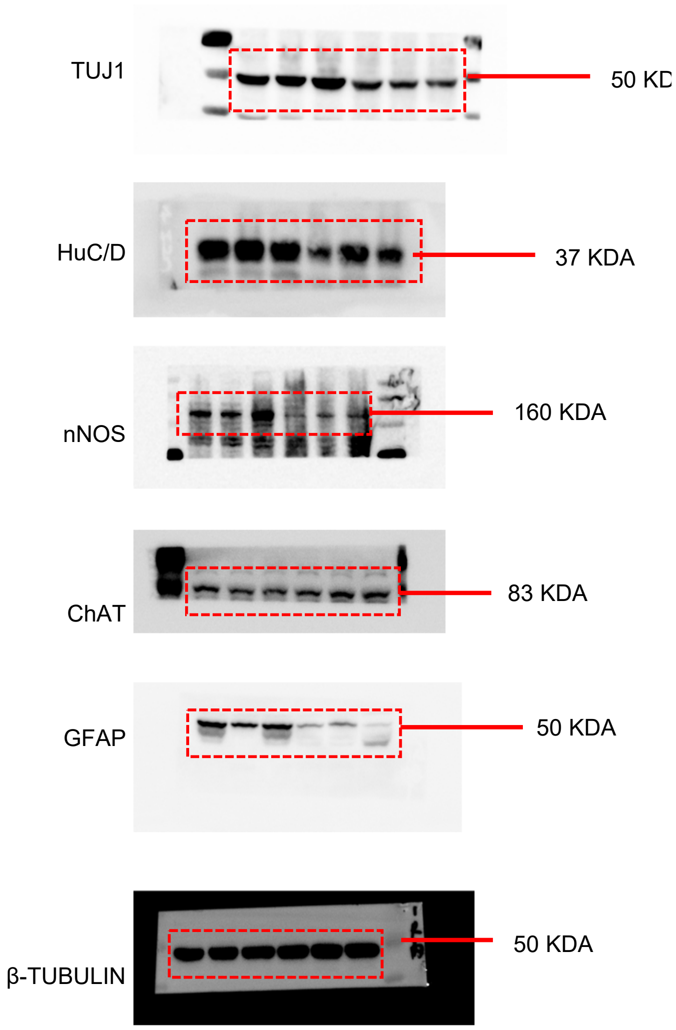

Full unedited blot for Figure 2C

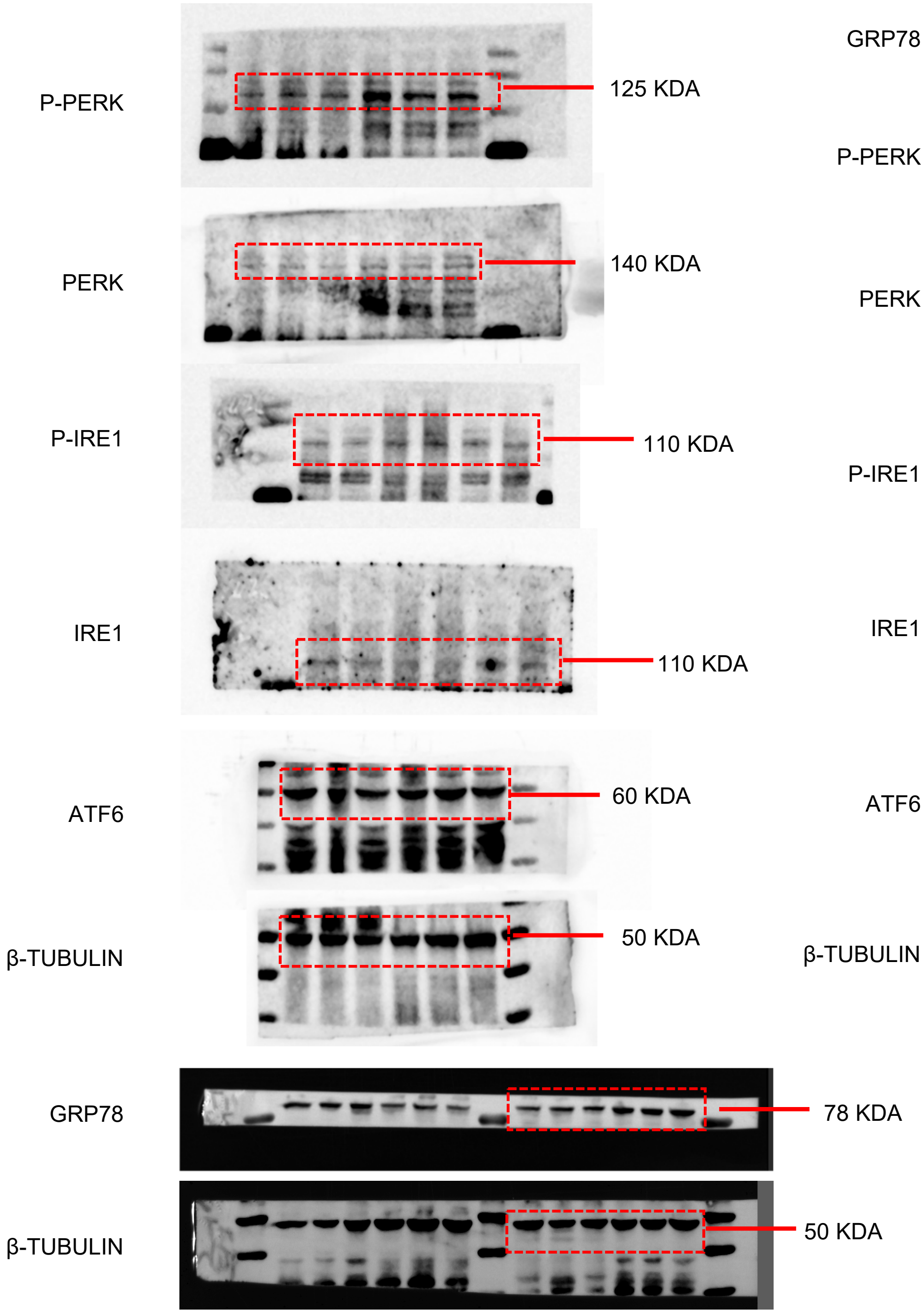

Full unedited blot for Figure 2D

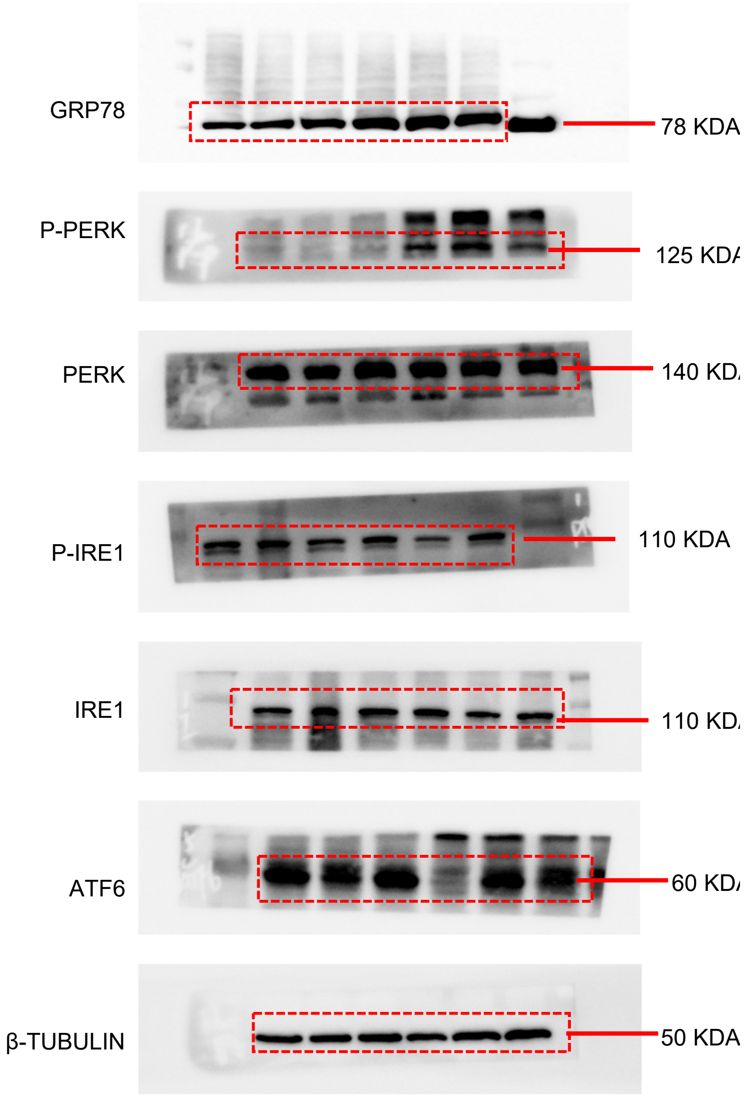

In Figure 2C, six tissue samples (control, and DM groups) were run in parallel gel on the same day for the GRP78 detection, and the loading control for it is shown below the blot.

Full unedited blot for Figure 2G

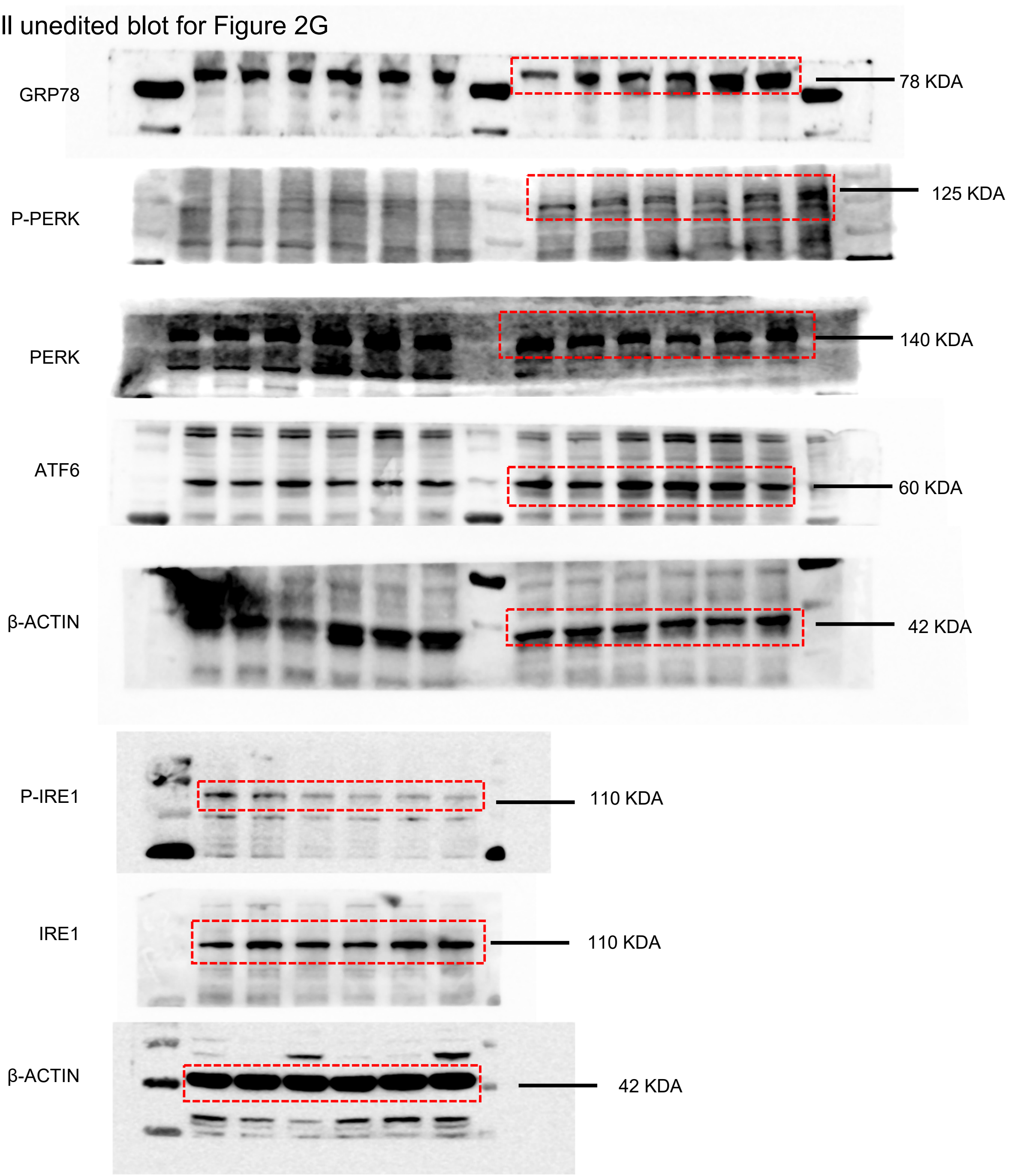

In Figure 2G, six ENPCs samples (control, mannitol and high glucose groups) were run in parallel gel on the same day for the P-IRE1 and IRE1 detection, and the loading control for it is shown below the blot.

Full unedited blot for Figure 4E

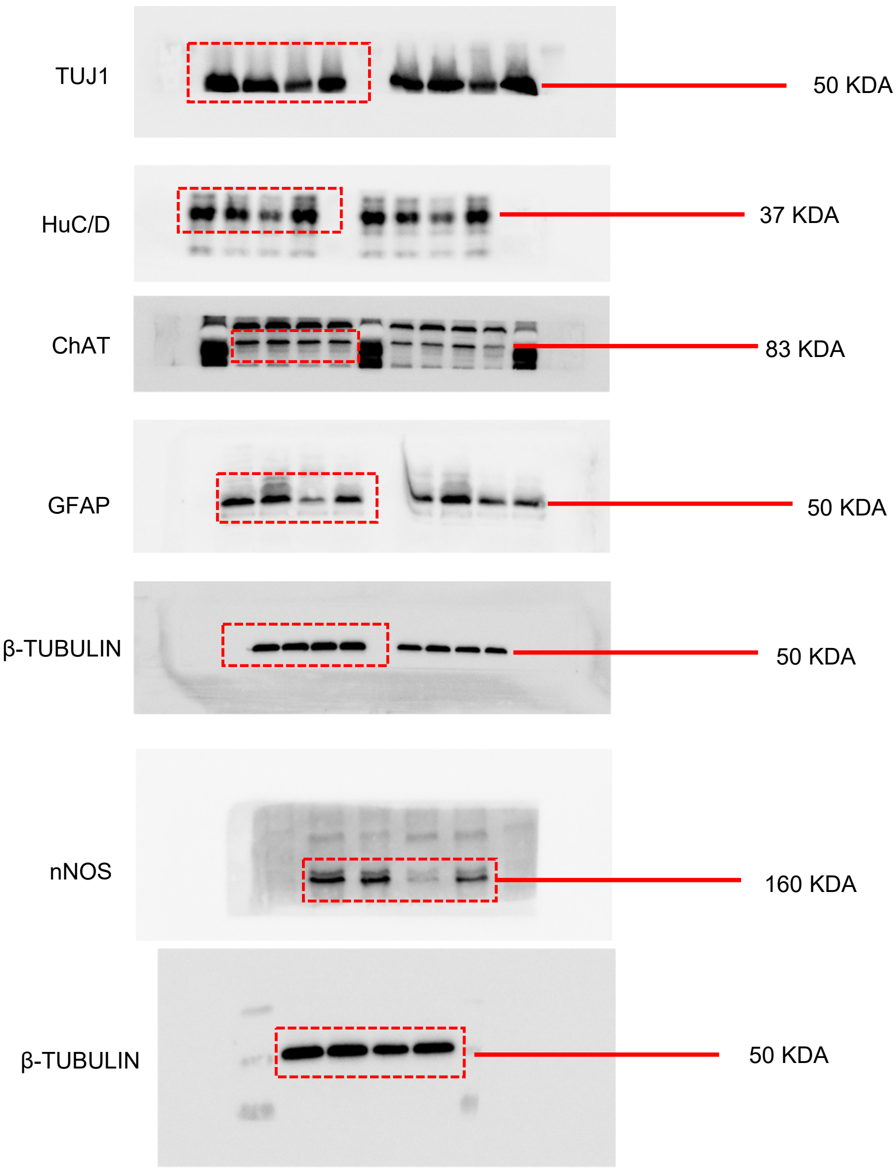

Full unedited blot for Figure 5C

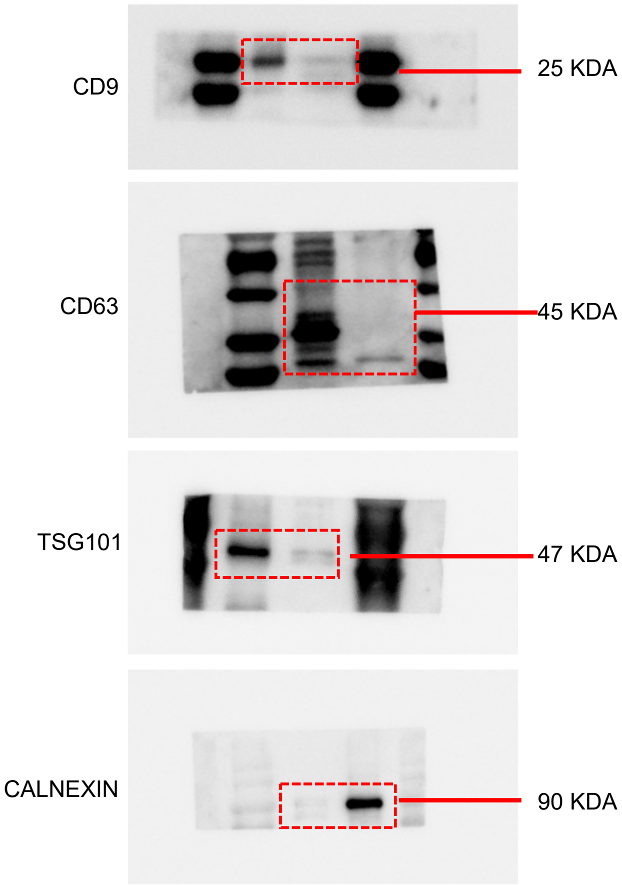

In Figure 4E, four tissue samples (Con, Con+4-PBA, DM, DM+4-PBA) were run in parallel gel on the same day for the nNOS detection, and the loading control for it is shown below the blot.

Full unedited blot for Figure 5D

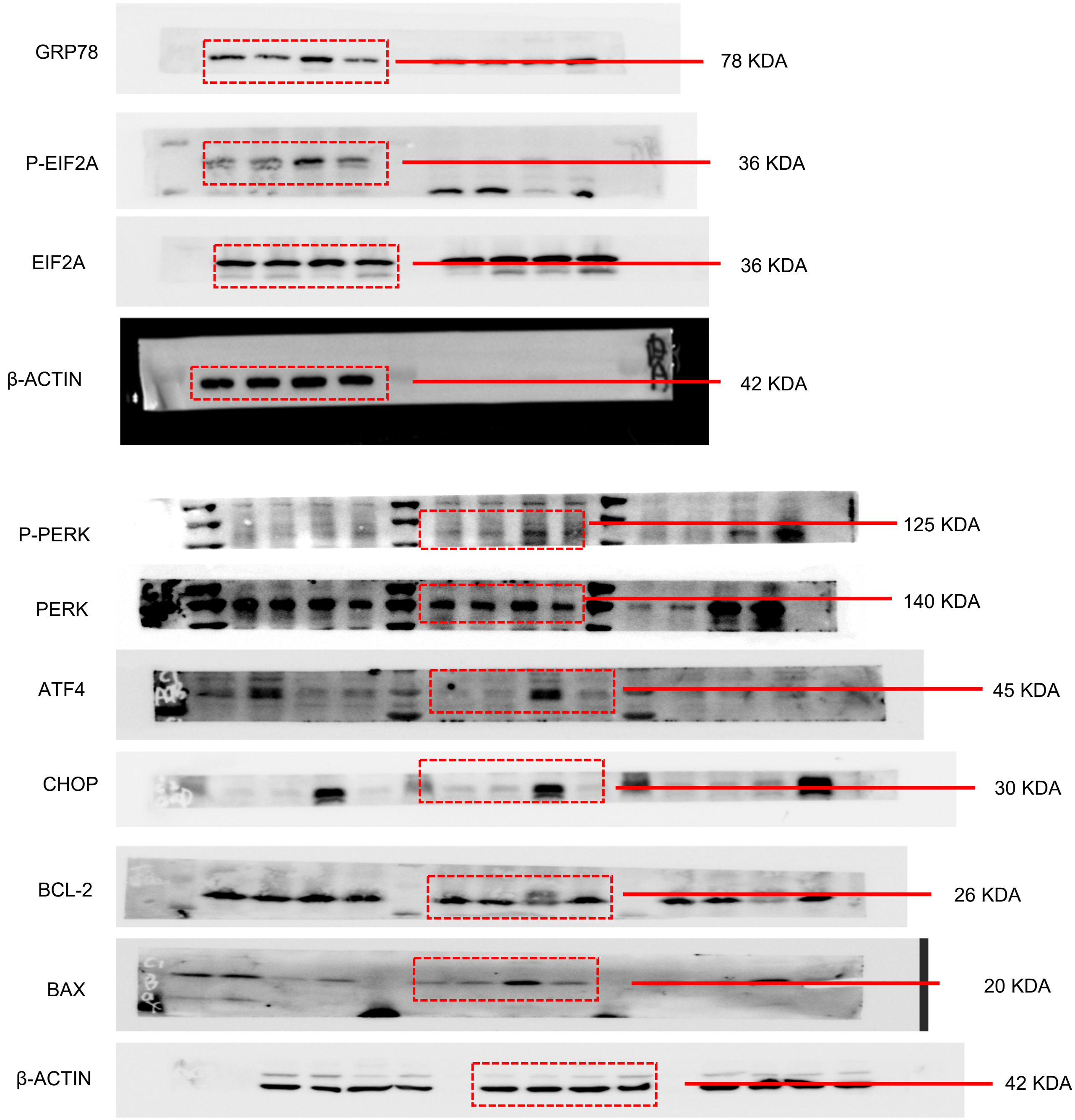

In Figure 5D, four tissue samples (Con, Con+MVs, Glu, Glu+MVs) were run in parallel gel on the same day for the GRP78, P-EIF2A, and EIF2A detection, and the loading control for it is shown below the blot.

Full unedited blot for Figure 5F

PUROMYCIN

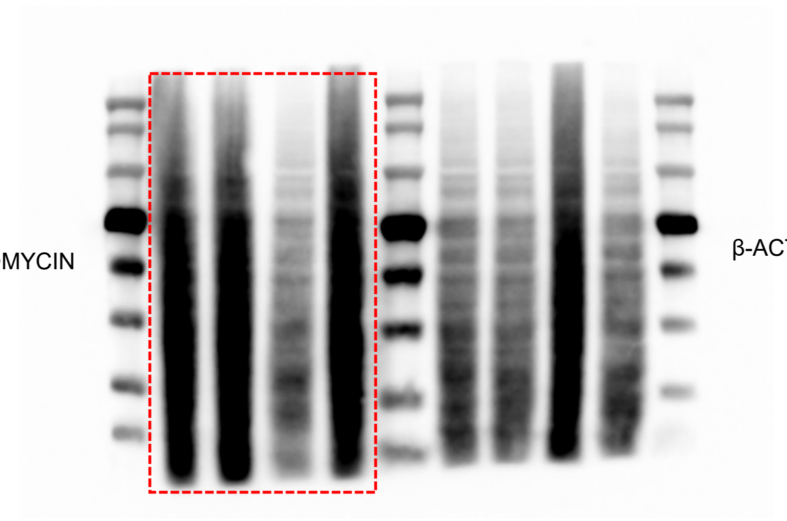

β-ACTIN

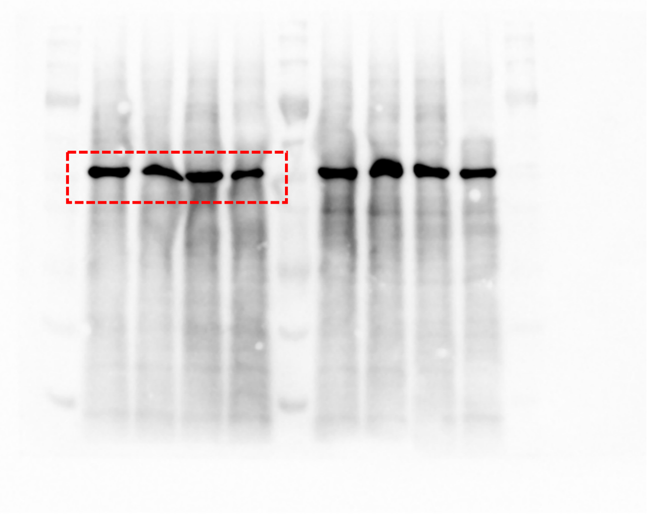

Full unedited blot for Figure 8A

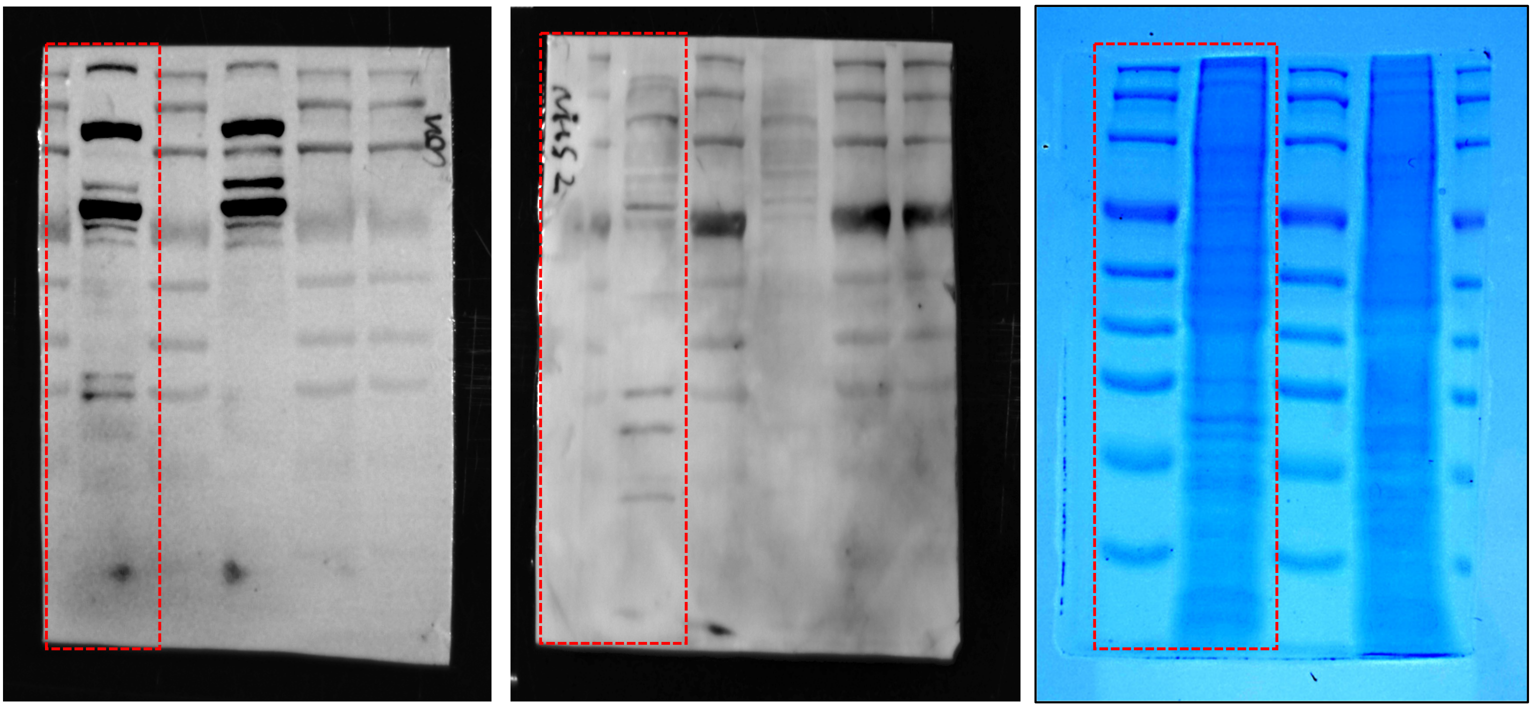

Full unedited blot for Figure 8B

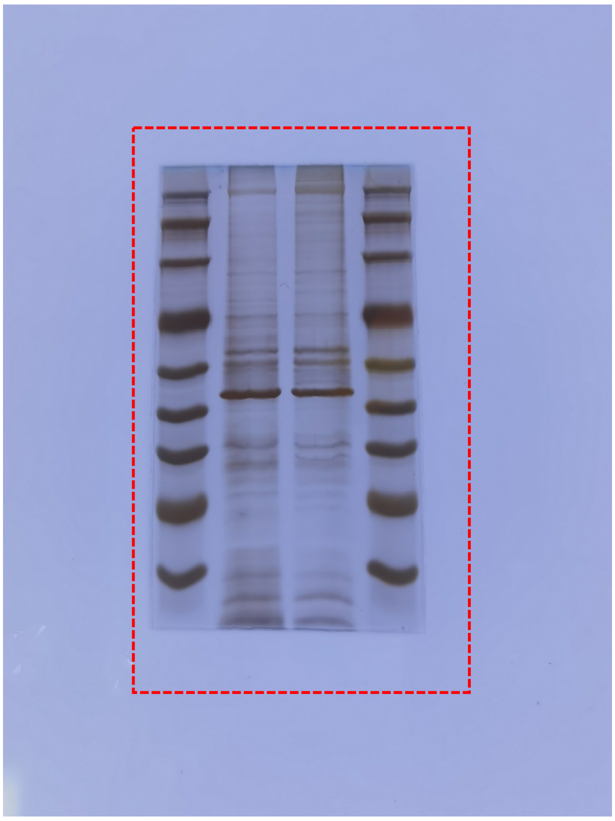

Full unedited blot for Figure 8D

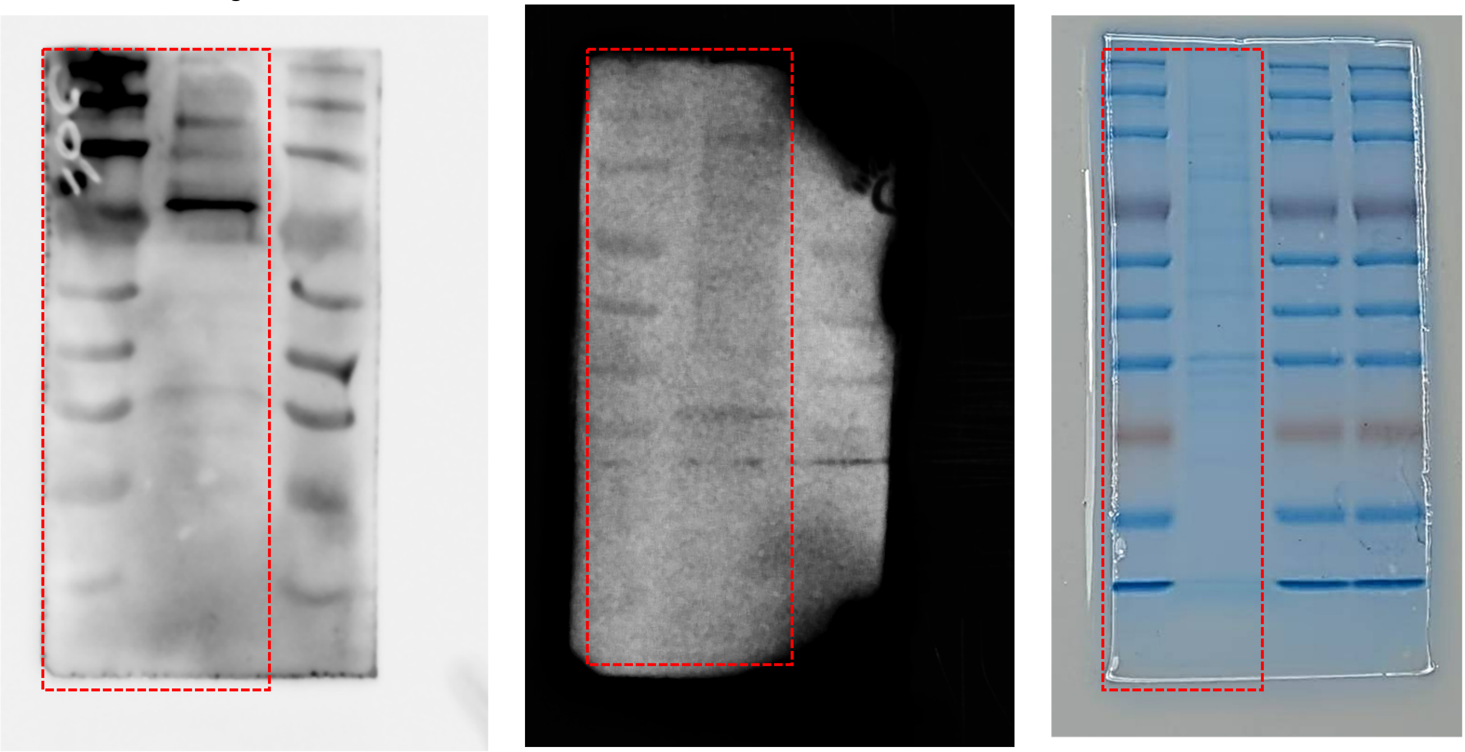

Full unedited blot for Figure 8E

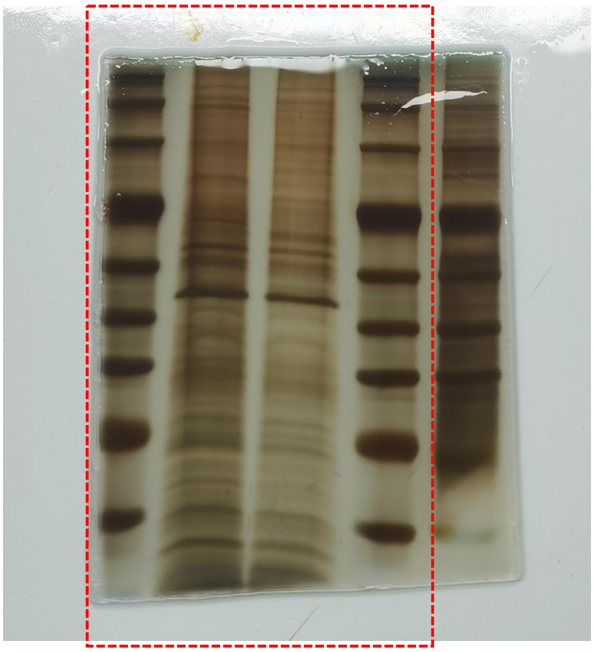

Full unedited blot for Figure 8G

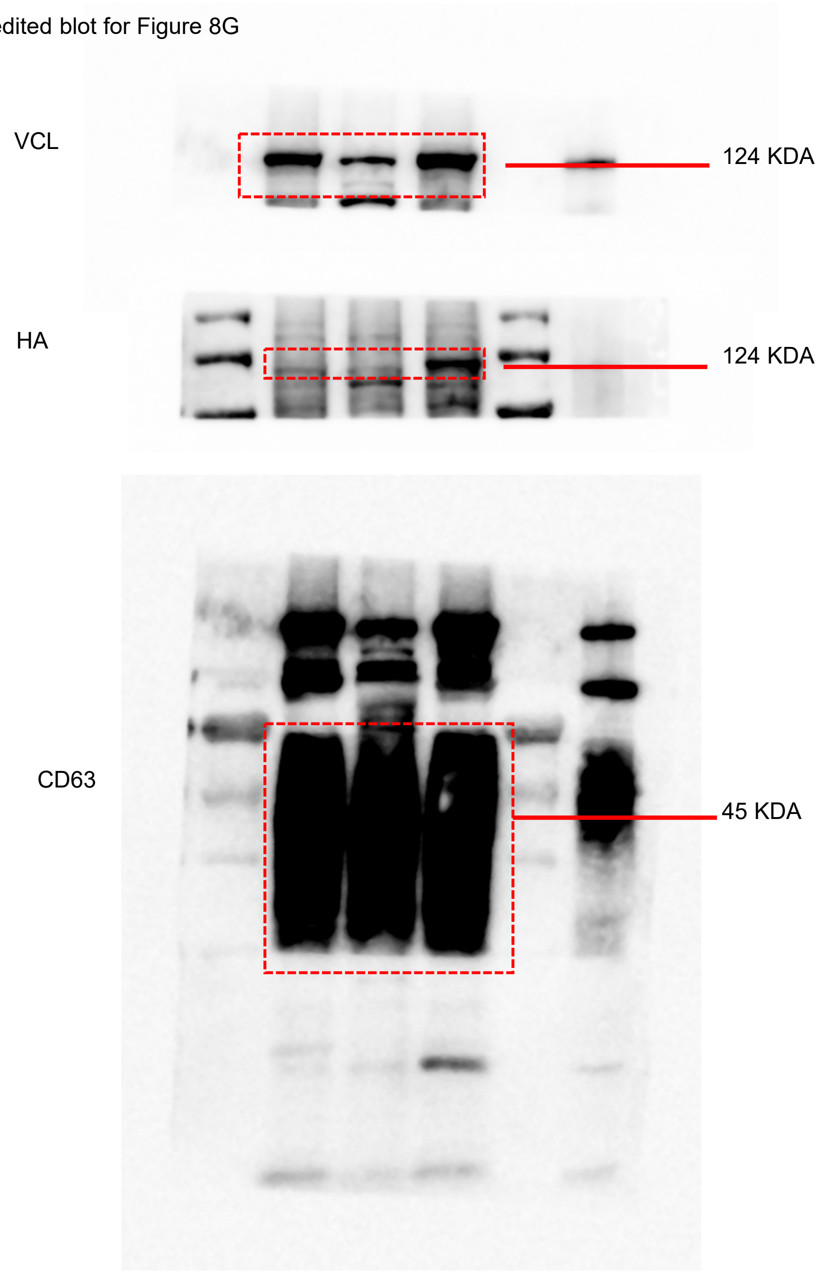

Full unedited blot for Figure 8H

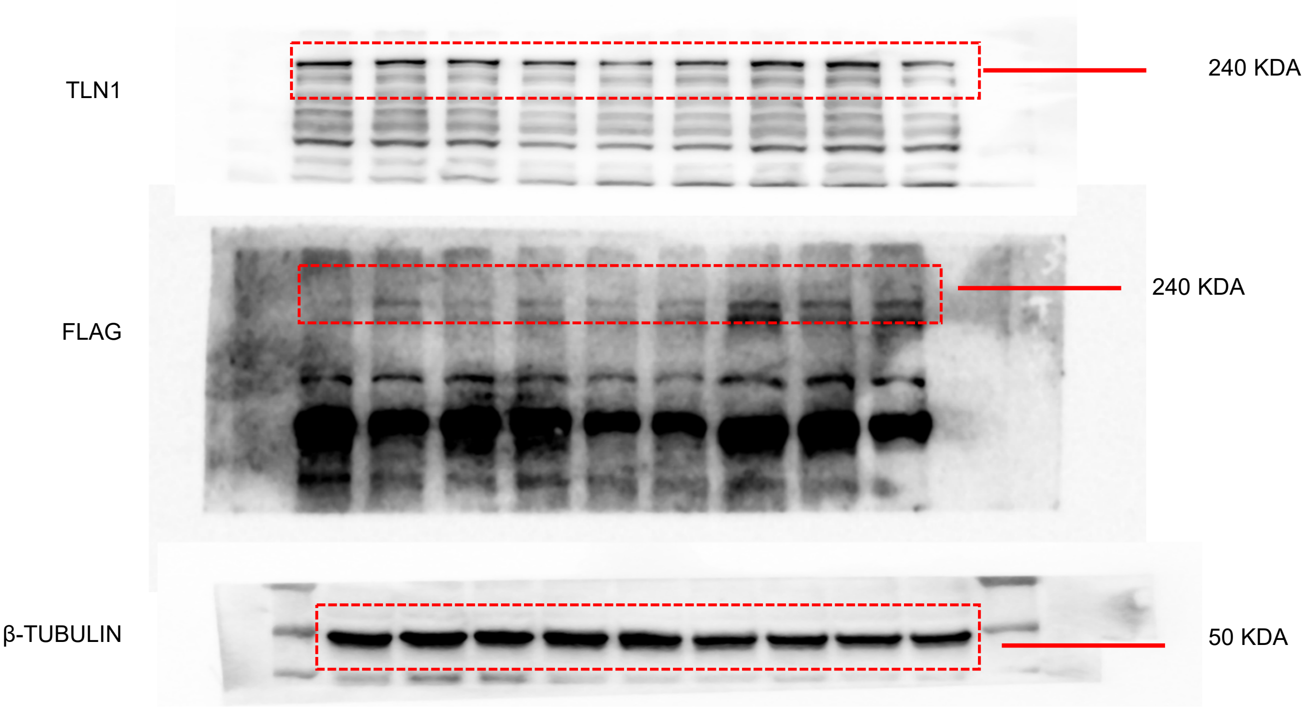

Full unedited blot for Figure 8I

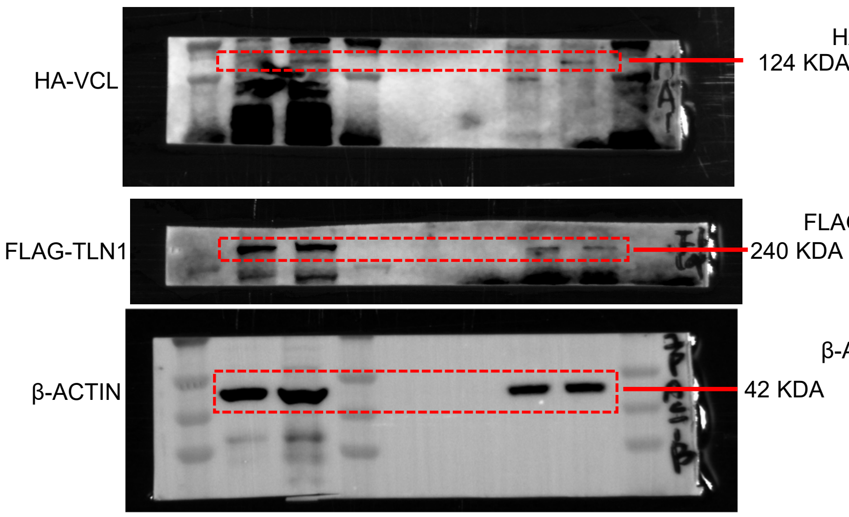

Full unedited blot for Figure 8J

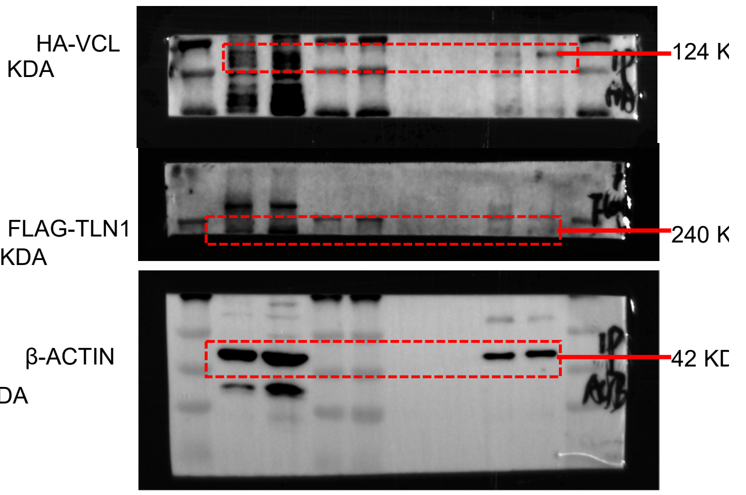

Full unedited blot for Figure 9H

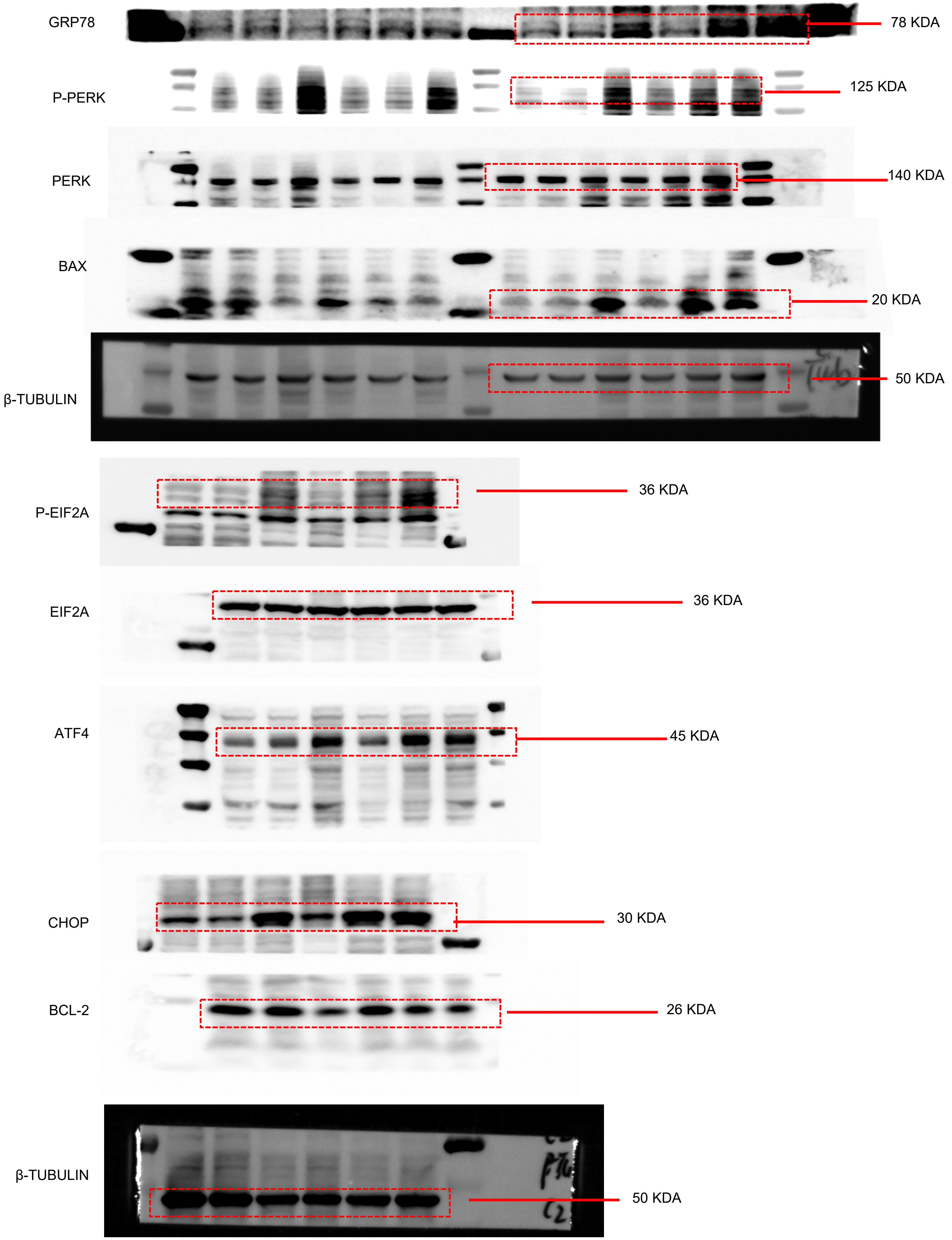

In Figure 9H, six cell samples were run in parallel gel on the same day for GRP78, P-PERK, PERK, and BAX detection, and the loading control for it is shown below the blot.

Full unedited blot for Figure 9I

PUROMYCIN

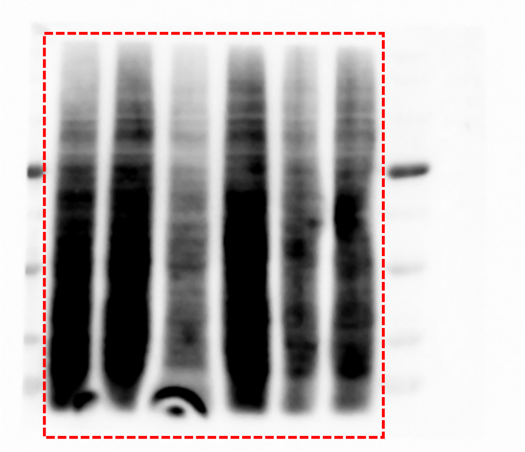

β-ACTIN

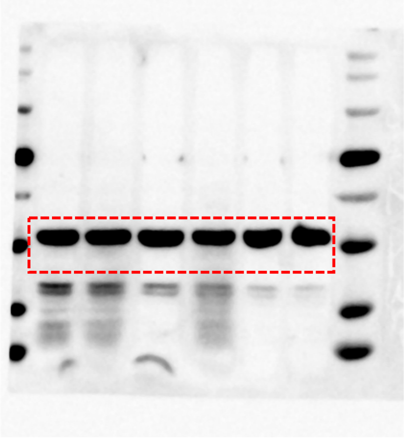

Full unedited blot for Figure 10E

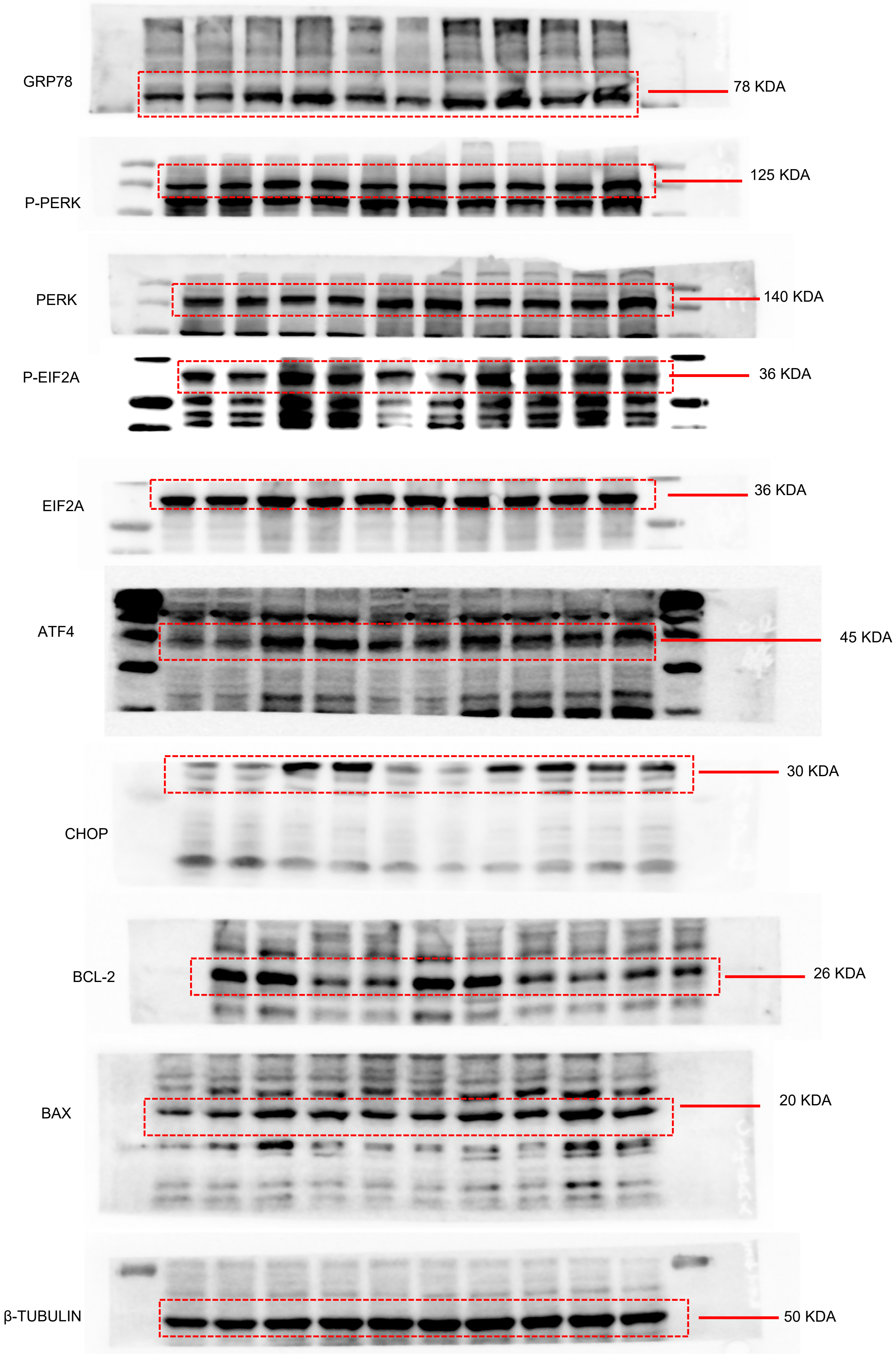

Full unedited blot for Figure 10F

PUROMYCIN

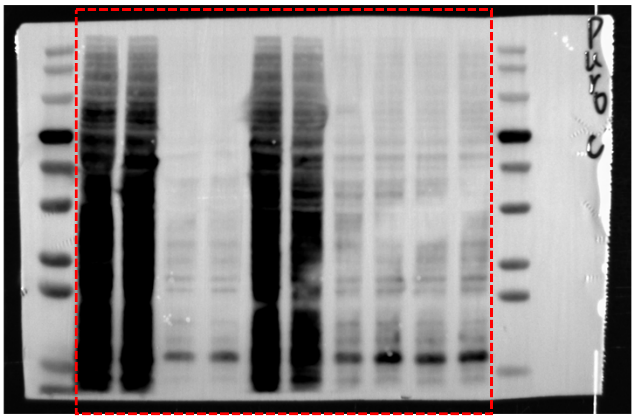

β-ACTIN

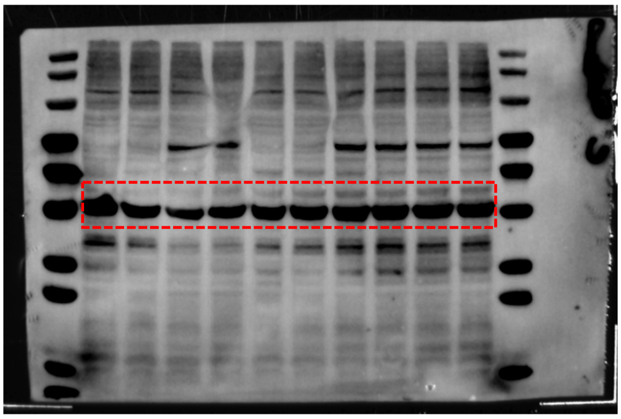

Full unedited blot for Figure 11D

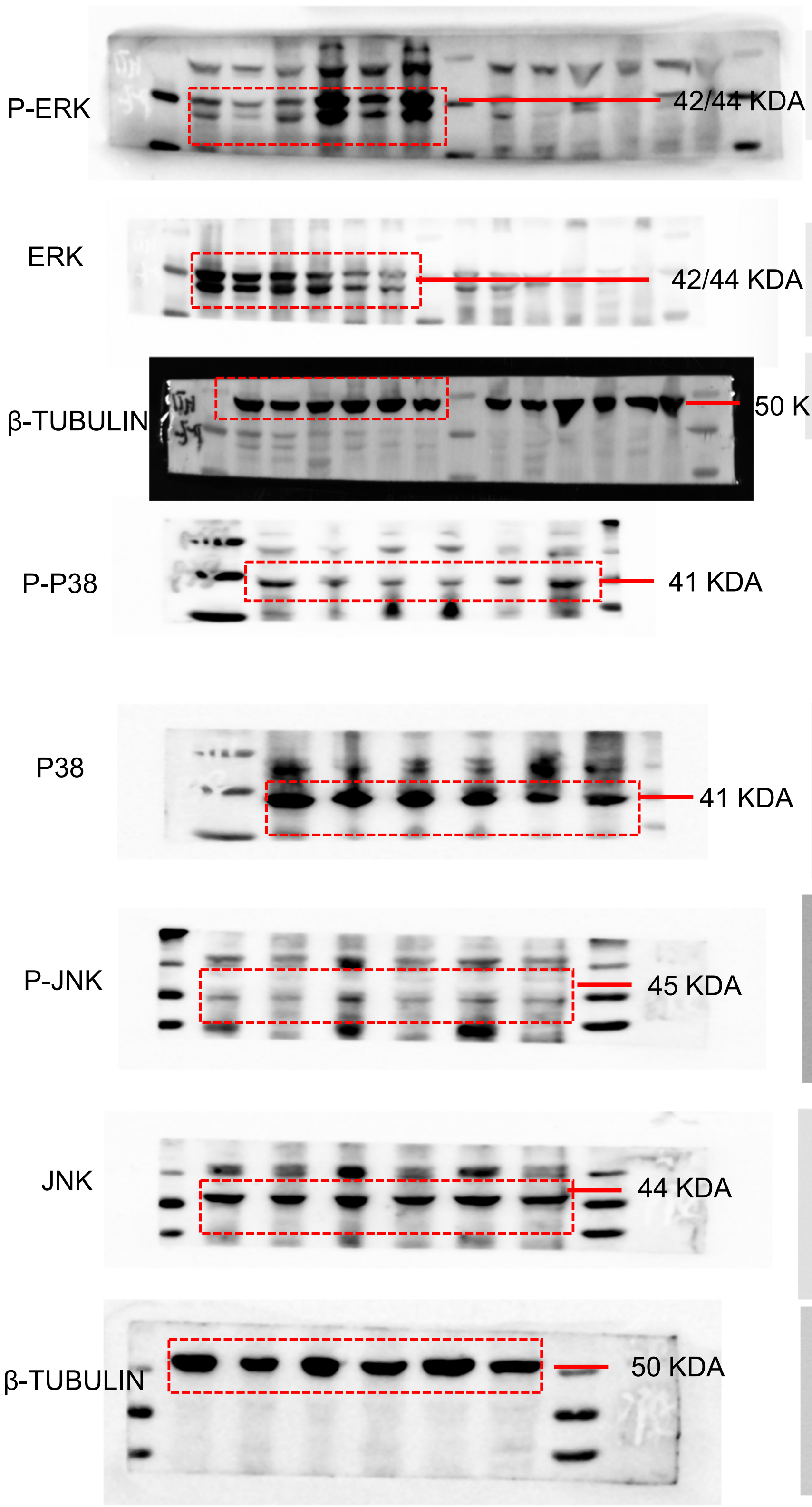

Full unedited blot for Figure 11E

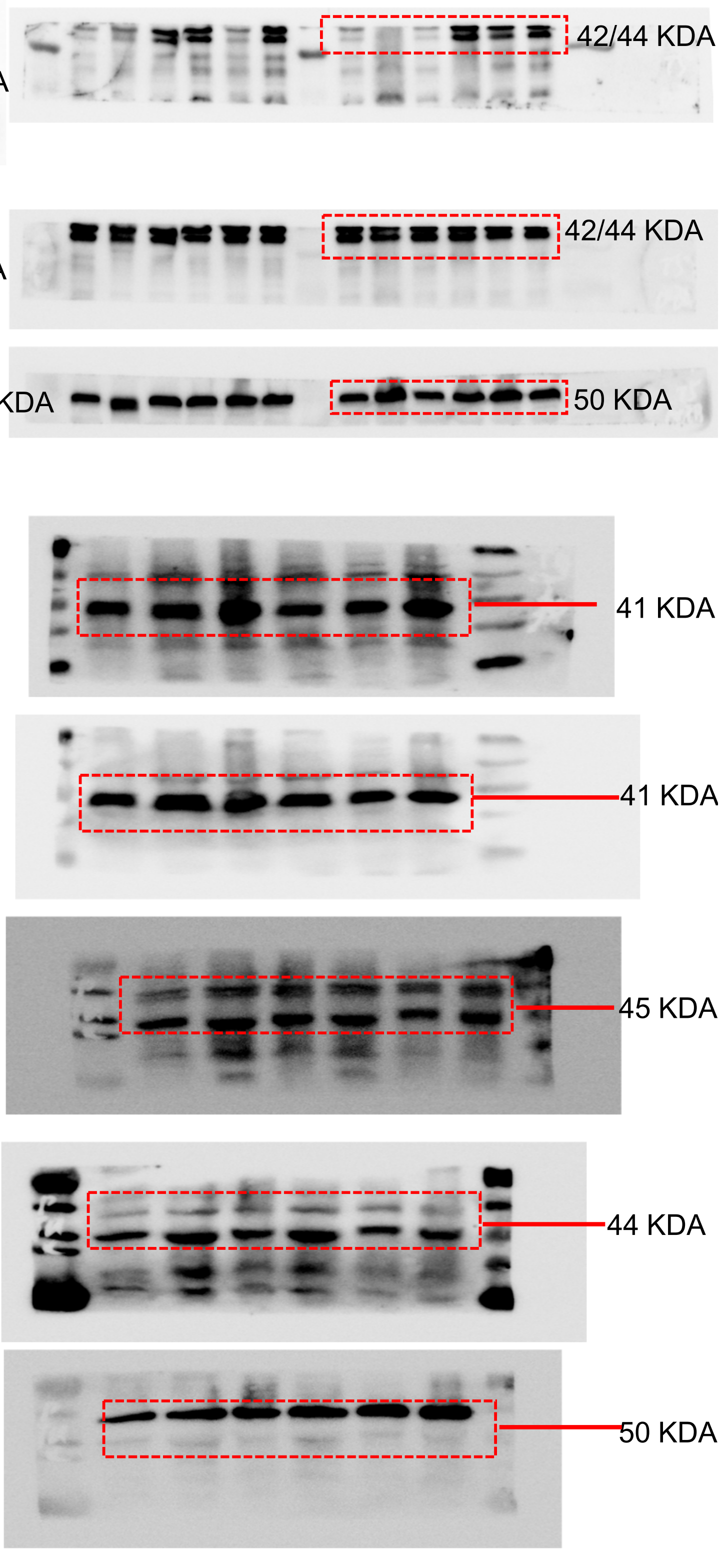

In Figure 11D and E, six samples (control and DM group) from human and mouse tissues were run in parallel gel on the same day for the P-ERK and ERK detection, and the loading control for it is shown below the blot.

Full unedited blot for Figure 11F

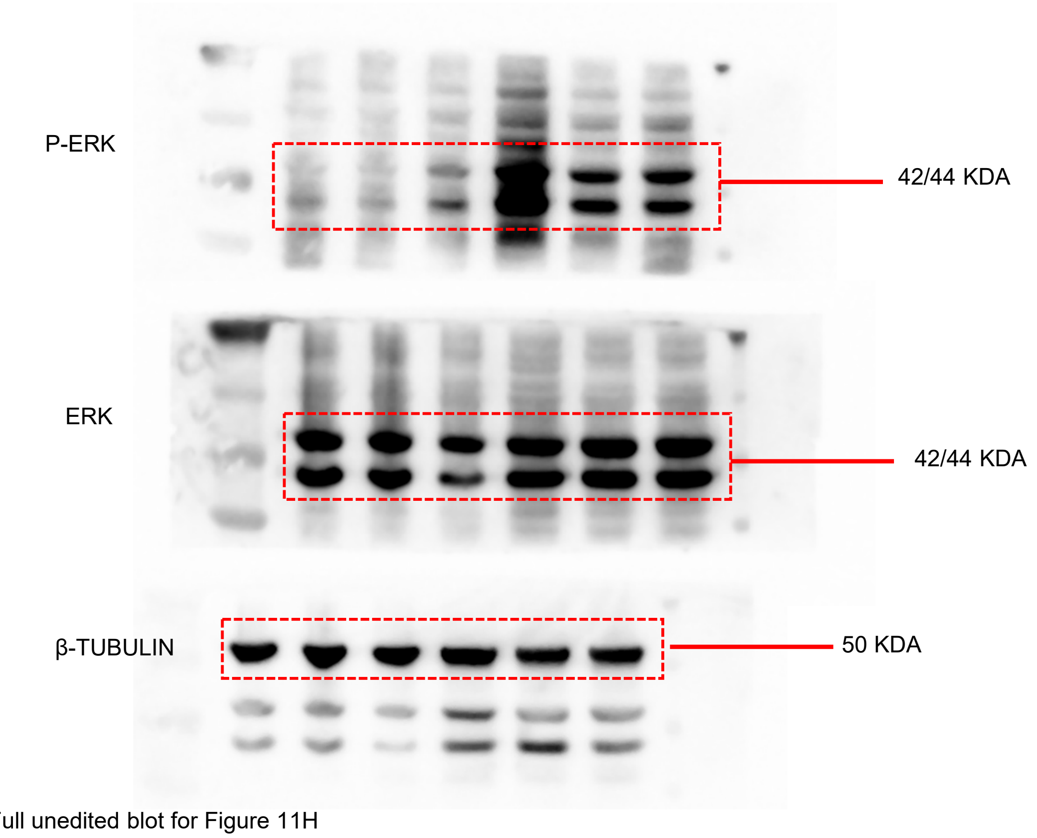

Full unedited blot for Figure 11H

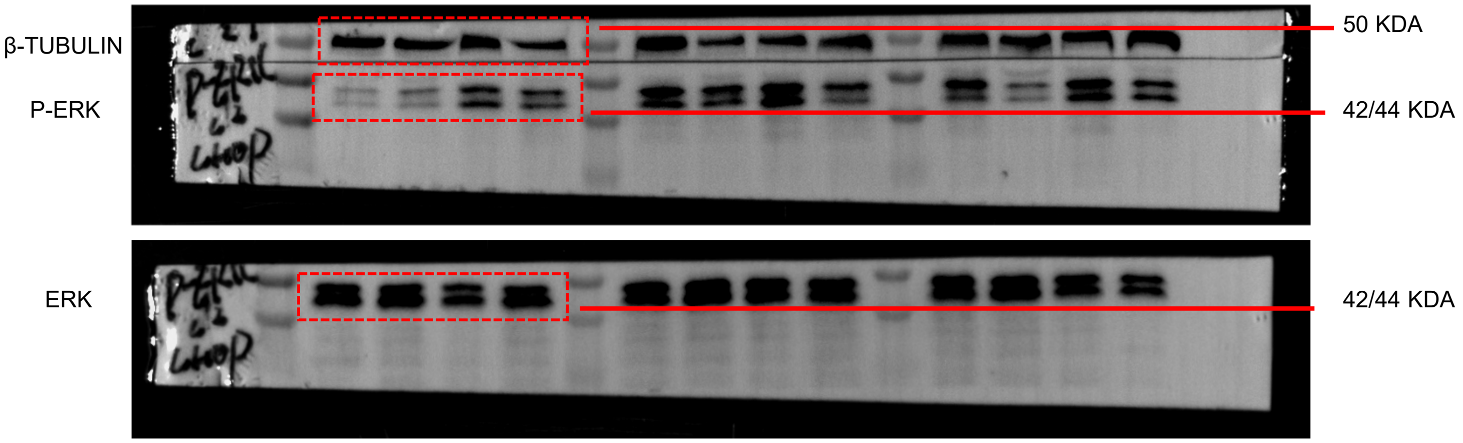

Full unedited blot for Figure 11J

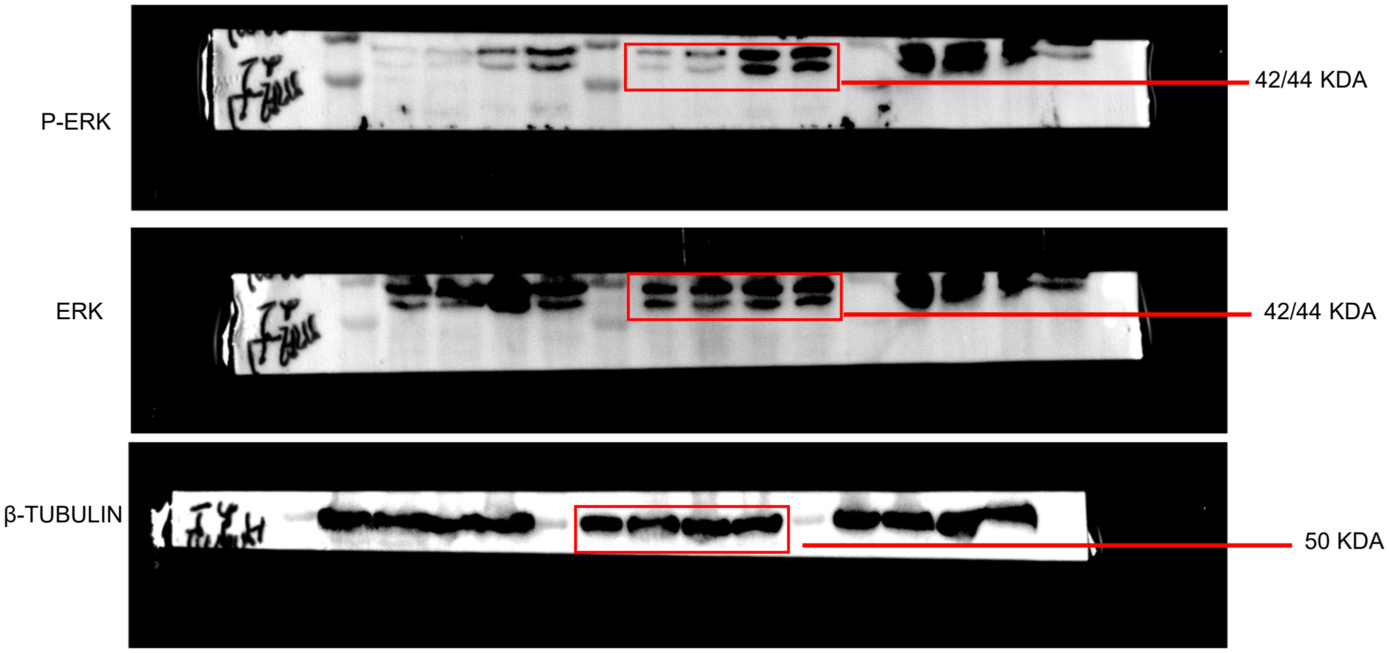

Full unedited blot for Figure 12F

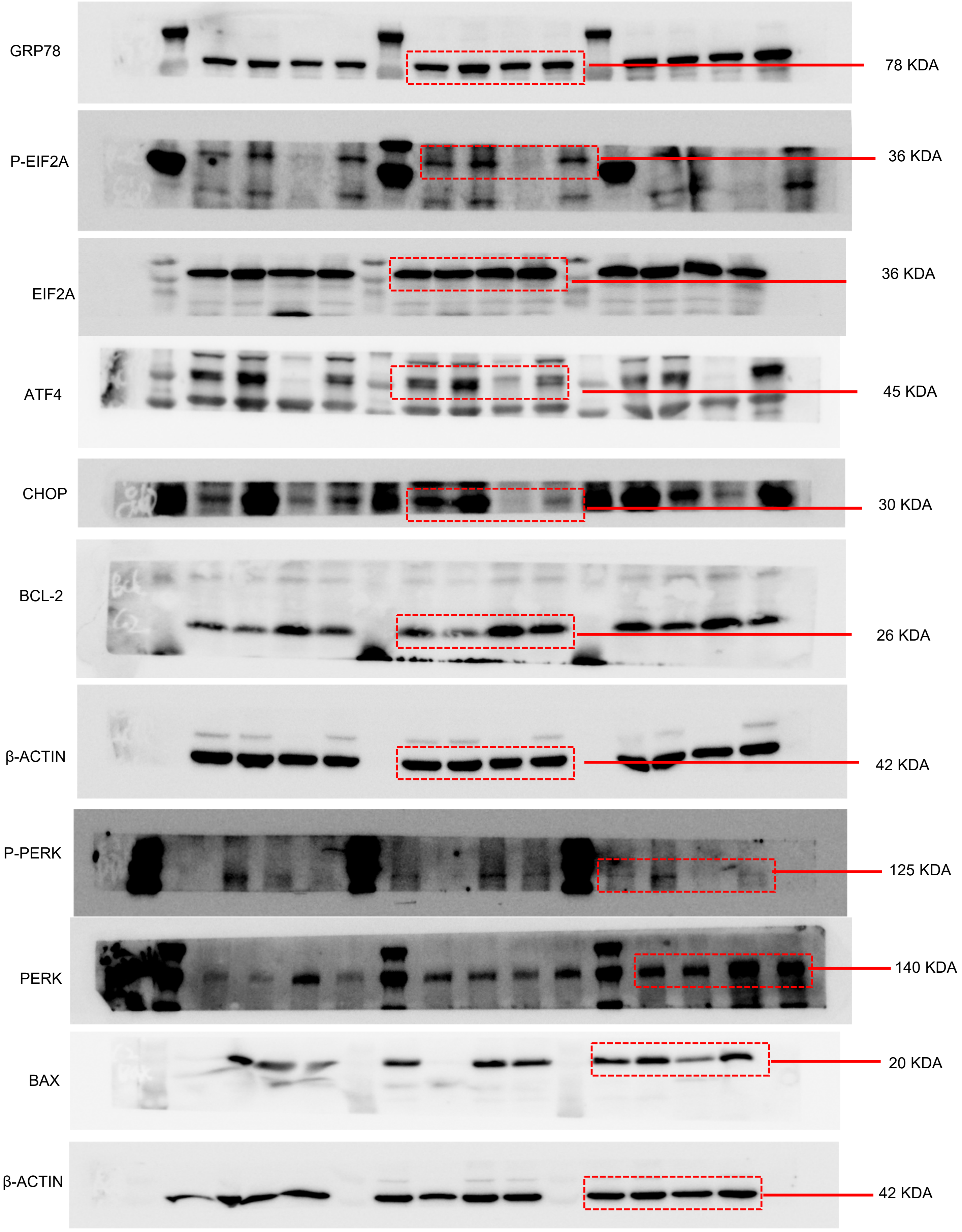

In Figure 12F, four cell samples (Glu, Glu+PD98059, Glu+MVs， Glu+MVs +PD98059) were run in parallel gel on the same day for the P-PERK, PERK and BAX detection, and the loading control for it is shown below the blot.

Full unedited blot for Figure 12G

PUROMYCIN

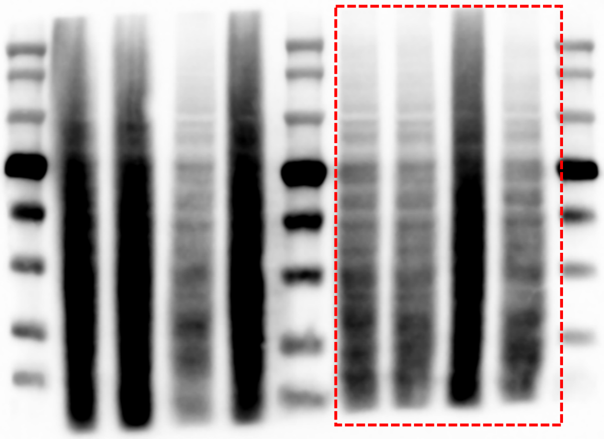

β-ACTIN

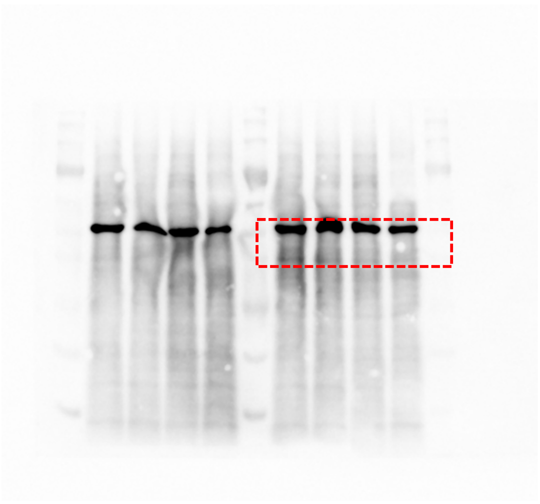

Full unedited blot for Figure 13D

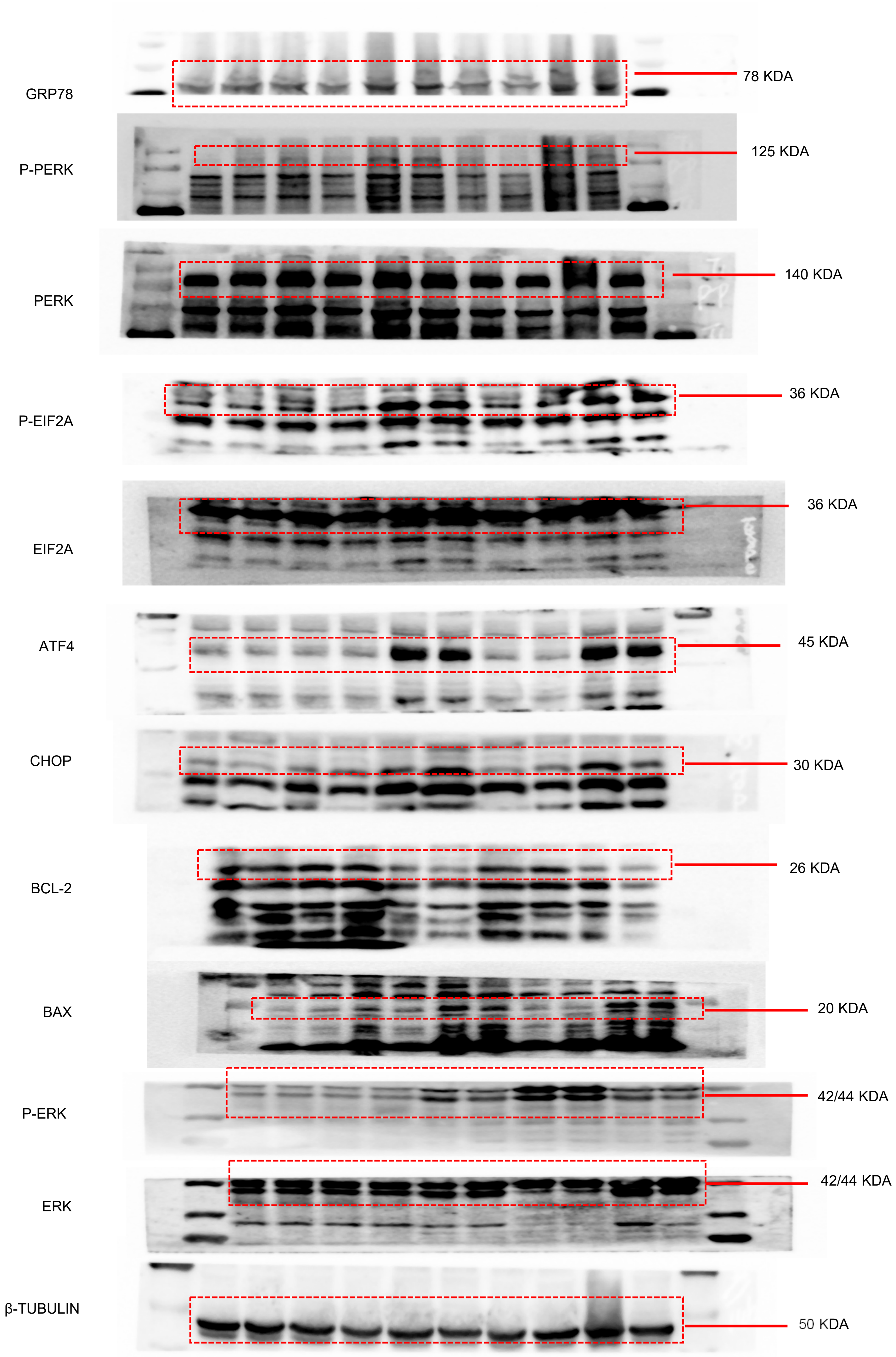

Full unedited blot for Figure 14C

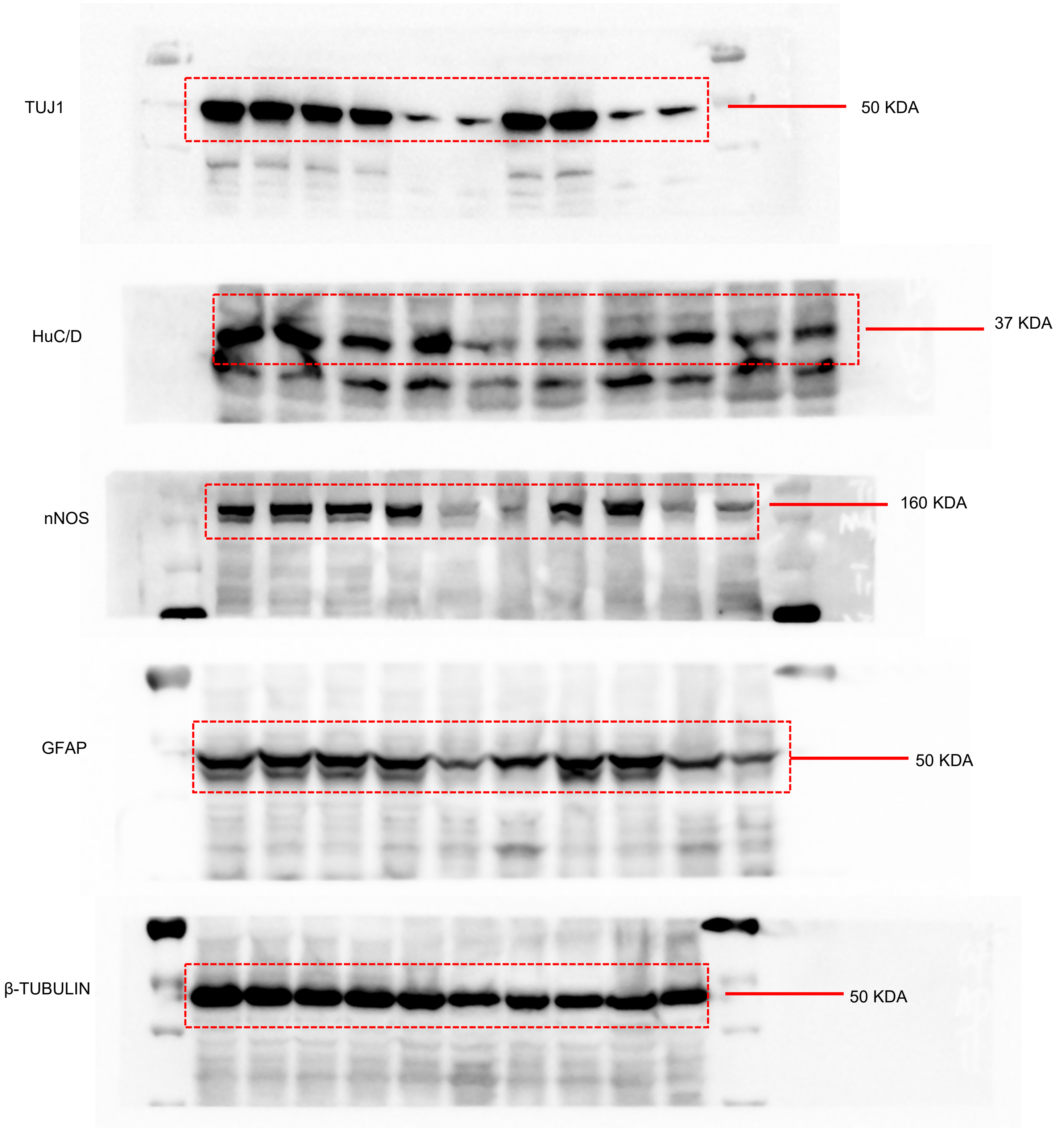

Full unedited blot for Supplemental Figure 3B

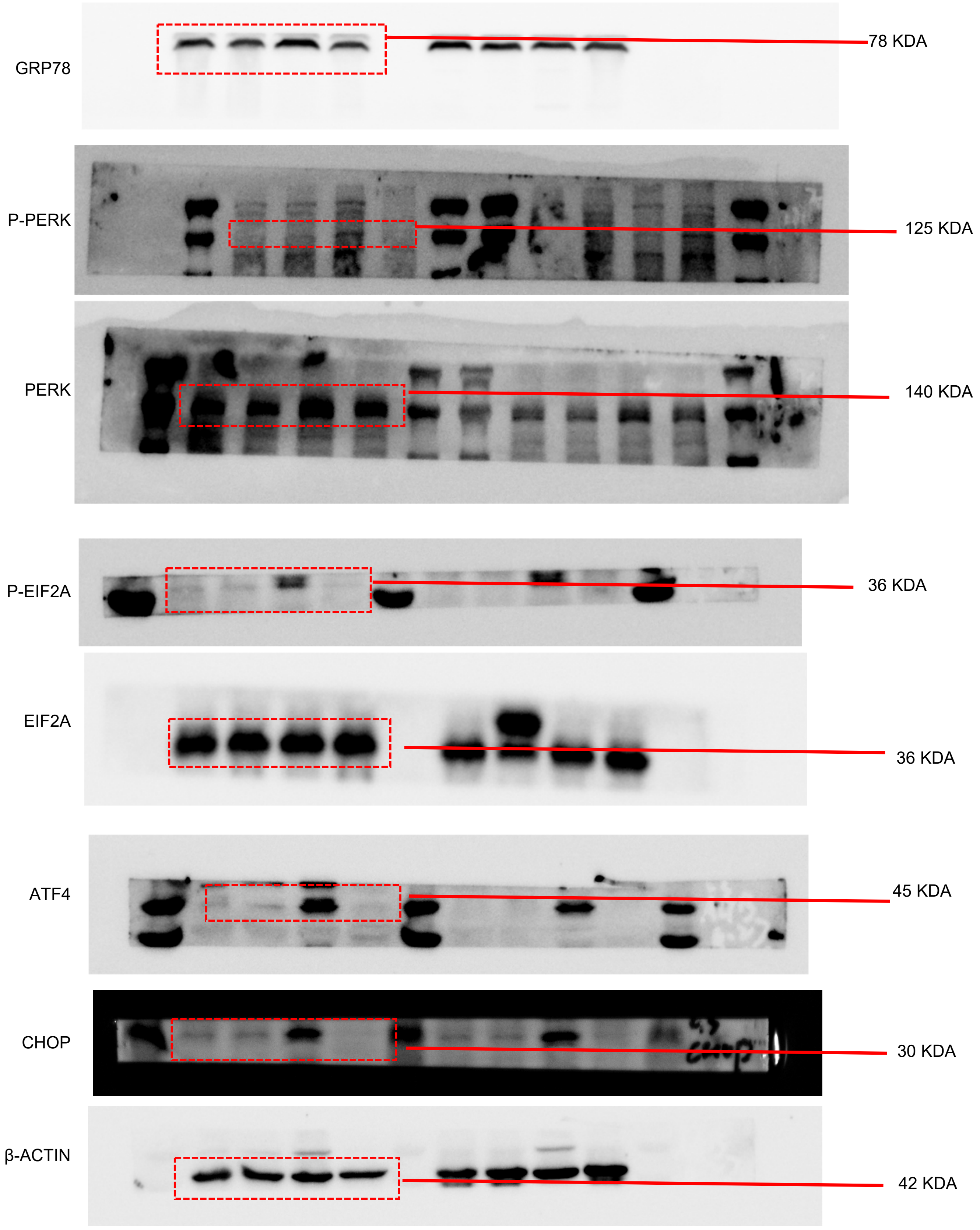

Full unedited blot for Supplemental Figure 3C

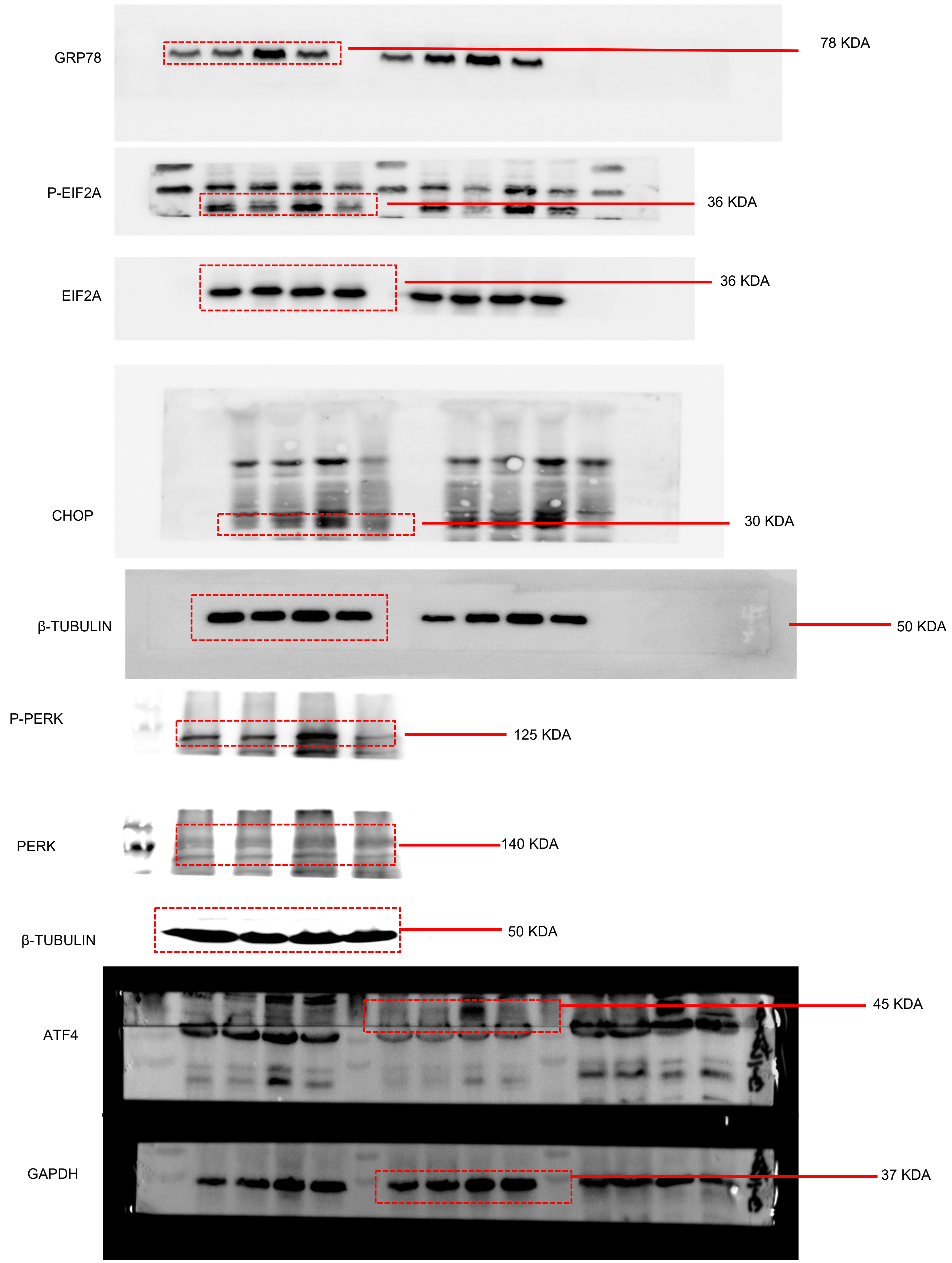

In Supplemental Figure 3C, four tissue samples (Con, Con+4-PBA, DM, DM+4-PBA) were run in parallel gel on the same day for the P-PERK, PERK and ATF4 detection, and the loading control for it is shown below the blot.

Full unedited blot for Supplemental Figure 4B

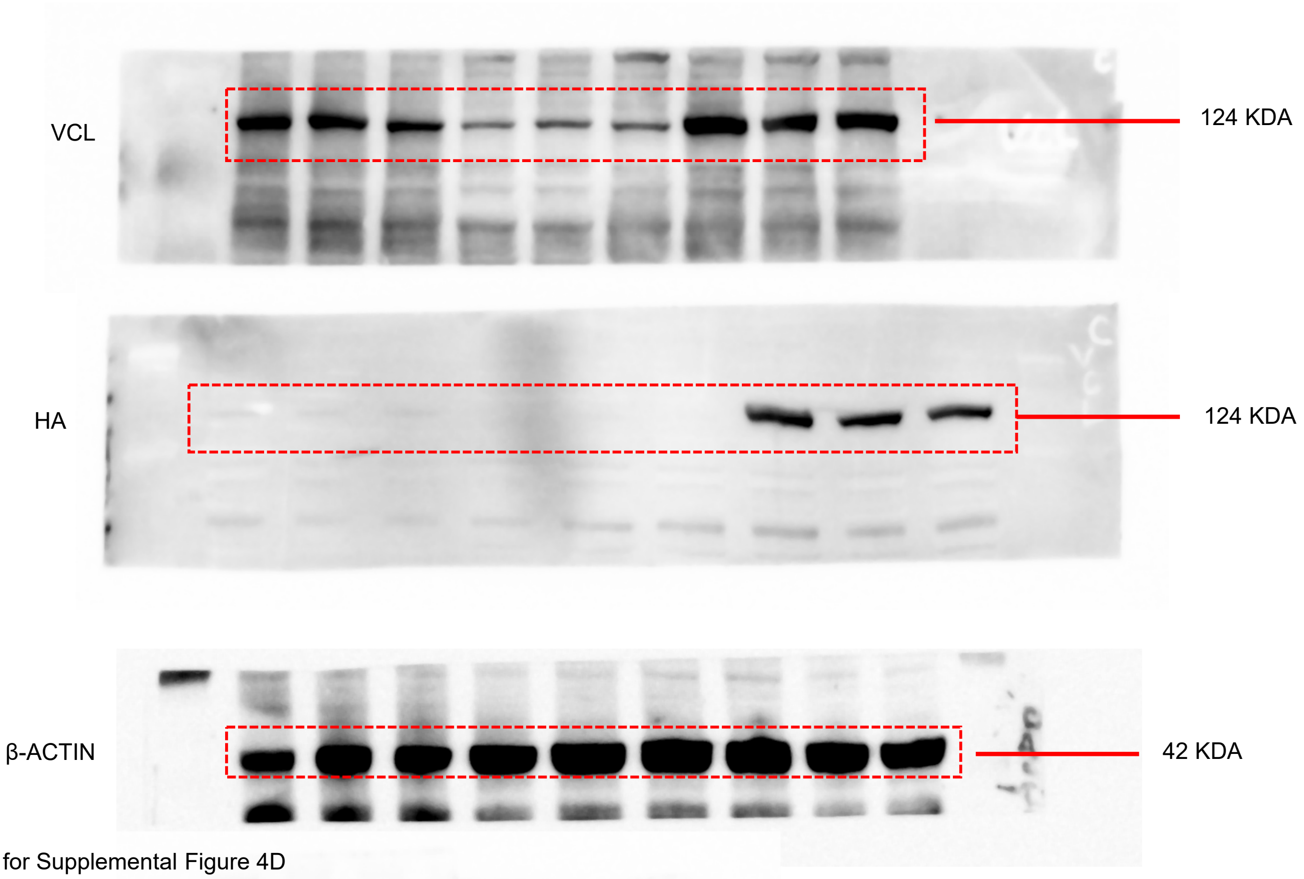

Full unedited blot for Supplemental Figure 4D

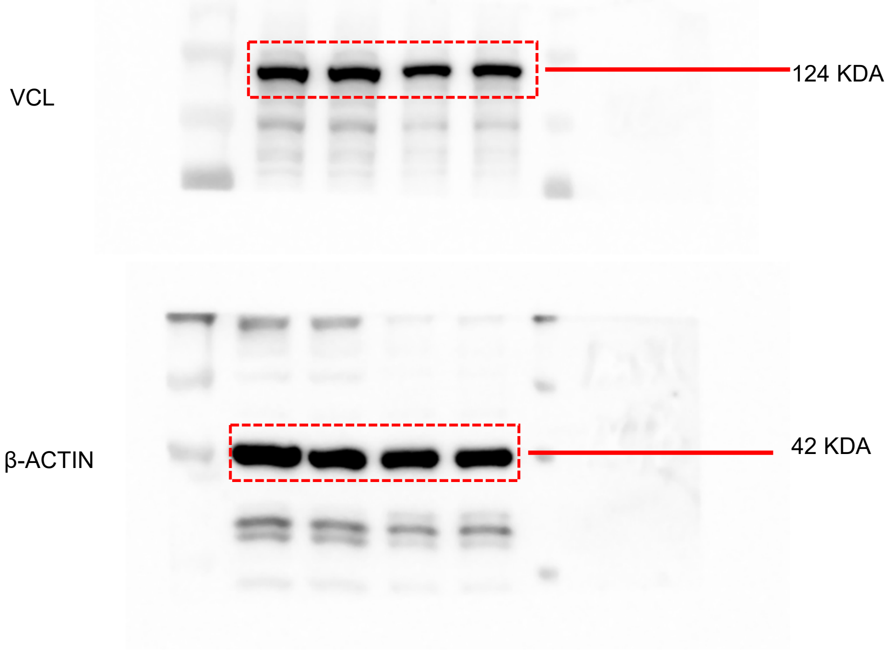

Full unedited blot for Supplemental Figure 4E

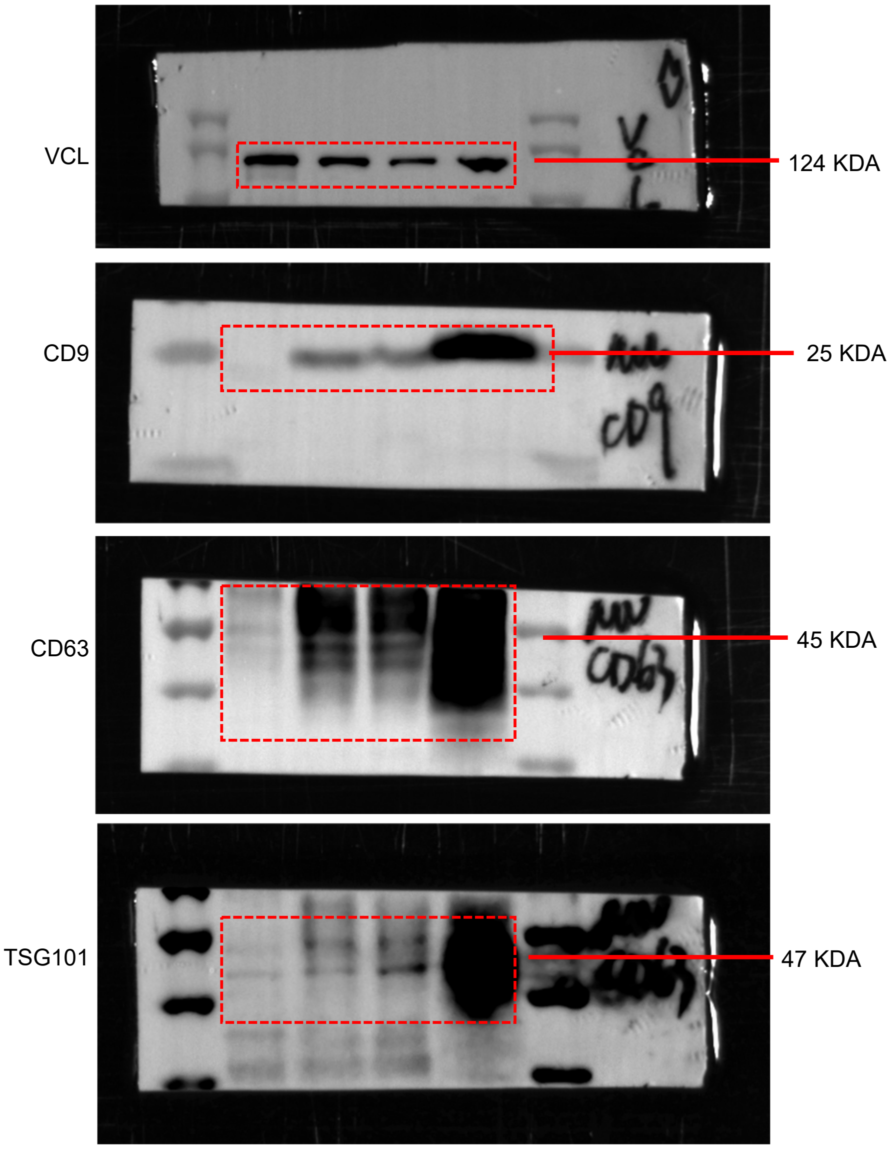

Full unedited blot for Supplemental Figure 6A

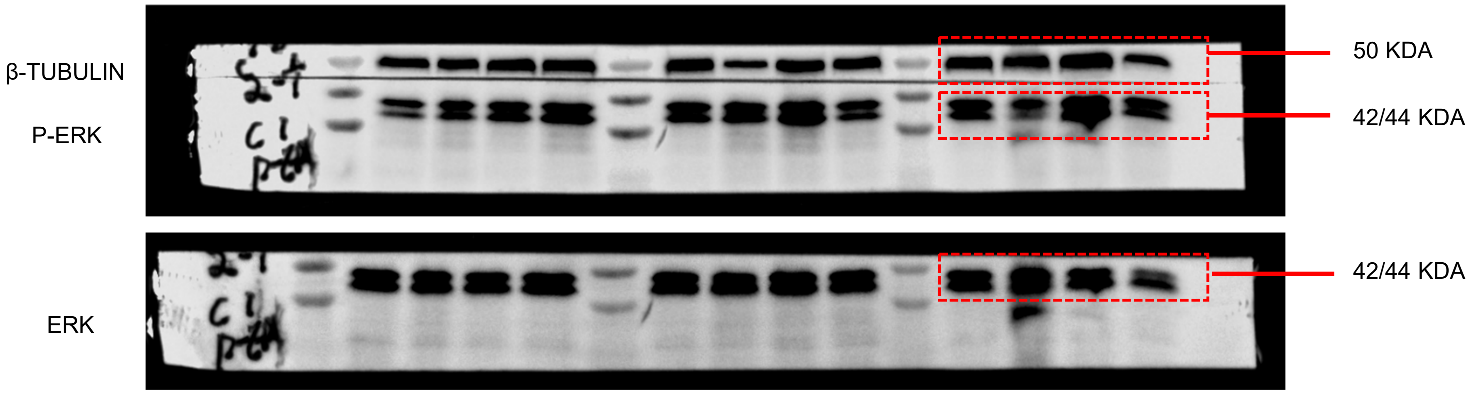

Supplement: Unedited blot and gel images [file jci-136-192437-s028.pdf]
